# Crystal Structure and Twisted Aggregates of Oxcarbazepine Form III

**DOI:** 10.1021/acs.cgd.2c00152

**Published:** 2022-05-24

**Authors:** Hector Polyzois, Rui Guo, Vijay K. Srirambhatla, Monika Warzecha, Elke Prasad, Alice Turner, Gavin W. Halbert, Patricia Keating, Sarah L. Price, Alastair J. Florence

**Affiliations:** † EPSRC Future CMAC Research Hub, University of Strathclyde, Glasgow G1 1RD, U.K.; ‡ Strathclyde Institute of Pharmacy & Biomedical Sciences, University of Strathclyde, Glasgow, G4 0RE, U.K.; § Department of Chemistry, University College London, London WC1H 0AJ, U.K.; ∥ Department of Pure and Applied Chemistry, University of Strathclyde, Glasgow G1 1XL, U.K.; ⊥ National Physical Laboratory, Teddington, Middlesex TW11 0LW, U.K.

## Abstract

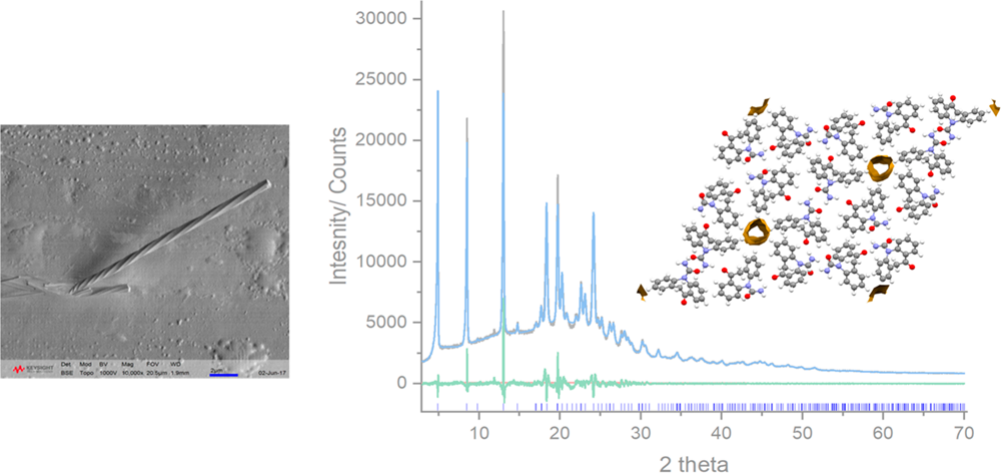

Polymorphism and crystal habit play vital roles in dictating the properties of crystalline materials. Here, the structure and properties of oxcarbazepine (OXCBZ) form III are reported along with the occurrence of twisted crystalline aggregates of this metastable polymorph. OXCBZ III can be produced by crystallization from the vapor phase and by recrystallization from solution. The crystallization process used to obtain OXCBZ III is found to affect the pitch, with the most prominent effect observed from the sublimation-grown OXCBZ III material where the pitch increases as the length of aggregates increases. Sublimation-grown OXCBZ III follows an unconventional mechanism of formation with condensed droplet formation and coalescence preceding nucleation and growth of aggregates. A crystal structure determination of OXCBZ III from powder X-ray diffraction methods, assisted by crystal structure prediction (CSP), reveals that OXCBZ III, similar to carbamazepine form II, contains void channels in its structure with the channels, aligned along the *c* crystallographic axis, oriented parallel to the twist axis of the aggregates. The likely role of structural misalignment at the lattice or nanoscale is explored by considering the role of molecular and closely related structural impurities informed by crystal structure prediction.

## Abstract 2

Twisted aggregates, crystal structure prediction, and the crystal structure of oxcarbazepine form III are detailed.

## 1 Introduction

Polymorphism is the ability of a compound to exist in different crystal packing arrangements^1,2^ and is widely observed in biological systems, foodstuffs, pigments, agrochemical products, and pharmaceuticals.^3−5^ Many important physicochemical properties of pharmaceutical solids, such as solubility, dissolution rate, flow properties, and compressibility,^6−8^ are often strongly dependent on the crystal habit and on the packing arrangement of molecules within the crystal lattice.^1^ Yet, our fundamental understanding of crystal nucleation and growth mechanisms in molecular systems remains limited and we are generally unable to predict the specific crystal attributes observed from a given set of conditions. While the monomer-by-monomer, addition-based classical nucleation theory (CNT)^9−12^ is still one of the most commonly used models and can give a reasonable fit for empirical nucleation rates, it fails to provide a quantitative/qualitative interpretation of many experimentally observed phenomena during crystallization.^13,14^ For example, several biominerals^14^ have been shown to crystallize via amorphous precursors. Such nonclassical^15^ crystallization models generally introduce an additional step or steps that precede the crystal nucleation event.^16−19^ In recent years, attempts have been made to shed light on possible intermediate stages during the early stages of crystallization while studying proteins,^16^ colloids,^20,21^ inorganic compounds,^22,23^ and small organic compounds.^24^ Monitoring and characterizing the intermediate stages of crystallization can provide new insights into the transformation kinetics during crystallization and ultimately lead to improved control of the crystallization process.

Molecular crystals are generally brittle and exhibit polyhedral shapes bounded by flat faces and sharp edges. However, in recent years, several molecular materials displaying twisted and bent morphologies have been reported.^25^ While bending of molecular crystals is generally a result of applied external force, twisted morphologies are commonly a consequence of the crystal growth conditions. Twisted crystals may present themselves as single fibers or ribbons, as elongated aggregates of multiple intertwined crystals, or as bundles in ring-banded spherulites. In the present work, we use the term aggregate or fibers to describe multiple intertwined crystals. Several examples of twisted crystals of pharmaceutical compounds have been reported, including aspirin,^26^ paracetamol,^27^ ibuprofen,^28^ and naproxen,^28^ highlighting that this may be a more common phenomenon than is currently recognized. While tracking the early crystal growth processes that result in the formation of helicoidal architectures is challenging, three general mechanisms for twisting in molecular crystals have been described. These include (1) deformation and twisting resulting from strain caused by the existence of defects that form via specific crystal growth mechanisms,^29^ (2) temperature,^30^ electrical,^29^ mechanical,^29^ and/or concentration^31^ fields that create a mechanical force acting on a growing crystal, (3) internal compositional and structural inhomogeneities that lead to a lattice mismatch with the creation of a twist moment at the growth front.^26,29^ Although all of the aforementioned types of mechanisms may be applicable under certain conditions, a lattice mismatch is most commonly invoked.

**Figure 1:**
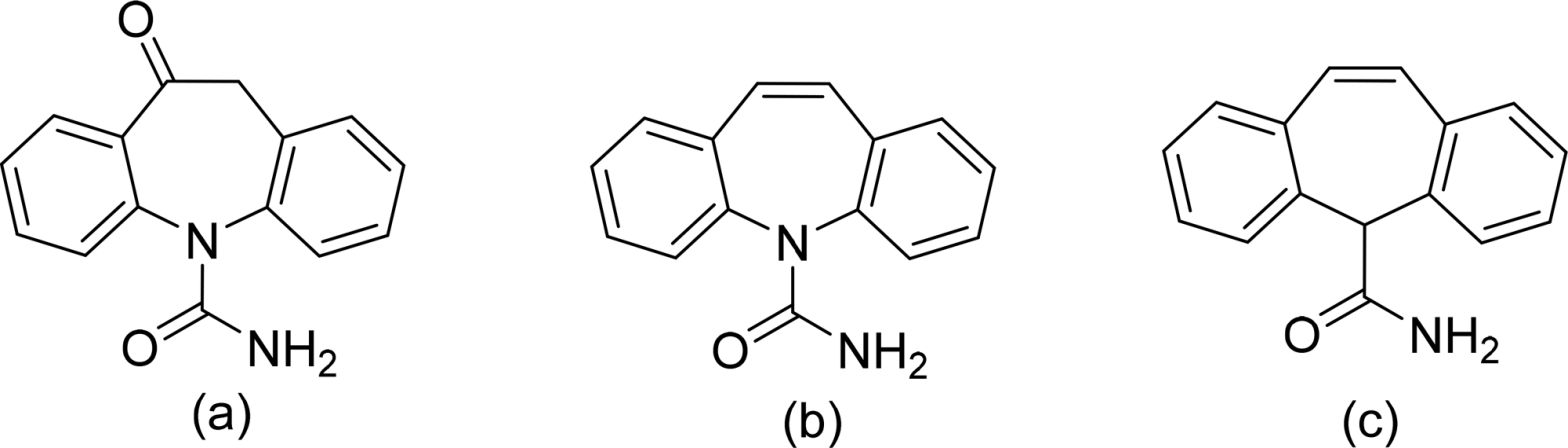
Molecular structures of (a) oxcarbazepine (OXCBZ), (b) carbamazepine (CBZ) and (c) cytenamide (CYT).

Oxcarbazepine^32^ (OXCBZ; 10,11-dihydro-10-oxo-5*H*-dibenz(*b*,*f*)azepine-5-carboxamide) is a commercially available anticonvulsant drug mostly used for the treatment of epilepsy and bipolar disorder and is a close analogue of carbamazepine (CBZ) and cytenamide (CYT). The molecular structures of OXCBZ, CBZ, and CYT are shown in Figure 1. OXCBZ is known to exist in three polymorphic forms.^33^ While the crystal structures of OXCBZ I (CSD refcode: CANDUR01) and II (CSD refcode: CANDUR02) are known, the structure of OXCBZ III has not been reported. This study describes the crystallization of OXCBZ III by multiple methods and reports the crystal structure of form III for the first time. We also demonstrate that the crystallization of OXCBZ form III from sublimation follows both a nonclassical nucleation mechanism and an unconventional growth process involving the condensation of OXCBZ vapor into droplets from which crystallites and twisted aggregates are observed to emerge. Similar but less prominent twisting is also observed in solution-grown OXCBZ III samples.

## 2 Experimental and Computational Methods

### 2.1 Solution-Based Crystallization Screening

OXCBZ, CBZ, and solvents for solution-based, solid-state screening studies were procured from Sigma-Aldrich UK, VWR Chemicals, and Acros Organics (details are given in the Supporting Information). CYT form II powder was synthesized using a modification of the method reported by Davis et al.^37^ The list of solvents used in screening studies of OXCBZ is presented in the Section S1 in the Supporting Information. Solvent selection was performed on the basis of the Strathclyde24 solvent map.^34^ All solution-based screening experiments were performed using a Crissy platform by Zinsser Analytic (see Section S1 in the Supporting Information).

### 2.2 Sublimation Experiments

Al, Ag, and Cu foils used as templates in sublimation studies were procured from Stephensons and Alfa Aesar. Cu- and Ag-coated glass substrates were fabricated (Section S2 in the Supporting Information). The details of the experimental sublimation setup^35^ are given in Section S3.1 in the Supporting Information. Typically, sublimation experiments were performed for up to 48 h. Controlled sublimation studies under high-vacuum conditions were performed using a custom-made QBox 450 system^36^ by Mantis Deposition Ltd., UK (Section S3.2 in the Supporting Information).

### 2.3 Differential Scanning Calorimetry/Thermogravimetric Analysis (DSC/TGA)

Simultaneous DSC/TGA analysis of samples was performed using a NETZSCH STA 449 F1 Jupiter thermal analyzer (NETZSCH, Germany). Samples were weighed into Al pans and crimped with a pinhole in the lid. The samples were analyzed in the temperature range of 20–300 °C at a scan rate of 10 °C/min while helium was used as the purge gas at a constant flow rate of 60 mL/min.

### 2.4 X-ray Powder Diffraction (XRPD) and Variable-Temperature XRPD (VT-XRPD)

XRPD analysis for the crystal structure determination of OXCBZ III was carried out at room temperature using a Bruker D8 Advance diffractometer operating at 40 kV and 50 mA. OXCBZ III from solution recrystallization was loaded into a 0.7 mm borosilicate glass capillary and mounted on the diffractometer operating in a capillary transmission geometry, equipped with Johansson monochromator (Cu Kα_1_ radiation, λ = 1.5406 Å) and a LynxEye detector. The detailed procedure of structure determination from powder diffraction data is presented in Section S5.4 in the Supporting Information. Variable-temperature diffraction data for OXCBZ III were collected using a Bruker D8 Discover instrument operating at 40 kV and 40 mA in Bragg–Brentano reflection geometry with Cu Kα_1,2_ radiation *(λ* = 1.5416 Å). The instrument was equipped with an Anton Paar CHC plus^+^ Cryo and Humidity chamber, LynxEye 1D detector, and 0.6 mm antidivergence slit. Data were collected from 20 to 190 °C in increments of 10 °C using a 2θ scan range of 3–40°, a step size of 0.017° 2θ, and a count time of 1 s/step. OXCBZ III obtained from sublimation was analyzed using low-background Si sample holders and measured at room temperature with data collected in the 2θ range of 3–35° with a 0.01° 2θ step size and a count time of 7 s/step. Further details are given in Section S5 in the Supporting Information.

### 2.5 Scanning Electron Microscopy (SEM)

SEM micrographs of samples were obtained with Keysight 8500B field-emission (Keysight Technologies) and JSM-IT100 InTouchScope (JEOL USA, Inc.) SEM instruments. Backscattered electron and secondary electron detectors were both utilized for data collection. Samples were adhered to Al SEM pin stubs using double-sided conductive carbon tabs and an acceleration voltage of 0.8–20 kV was used. Samples prepared using the QBox setup were analyzed with a ZEISS SUPRA 40 field-emission SEM instrument (ZEISS, Germany) using an acceleration voltage of 2.7–5 kV. Crystal features in the SEM micrographs were measured using version 1.51k of ImageJ.^38^ For pitch measurements, the method described by Fang et al.^39^ was employed.

### 2.6 Atomic Force Microscopy (AFM)

The AFM analysis of OXCBZ material resulting from sublimation experiments was performed using a Bruker Dimension FastScan AFM instrument. All images were collected under ambient conditions in PeakForce Tapping mode using Bruker ScanAsyst Air probes with the nominal spring constant *k* = 0.4 N/m and a nominal tip radius of 2 nm. The NanoScope Analysis software package (v.1.9) was utilized to apply first-order flattening to all height sensor images obtained and measure the diameter of sample features that were of interest.

### 2.7 High-Performance Liquid Chromatography–Mass Spectrometry (HPLC-MS)

HPLC-MS analysis of OXCBZ samples was performed using a dual-source LC-MS Agilent 6130 instrument (Agilent Technologies Inc., USA) with an Agilent 1200 series LC component and the UV detector set at 254 nm. An Agilent Poroshell 120 LC column (Model EC C18, dimensions 4.6 mm × 75 mm, 2.7 μm total particle size) with a mobile phase gradient from 95% water/5% acetonitrile (v/v, both containing 5 mM ammonium acetate) to 100% acetonitrile with 5 mM ammonium acetate was utilized. The mobile phase flow rate used for separation was 1 mL/min, and the column temperature was 40 °C. The injection volume employed was 10 μL with a run time for each sample of 18 min. Mass spectra were recorded in MM-ES+APCI ionization mode with both positive and negative polarity. The Agilent OpenLab CDS ChemStationEdition software (Agilent Technologies Inc., USA) was used for data collection and processing.

### 2.8 Crystal Structure Prediction (CSP), Periodic DFT-D Calculations, and Determination of Elastic Constant Components

The complete details of the CSP studies conducted for OXCBZ are given in Section S11 in the Supporting Information. As OXCBZ exists in two conformations (*syn* and *anti*) in the gas phase (Section S10 in the Supporting Information), CSP studies were carried out for both areas of conformational space separately. CrystalPredictor2.1^40^ was used to generate the hypothetical structures, allowing the amide group torsion angle to be flexible while modeling intermolecular interactions with potential-derived atomic charges and an *exp-6* form of the dispersion–repulsion (FIT) model.^41^ CSP-generated structures were subsequently refined using CrystalOptimizer2.4,^42^ using the FIT potential and distributed multipoles derived from PBE0/6-31G(d,p) wavefunctions for the intermolecular energy and the *ab initio* conformational energy penalty from the same wavefunction. The lattice energy landscape of OXCBZ is given in Figure S33 in the Supporting Information. The diagonal compression and shear components of the calculated elastic tensors^43^ of OXCBZ III, CBZ II, and CYT I were determined with DMACRYS^41^ using the same intermolecular potential model (FIT and PBE0/6-31G(d,p) distributed multipoles), assuming that the molecule was rigid. The same model was used to calculate the Γ-point phonons and estimate the Helmholtz free energies.^44^ As a cross check on the calculated relative stability of the structures, periodic DFT-D optimizations were carried out on a small number of structures with sufficiently small unit cells using CASTEP^45^ ver. 16.1.1 (Section S12.2 in the Supporting Information).

## 3 Results

### 3.1 OXCBZ Solid-State Screening and Comparison with CSP

Solid-state screening studies of OXCBZ involving solution-based crystallization and sublimation resulted in the crystallization of forms I, II, and III. The complete results from all screening experiments are presented in Tables S1–S4 in the Supporting Information. OXCBZ III was obtained from binary solvent mixtures comprised of ethanol and toluene. Sublimation studies also enabled OXCBZ III to be crystallized on all substrates, providing an additional route to produce this metastable polymorph. XRPD data showed strong similarity to data reported in the literature,^33^ enabling identification, confirmation of temperature-dependent phase transitions, and successful structure determination (details are given in Sections 3.2 and 3.3).

The CSP calculations of OXCBZ successfully predicted both OXCBZ I and II, with form I ranked as the most stable structure with the *anti* conformation and only slightly higher in lattice energy in comparison with the global minimum, which had the *syn* conformation. Five CSP structures (a96, a165, a722, a900, and a1858) containing the *anti* conformation have XRPD patterns similar to the experimental pattern for OXCBZ III (Figure S37 in the Supporting Information). CSP structure a96 lies within the range of crystal energies where ∼90% of polymorphic pairs are usually found.^46^ The relative energies of the crystal structures do vary with the computational approximations (Section S12 in the Supporting Information) but the other structures a165, a722, a900, and a1858 would be highly metastable as single-component polymorphs. The crystal-packing arrangements of these structures is similar (Table S9 in the Supporting Information) and they all contain void channels extending along the [001] direction (Figure S38 in the Supporting Information). Solvent inclusion in the channels would stabilize the structures.

### 3.2 VT-XRPD of OXCBZ III

**Figure 2:**
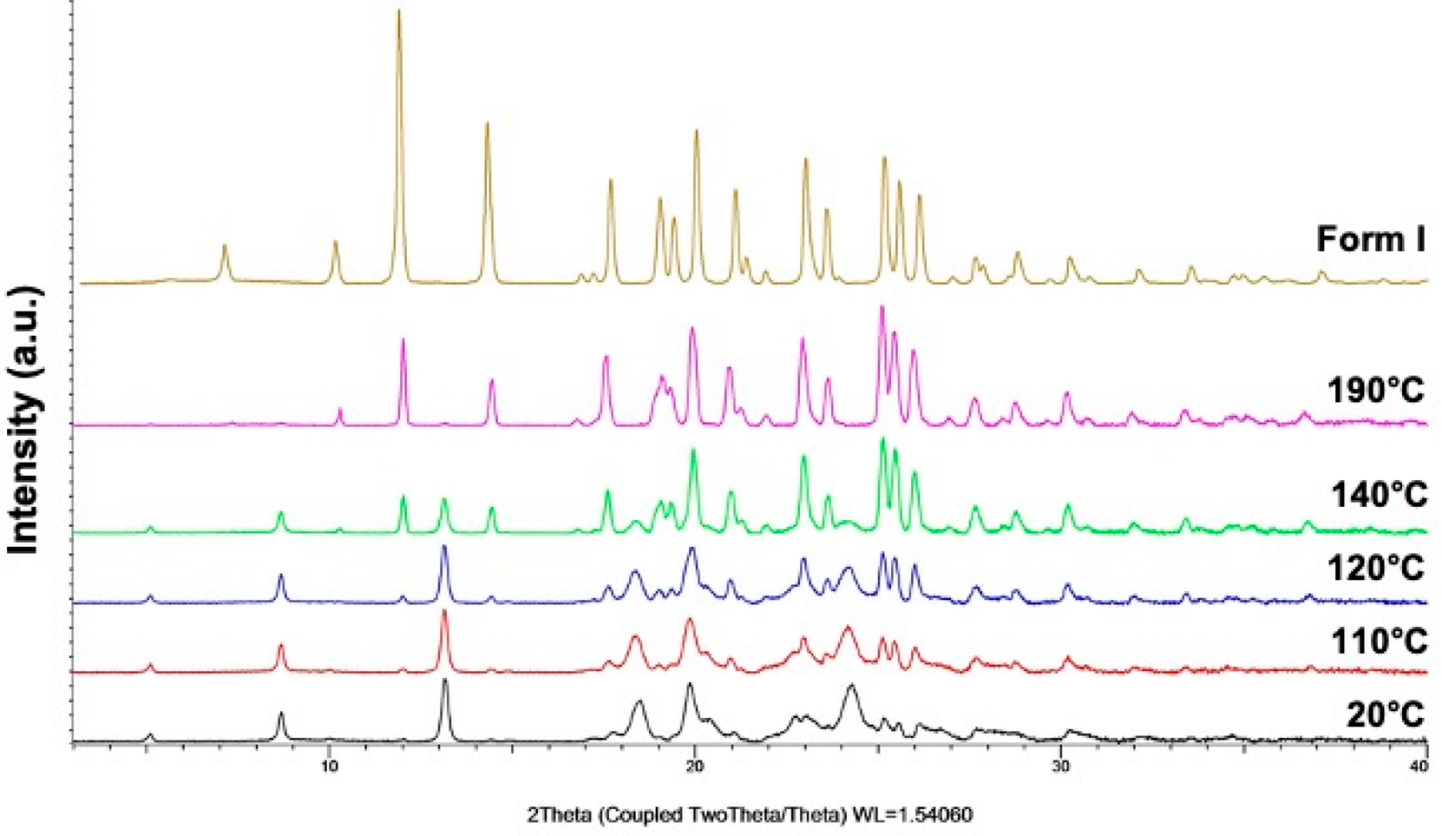
VT-XRPD analysis of OXCBZ III material obtained from an ethanol–toluene solution. A simulated pattern for OXCBZ form I (top) was calculated using single-crystal data from the Cambridge Structural Database (Refcode: CANDUR01).

OXCBZ III crystallized from solution was used to perform *in situ* VT-XRPD analysis in the temperature range 20–190 °C (Figure 2). Upon heating to 100–120 °C, OXCBZ III underwent a transformation to the thermodynamically stable OXCBZ I, evidenced by an increase in the intensity of the diffraction peaks present between 12−14.4° 2θ, which are both characteristic of form I. The transformation to OXCBZ I was completed by 190 °C, consistent with a DSC/TGA analysis (Figure S20 in the Supporting Information) and previously reported data for OXCBZ III.^33^

### 3.3 Crystal Structure Determination of OXCBZ III

**Figure 3:**
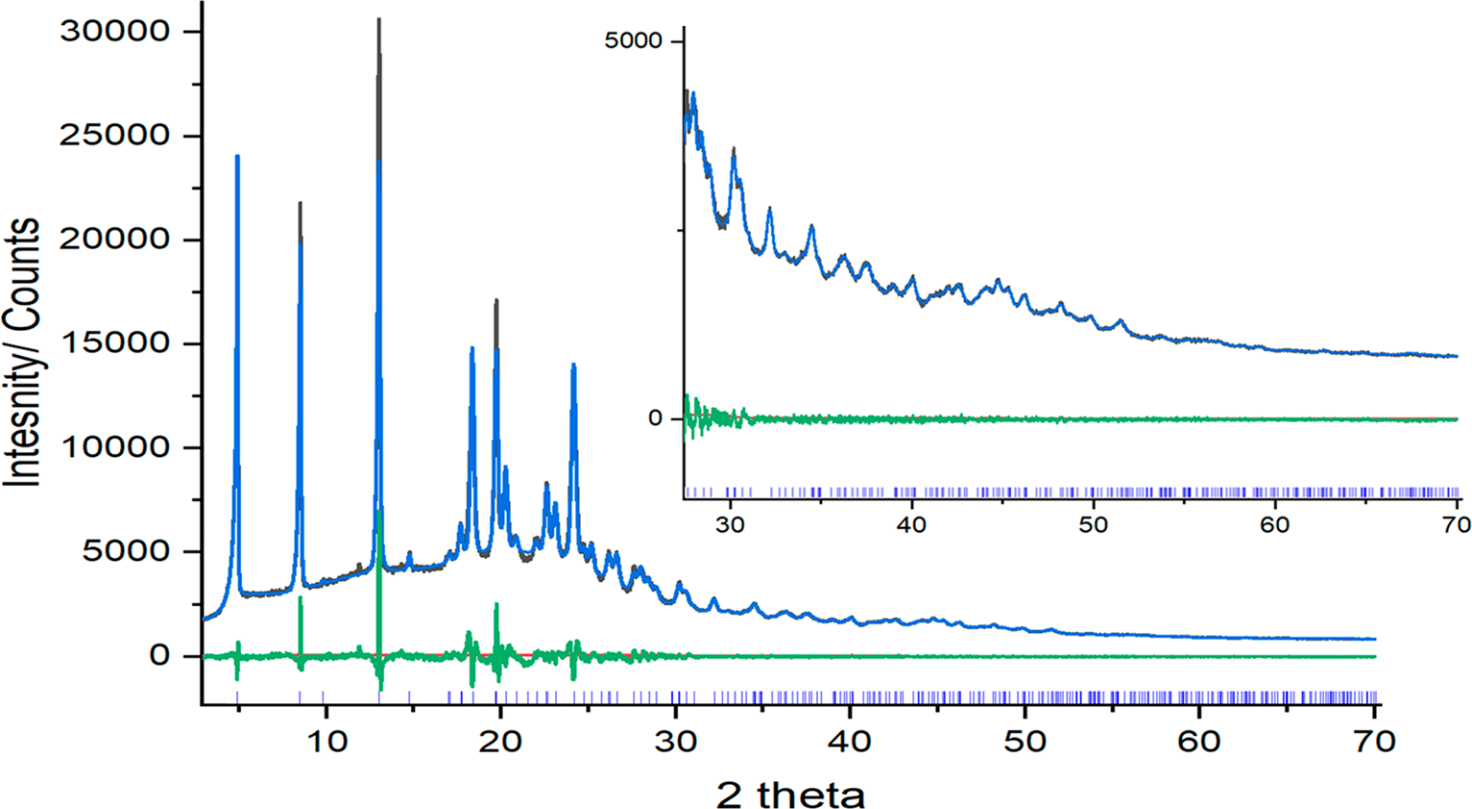
Final Rietveld plot of OXCBZ III in the range 3–70° 2θ (inset: 25–70° 2θ). The black (Y_obs_) and blue (Y_calc_) lines represent the observed and calculated patterns in OXCBZ III, respectively. The bottom green line (Y_obs_ – Y_calc_) represents the difference between the observed and calculated patterns. The blue tick marks at the bottom correspond to reflection positions in OXCBZ III.

**Figure 4:**
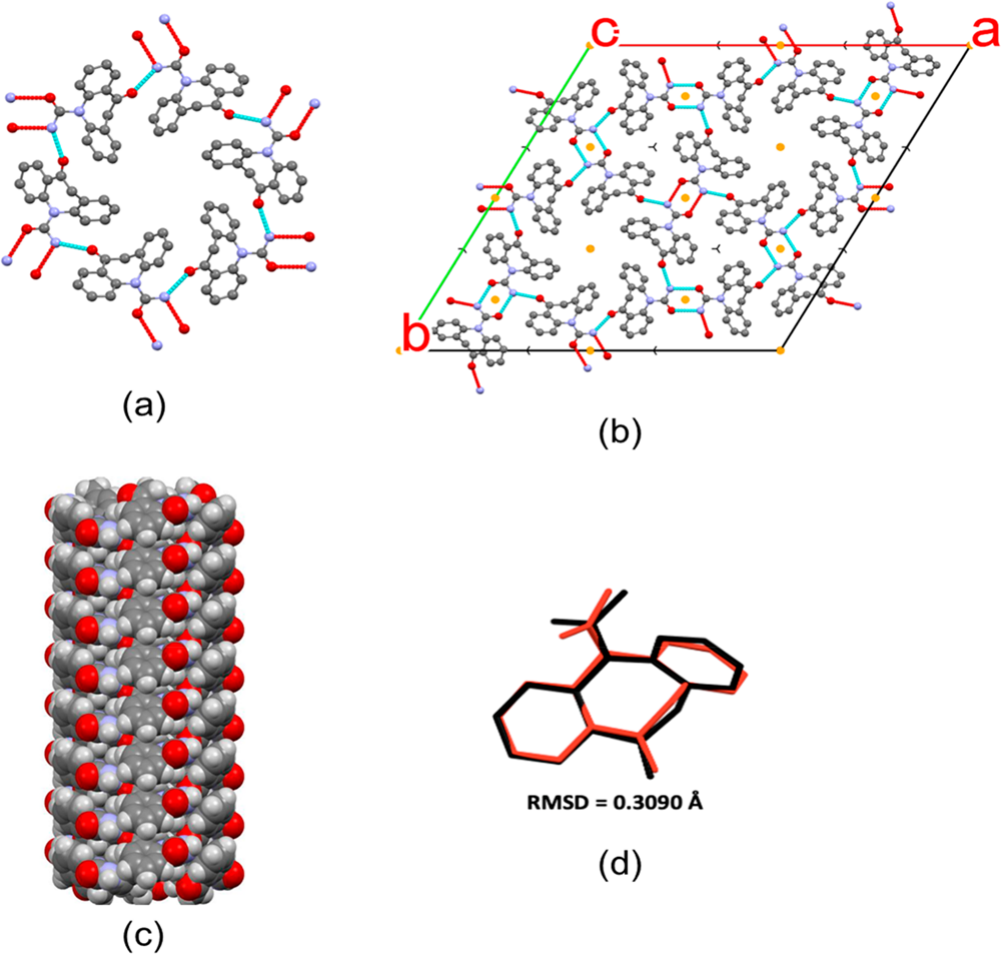
(a) *R*_6_^6^(48) type hydrogen-bonded arrangement around the channels in OXCBZ III. (b) Crystal-packing arrangement in OXCBZ III viewed along [001]. The yellow features in the packing diagram indicate the presence of inversion centers. (c) Space-filling model of the columnar stacking of motif in (a) along the *c* axis in OXCBZ III. (d) Overlay of the molecular conformation of the refined, experimental OXCBZ III structure (red) with the predicted CSP structure a96 (black).

Repeated attempts to obtain suitable single-crystal samples to allow structure determination using single-crystal diffraction were unsuccessful, and so a structure determination of OXCBZ III from powder diffraction data was pursued. Pawley profile fitting^47^ of the XRPD data (Figure S15 in the Supporting Information) confirmed that OXCBZ III crystallizes in the *R*3̅ space group. A closer inspection of the powder diffraction data suggested the presence of minute amounts of OXCBZ I. A Pawley fitting showed that the unit cell parameters of CSP a96 and a900 fit the experimental data for OXCBZ III almost equally well and are significantly better than the rest of the candidate structures. As the lowest-ranked candidate in the lattice energy landscape, structure a96 was identified as the most suitable candidate structure for OXCBZ III. The crystal structure of OXCBZ III was solved using real-space methods^48^ implemented in DASH.^49^ The refined lattice parameters, space group, and OXCBZ molecular geometry from structure a96 were used as inputs in the structure solution attempt. The positions and orientations of the molecules in the unit cell as well as the rotations around single bonds were varied subject to a Mogul distribution bias.^50^ The best solution from the simulated annealing runs was used in a restrained Rietveld refinement^51,52^ in TOPAS.^53^ Standard restraints were applied to bond lengths, bond angles, and planarity. The final Rietveld fit of the refined structure to the XRPD data is shown in Figure 3. A comparison of the molecular conformations of the CSP structure a96 and the experimentally determined OXCBZ III structure is shown in Figure 4 (Table S5 in the Supporting Information). While the crystal structure of OXCBZ III is isostructural with CBZ form II^54^ and CYT form I,^55^ the hydrogen-bonding arrangement observed for OXCBZ III displays an additional N–H···O=C H-bonding interaction arising from the additional hydrogen bond donor in OXCBZ (Figure 4).

### 3.4 SEM Analysis

#### 3.4.1 Solution-Grown Crystals

**Figure 5:**
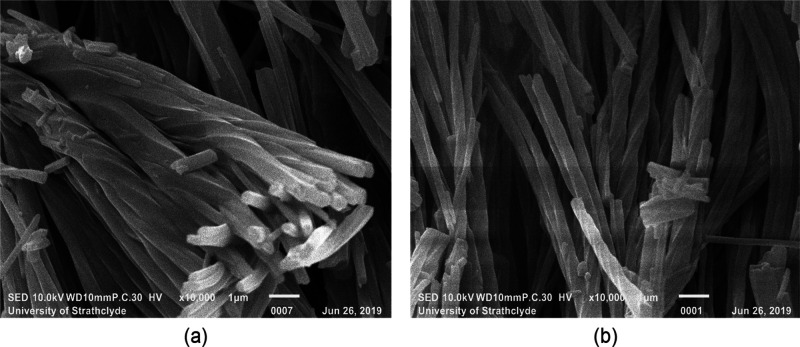
SEM micrographs (a and b) of solution-grown OXCBZ III showing the clear presence of twisted morphologies. The scale bar is 1 μm for both micrographs.

**Figure 6:**
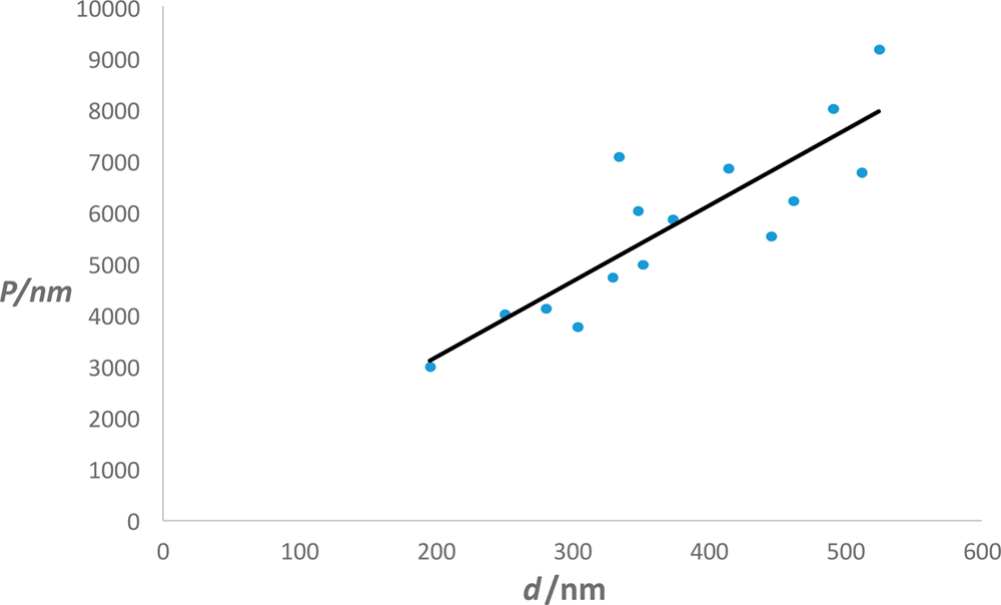
Correlation between pitch, *P*, as a function of aggregate diameter, *d*, measured from a sample of solution-grown OXCBZ III twisted aggregates.

SEM micrographs of representative samples of solution-grown OXCBZ III are shown in Figure 5. Multiple twisted features can be seen to emerge from the same central bundle (Figure 5a), indicating that the solution-grown crystalline OXCBZ III comprises structures that can be described as helical or twisted polycrystalline aggregates.^29^ These images clearly show that the OXCBZ III aggregates are twisted along their respective lengths. The diameter and length of individual aggregates are in the ranges ∼160–540 nm and ∼1.6–9.5 μm, respectively. These parameters both vary considerably between aggregates, though the width of any particular aggregate is relatively uniform along its length. The extent of the twist was assessed by measuring the diameter, *d*, the angle of the twist at different positions along several aggregates, and the pitch of the twist, *P*, defined as the length corresponding to a rotation of 360° (Figure 6). The pitch is generally observed to increase with the width of the aggregate. While twisted OXCBZ III aggregates were observed from solution-derived samples, nonaggregated, straight crystals were the most prevalent in all samples from solution recrystallization. The morphology of nontwisted, nonaggregated crystals shows a close comparison to the hexagonal needle morphology calculated from the OXCBZ III crystal structure (Section S13.3 in the Supporting Information).

#### 3.4.2 Vapor-Grown OXCBZ III

**Figure 7:**
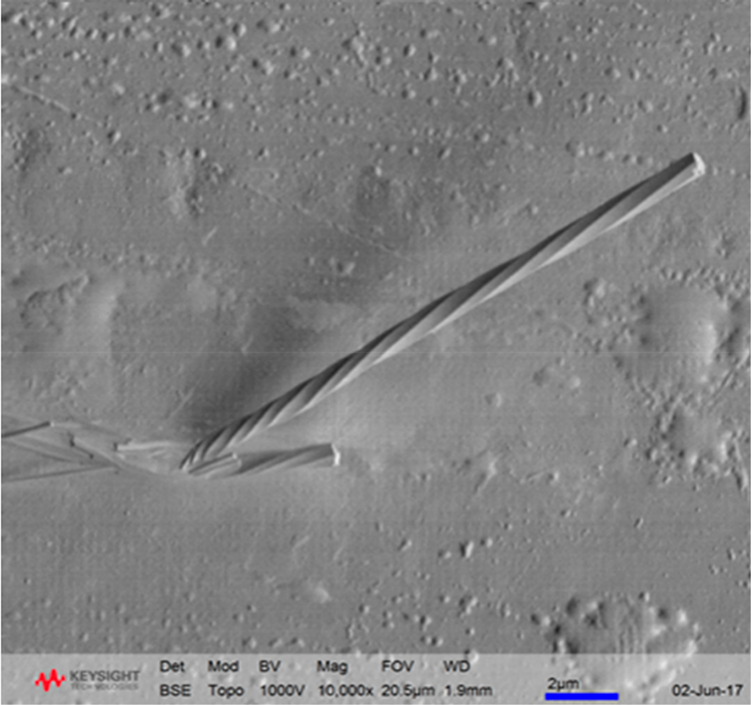
Representative SEM image depicting a twisted morphology in a crystal of OXCBZ III grown via sublimation onto Ag foil.

Sublimation experiments (Section 3.1) with growth from the vapor phase onto a range of surfaces/templates consistently resulted in the formation of OXCBZ III, but we note that samples were often found to also contain the thermodynamically stable form I. The complete set of results is presented in Table S3 in the Supporting Information. The formation of OXCBZ III was largely independent of the type of surface used (metallic foil or metal-coated glass) or the nanoscale roughness of each surface (see Section S2 in the Supporting Information). Further sublimation experiments using the QBox 450 system to explore the potential effect of variable deposition rate were unable to replicate form III under the conditions tested and yielded only OXCBZ I. This may be due to the assumed lower deposition rates and thermal driving force achieved in QBox, favoring the more stable form. Full results from QBox experiments are provided in Table S4 in the Supporting Information. An SEM analysis of vapor-grown OXCBZ III (Figure 7) indicated the presence of a helical or twisted morphology very similar to that observed in solution-grown OXCBZ III. From Figure 7 it can be noted that the diameter of the crystal at the substrate end is ∼170 nm. The diameter increases to ∼750 nm when the length of the crystal is ∼1.52 μm. At lengths greater than 1.52 μm, though the diameter of the crystal remains almost constant (∼750 nm), the pitch gradually increases continuously as the length of the crystal increases.

**Figure 8:**
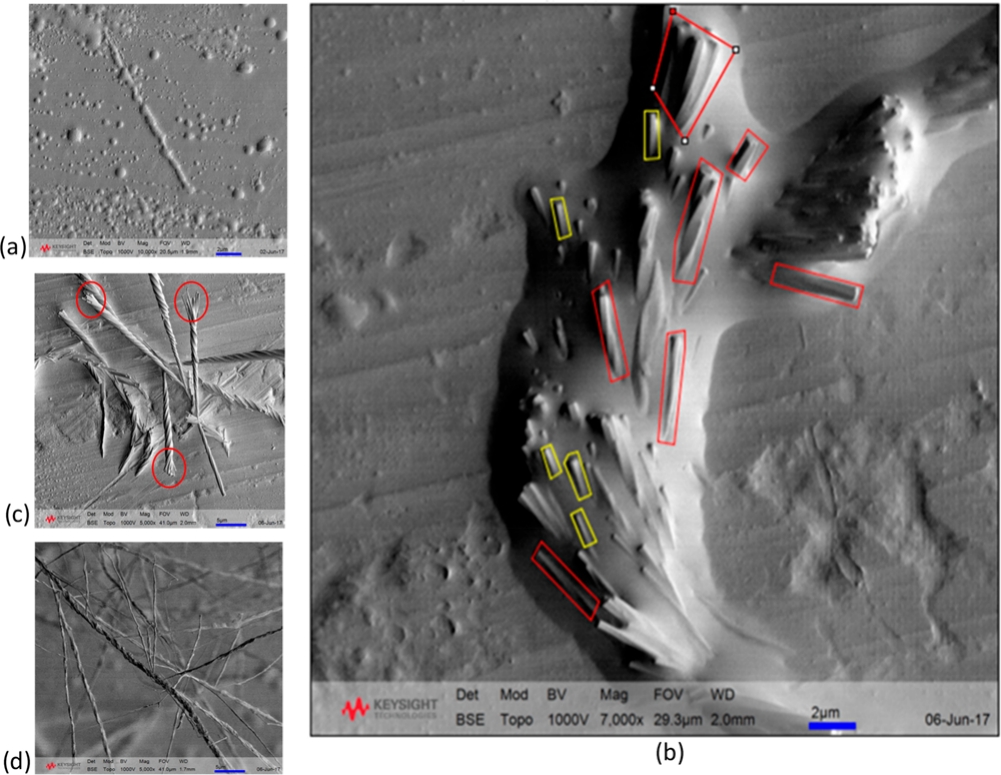
SEM micrographs from samples on Ag foil showing (a) the distribution and coalescence of surface droplets after 16 h of OXCBZ sublimation and (b) the emergence of needlelike nanocrystals from the droplets after 40 h of sublimation. Yellow boxes highlight crystals that are not twisted, while red boxes indicate evidence of twisting. (c, d) Aggregates or bundles of twisted OXCBZ III crystals after 40 h showing a range of crystal widths and strand splitting (c, red circles). Scale bars = 2 and 5 μm for (a, b) and (c, d), respectively.

Individual particles obtained from sublimation experiments exhibited cross sections with diameters in the range of ∼60–650 nm. As with the solution-grown samples, the cross-section sizes of the sublimation-grown OXCBZ III materials were relatively constant along their respective lengths. An SEM analysis following prolonged sublimation (Figure 8c) revealed the occurrence of twisted aggregates splitting into multiple single strands. This provides further evidence that the vapor-grown OXCBZ III material comprises helical polycrystalline aggregates with multiple intertwined crystals.

The observed twist in vapor-grown OXCBZ III was less uniform than in solution-grown crystals, with a variation in the extent of the twist observed across different aggregate lengths, *l* (Figure S7 in the Supporting Information). The fact that the pitch of these aggregates increased with increasing length indicated renormalization of the pitch:^56^ i.e., untwisting of the twisted aggregates as they grow longer. The angle of the twist was also found to vary between different individual aggregates produced from the same experiment. These observations indicate that the crystalline aggregates also display sensitivity to small, local changes in heat and mass transfer and driving force at the nanoscale under the individual experimental conditions. The relative frequency of twisted aggregates in OXCBZ III samples prepared via sublimation was found to be significantly greater than that in solution-grown samples.

**Figure 9:**
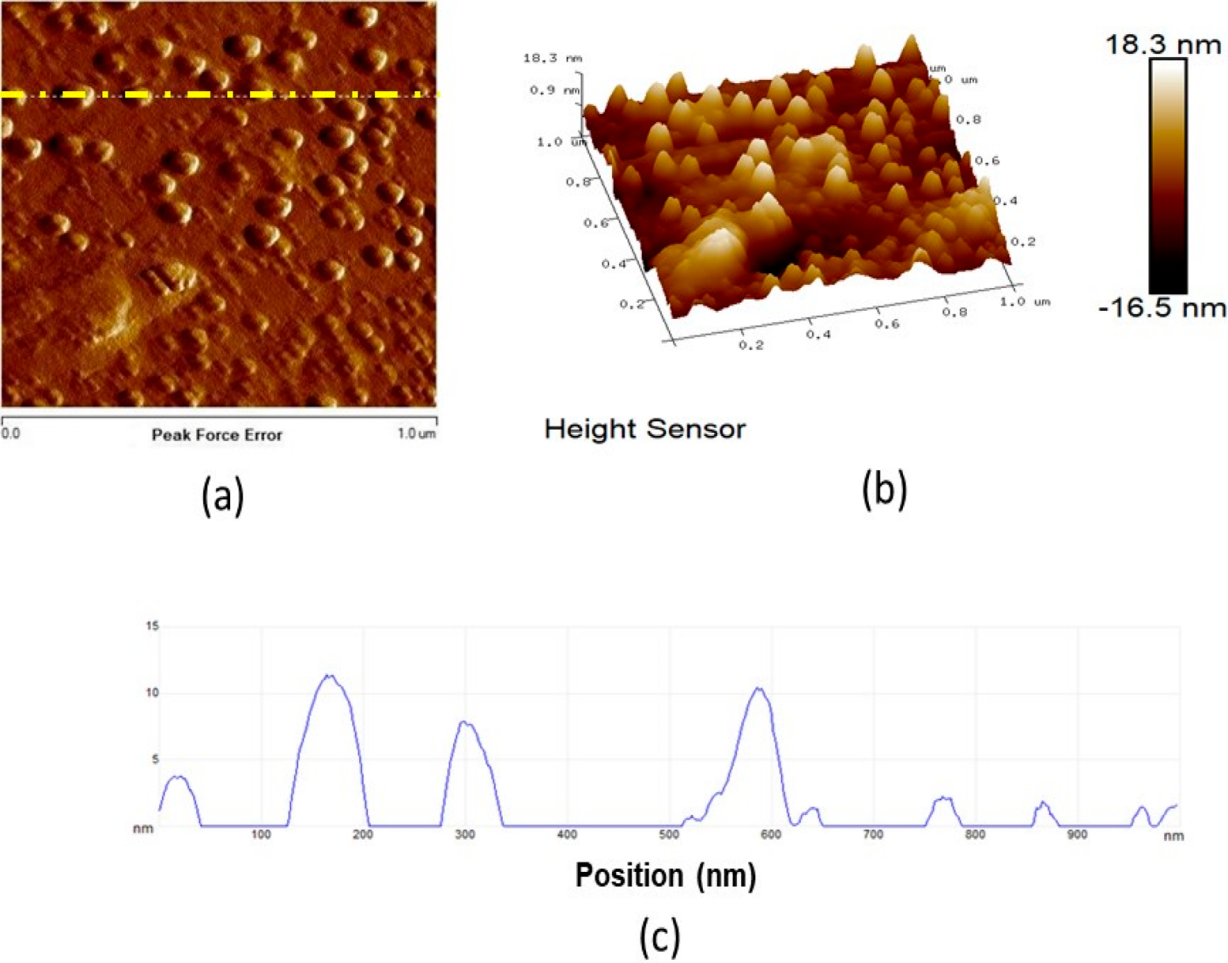
AFM scans showing the initial droplets formed on Ag foil exposed to OXCBZ vapor (a, b). (c) Corresponding cross section of height indicated by the dashed yellow line in (a). Image collection occurred using a scan size of 1 μm × 1 μm after 2 h of OXCBZ sublimation.

An SEM analysis of the time-dependent vapor deposition of OXCBZ onto Ag foil is shown in Figure 8, and the images capture different time points during the crystallization of OXCBZ III from the sublimation experiments. These results show an unconventional crystallization mechanism. Within the first 1 h of OXCBZ sublimation, spherical droplets of a condensate were observed on the Ag foil. These droplets were imaged using AFM (Figure 9) and exhibited a mean diameter of 70.18 ± 8.78 nm (*n* = 30 droplets) after 1 h of sublimation. The height of protrusion of the droplets from the foil was less than 15 nm. Raman spectra collected from a number of droplets in the low-frequency region^57,58^ of 10–400 cm^–1^ exhibited broad peaks similar to those of an amorphous OXCBZ reference, confirming the lack of crystalline order at this stage (Figure S25 in the Supporting Information).

**Figure 10:**
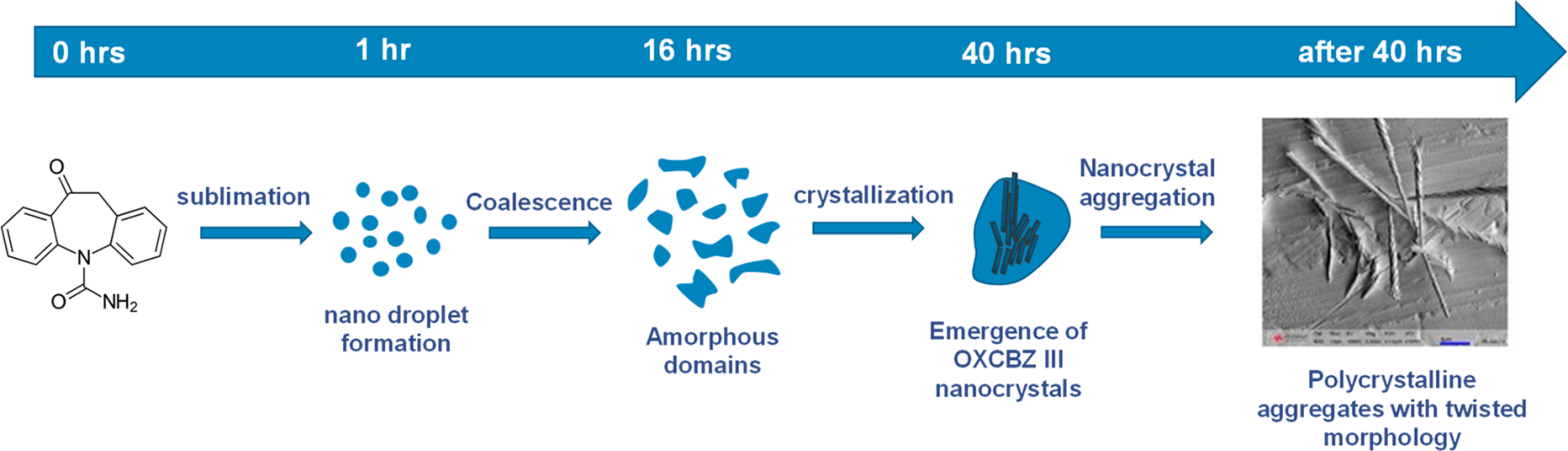
Schematic representation of the proposed crystal growth mechanism observed for OXCBZ III in sublimation experiments.

After 16 h (Figure 8a), the droplets have grown through continued deposition and/or coalescence to produce larger noncrystalline, amorphous domains of average diameter 808 ± 243 nm (*n* = 30 domains). SEM micrographs collected after 40 h of sublimation revealed the eventual emergence from these larger droplets of coalesced condensate of needlelike nanocrystals that are clearly imaged in Figure 8. Untwisted nanocrystals can be observed emerging from these regions (yellow boxes) in addition to the appearance of larger twisted OXCBZ aggregates protruding from the condensate droplet (red boxes). An SEM analysis also showed the presence of both left- and right-handed twisted aggregates (Figure S6 in the Supporting Information). It is notable from Figure 8b that the columnar crystals growing within the amorphous droplets undergo a morphological transformation to aggregate and twist spontaneously along the longest crystal length to form twisted bundles or aggregates. SEM micrographs collected after 40 h of sublimation show elongated, twisted aggregates represented in Figure 8c,d. After 40 h of sublimation the diameter and length of the aggregates were in the ranges of 60–450 nm and 1.5–6 μm, respectively. The plot of crystal length vs pitch for one of the twisted aggregates after 40 h of sublimation is presented in Figure S7 in the Supporting Information. On the basis of these observations, the crystal growth process for twisted OXCBZ III prepared via sublimation is summarized in the schematic in Figure 10.

## 4 Discussion

Following the original report of OXCBZ III, the crystallization screening studies presented here show that form III can be obtained reproducibly by crystallization from solution or via sublimation methods. In addition to the identification of new routes to obtain OXCBZ III, a further assessment of the properties of OXCBZ using pH-controlled intrinsic dissolution shows that OXCBZ III dissolves ca. 2.6 times faster than form I (Section 8.2 in the Supporting Information). Dry samples of OXCBZ form III do not show any evidence of transformation to form I under ambient conditions; therefore, form III presents a potential route to access a useful property enhancement, as OXCBZ is a BCS Class II drug likely to exhibit dissolution-rate-limited oral bioavailability. VT-XRPD and DSC analyses show that OXCBZ III transforms into the thermodynamically stable form I on heating, with DSC data indicating that these two polymorphs are related monotropically. A crystal structure determination of OXCBZ III from XRPD data confirms the close similarity of the determined structure to the CSP-predicted structure a96. The crystal packing contains void channels that are aligned along the crystallographic *c* axis, oriented parallel to the twist axis of the aggregates. Similar channels have been observed in CBZ II (Section S13.2 in the Supporting Information), where solvent inclusion has been demonstrated,^53^ as well as in the isomorphous CYT I structure.

An SEM analysis of crystal formation from the vapor during sublimation experiments shows an unconventional vapor–condensate–solid growth process, a phenomenon that was recently reported for several small organic compounds crystallized by microspacing in-air sublimation.^59^ Strikingly, OXCBZ III produced from vapor and solution recrystallizations often displayed a markedly twisted morphology. On the basis of the observations of particle formation and growth, OXCBZ is considered to be a twisted polycrystalline aggregate. Initial particle formation from the vapor phase shows clear evidence of the formation of straight nanocrystals but with twisting as multiple crystals aggregate as they emerge from the condensate and elongate. The pitch also lengthens as the aggregate diameter and length increase, away from the point of origin. However, no evidence of crystals directly aggregating together in solution has been observed, suggesting that this process occurs during the early onset of crystallization. An increasing number of molecules have been shown to exhibit different types of twists and a range of mechanisms have been proposed to account for this intriguing phenomenon.^60^ Here we consider the mechanism and potential factors that influence twisting in OXCBZ III.

The tendency of columnar crystals to form twisted fibers is dependent on the interplay between the unfavorable elastic energy associated with twisting and the stabilization afforded by the aggregation of crystals^61^ from the reduction in the surface free energy.^62^ The computed morphology of OXCBZ III is a hexagonal needle with a channel running along the length of the needle crystal. Hence, most of the surface of the crystal will have the same free energy but the attractive forces for self-assembly and aggregation will depend upon the medium around the needles: i.e., solvent, air, or the condensate liquid. It is therefore not surprising that differences are observed under different growth conditions: for example, with solution crystallization leading to a lower relative occurrence of twisted particles in comparison with growth from the vapor. In addition, hexagonal needle crystals of the same diameter can readily close pack, making it difficult to distinguish conceptually, let alone experimentally, between a single crystal with domains and a self-assembled aggregate. OXCBZ III appears to be similar to the metastable form of benzamide, which forms twisted aggregates as a result of internal stresses arising from a misfit among thin crystallite intergrowths with different crystallographic orientations.^25^ For the sublimation-grown samples of OXCBZ III, nearby crystallites could be imperfectly aligned by the condensate molecules between them, leading to the emergence of a twisted crystal or agglomerate. Misalignment can arise from different factors such as differences in orientation and diameter or from mismatched unit cell dimensions.

**Figure 11:**
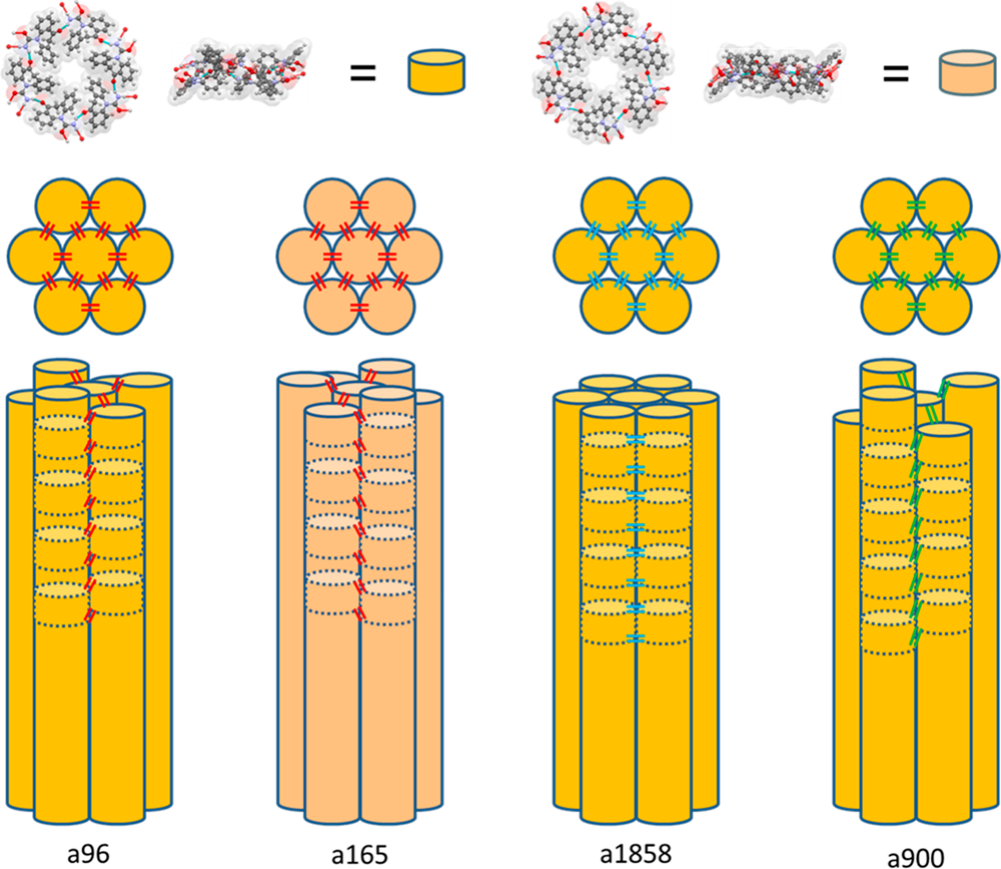
Schematic packing comparison of the four trigonal CSP structures which have similar structures: a96 (*R*3̅), which is identified as OXCBZ form III, and the closely related column packing in a165 (*R****3̅***), a1858 (*P*3̅), and a900 (*R****3̅***). The top row shows top and side views of the two types of hydrogen-bonded units forming the channel void with adjacent channel units connected via *R*_2_^2^(8) amide–amide hydrogen bonds. These are denoted in blue when they form a circuit around the intercolumn space between three adjacent columns, in red if they form a single screw, and in green if they form a double screw. The differences in the interchannel *R*_2_^2^(8) and the steps give rise to a clockwise helical arrangement of hydrogen bonding between the steps for a96 and a900, an anticlockwise helix in a165, and a flat packing in a1858.

One of the possible explanations for a twisted fiber is Eshelby twisting.^63,64^ According to Eshelby’s theory, an axial screw dislocation in a nanocrystal creates an elastic stress field that can be partially relaxed through continuous twisting of the crystal. The Eshelby and other twisting and untwisting mechanisms have been discussed and modeled in molecular crystals such as benzil.^65,66^ While Eshelby twisting could be one of the possible mechanisms of twisting in OXCBZ form III, it is highly unlikely that the twist in a large number of crystals of OXCBZ III would be synchronized since dislocation formation is generally stochastic. The other most common cause of twisting has been identified^29^ as the presence of compositional and structural inhomogeneities in a crystal, producing a lattice mismatch that generates a twist moment at the growth front. The crystal packing of the closely related structures identified on the CSP landscape of OXCBZ (Section S13 in the Supporting Information and Figure 11) suggests that this may be plausible for OXCBZ. Four CSP structures, a165, a1858, a900, and a96 (form III), show similar unit cells and have common void channels parallel to the crystallographic *c* axis (Figure S38 in the Supporting Information), with two variations in the internal *R*_6_^6^(48) hydrogen bonding using the carbonyl groups on the seven-membered ring and one of the NH groups of the amide. The channels are formed with stacked “steps” with different arrangements of the six *R*_2_^2^(8) amide hydrogen bonds that connect each channel to its six neighboring channels (Figure 11).

**Figure 12:**
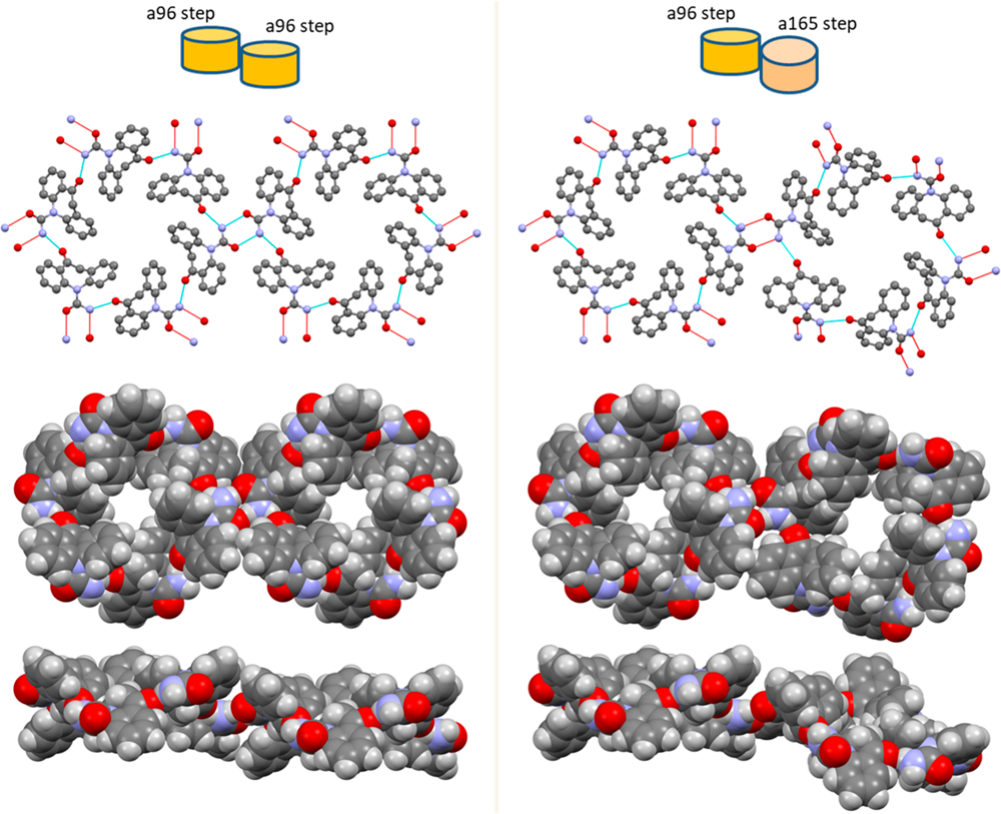
Comparison of the hydrogen bonding between steps of OXCBZ form III (a96) and the possible hydrogen bonding between an a96 step to one of the structures of a165.

The alternative to the form III structure, a96, has a different sense of screw. The channels of a96 form a right-handed screw with the central channel through three *R*_2_^2^(8) hydrogen bonds linking one step in a channel to the immediate step on top of it, whereas in a900 a double screw is formed, in a165 a left-handed screw is formed, and in a1858 a coplanar circuit is formed. This sense of screw dictates how phenyl rings from three OXCBZ molecules in three adjacent channels interdigitate with each other. These three related, hypothetical structures show the potential for growth errors arising from subtly different errors in the molecular packing where there is either a different step structure (as illustrated in Figure 12 for a step of a165) or different attachments of a step to the channel. Such structural inhomogeneities, of either steps or the hydrogen-bonding attachment between channels, have an inherent twist that could initiate the formation of twisted crystals.

Although no direct experimental evidence of structural inhomogeneities was observed in any of the OXCBZ III diffraction data, small amounts of any crystalline phase impurities would be difficult to detect using standard laboratory powder diffractometers at levels below 1–2% by mass.^67,68^ The close similarity in the XRPD patterns of a165, a1858, or a900 and OXCBZ III (Figure S37 in the Supporting Information) increases the difficulty in detecting even significant domains based on these structures.

Chemical impurities are also known to have varied effects on crystals displaying twists, affecting the pitch in twisted crystals by modifying their morphology and packing or causing crystal growth to be suppressed under certain supersaturations.^29^ The incorporation of impurities has been associated with increasing the extent of twist in a number of systems, including aspirin,^26^ and as a possible cause of twisted oxalic acid dihydrate and gypsum crystals.^69^ It is therefore worth highlighting that a chemical impurity, dibenzazepinodione (DBZ; Figure S30 in the Supporting Information) was present in the raw OXCBZ material used in the sublimation experiments (1.8–1.9% w/w) and found to increase in concentration over time in the material reservoir during the experiments (up to 6.6% w/w; Section S9 in the Supporting Information). Small quantities of a chemical impurity (<0.1%) can lead to lattice defects and result in structural mismatch and strain and so, although there is no direct confirmation of DBZ in the OXBZ III product obtained, its effect on twisting in OXCBZ III cannot be ruled out.

This polymorph of OXCBZ highlights the need to develop a more comprehensive understanding of the complex mechanisms and interplay between structure and the thermodynamic and kinetic factors that direct nucleation and growth as well as the formation of a particle microstructure leading to twisting. The increased awareness of the high frequency of occurrence of twisted morphologies across many different classes of materials and the variety and sensitivity of the different mechanisms involved suggest that greater consideration of these aspects should be included within materials development. The challenge remains to ensure that reliable detection occurs during early materials development and that the additional techniques to measure and model these material attributes are routinely incorporated within investigations. For example, standard diffraction methods for phase identification as well as particle sizing or other methods for determining bulk properties will not routinely distinguish standard crystalline morphologies from the diverse range of possible helicoidal, twisted particle shapes. Clearly within the context of the development of pharmaceuticals, for which there are now increasing numbers of documented examples of twisted morphologies, any failure to recognize changes in microstructure as a source of variability has significant implications on the design, manufacturing, and quality of medicines. An improved understanding of the potential effect on stability, processability, or performance would also provide indications of whether this area of crystal growth can be exploited to improve functional material performance.

## 5 Conclusions

OXCBZ III crystallizes from the vapor phase via a nonclassical, multistep nucleation, analogous to some protein^16^ and biomineral^14^ recrystallizations. As crystals grow and aggregate, the twist is reduced as a function of length, consistent with observations in other systems.^25,60^ The unreported crystal structure, key physical properties, and relative stability of OXCBZ III have been determined from a combination of screening, CSP, and characterization techniques. The unexpected finding of twisted OXCBZ III morphologies was also revealed while in pursuit of these important structure–property relationships.

Twisted crystal morphologies were observed under different crystallization conditions and SEM studies of the sublimation-grown OXCBZ III showed that columnar twisted crystals emerge directly from amorphous nanodroplets coalescing on the experimental substrates. The emergent nanocrystals twist spontaneously as they grow, driven by their aggregation into twisted bundles. Some evidence of twisting was also observed for crystals of CBZ form I grown via sublimation (Figure S8b in the Supporting Information), highlighting the relative frequency of twisted morphologies in molecular crystals. In the absence of direct structural evidence for the specific mechanism of formation of twisted crystals in solution-based or sublimation crystallization, close similarities among OXCBZ III and several other CSP-predicted structures are highlighted as showing the potential for growth errors during crystallization, leading to a lattice mismatch and thus twisted crystals. The spontaneous twisting of OXCBZ III crystals limits their radial crystal growth and leads to the formation of bundles of polycrystalline fibers, thus explaining the difficulty in obtaining suitable single crystals for X-ray diffraction measurements and enabling further structural characterization.

## Supporting Information Available

Full details of all the computational modeling and experimental work (PDF)
