# Supplementary material for: Crystal Structure and Twisted Aggregates of Oxcarbazepine Form III
Source: Cryst Growth Des. 2022 May 24;22(7):4146–56. doi: 10.1021/acs.cgd.2c00152 (PMC9337787; doi:10.1021/acs.cgd.2c00152)
Supplement: Supplementary file 1 — cg2c00152_si_001.pdf [file cg2c00152_si_001.pdf]

Electronic Supporting Information

Crystal Structure and Twisted Aggregates of Oxcarbazepine Form III

Hector Polyzois,[1,2,5] Rui Guo,[3] Vijay K. Srirambhatla,[1,2] Monika Warzecha,[1,2] Elke Prasad,[1,2] Alice Turner,[1,2] Gavin W. Halbert,[1,2] Patricia Keating,[4] Sarah L. Price[3] and Alastair J. Florence[1,2]*

[1]*EPSRC Future CMAC Research Hub, University of Strathclyde, Glasgow, G1 1RD, U.K.*

[2]*Strathclyde Institute of Pharmacy & Biomedical Sciences, University of Strathclyde, Glasgow, G4 0RE, U.K.*

[3]*Department of Chemistry, University College London, London, WC1H 0AJ, U.K.*

[4]*Department of Pure and Applied Chemistry, University of Strathclyde, Glasgow, G1 1XL, U.K.*

[5]*National Physical Laboratory, Teddington, Middlesex, TW11 0LW, U.K.*

Table of Contents

I. Experimental ESI....3

S1. Solution-Based Screening Studies....3

S2. Surface Topography of Experimental Substrates....7

S3. Sublimation Studies....10

S3.1 Sublimation Experiments under Atmospheric Conditions....10

S3.2 Sublimation under High Vacuum using QBox....12

S4. Scanning Electron Microscopy (SEM)....12

S4.1 Twisted-Crystal Formation in OXCBZ....17

S4.2 Twisted-Crystal Formation in OXCBZ Analogues....17

S5. Powder X-Ray Diffraction (XRPD)....17

S5.1 Analysis of Samples from Sublimation Experiments....17

S5.2 Analysis of Samples from Solution-Based Crystallization Experiments....20

S5.3 Variable Humidity XRPD (VH-XRPD) Analysis....23

S5.4 Crystal-Structure Determination of OXCBZ Form III....24

S6. Simultaneous Differential Scanning Calorimetry (DSC) & Thermogravimetric Analysis (TGA)....25

S7. Raman Spectroscopy....28

S8. Dissolution Studies....30

S8.1 Determination of Molar Absorption Coefficient (MAC) 30
S8.2 Dissolution Measurements 31
S9. High Performance Liquid Chromatography-Mass Spectrometry (HPLC-MS) . 32
II. Computational ESI 37
S10. Conformation Analysis 37
S11. Crystal Structure Prediction 38
S12. Sensitivity of the Crystal Energy Landscape to Energy Models 41
S12.1 Sensitivity to Polarization by the Environment 41
S12.2 Periodic DFT-D Lattice Energies 42
S12.3 Sensitivity to Temperature 43
S13. Calculated Properties of the CSP candidates for OXCBZ III 45
S13.1 XRPD Patterns, Structures and Similarities 45
S13.2 Channel Properties 47
S13.3 Calculated Growth and BFDH Morphology of OXCBZ III Candidates 48
References …………………………………………………………………………50

I. Experimental ESI

S1. Solution-Based Screening Studies

Solution-based screening of OXCBZ was performed using a Crissy® platform by Zinsser Analytic. A known amount of OXCBZ form I powder sourced from Molekula Ltd., UK (purity 99.4%) was manually added to known amounts of each solvent (either single-component or binary mixture). 30 analytical/reagent grade pure solvents sourced from Sigma-Aldrich UK, VWR Chemicals and Acros Organics were used experimentally. 34 binary solvent mixtures were additionally utilized, several of which comprised Milli-Q® laboratory grade deionized water (Merck Millipore) as one of the solvent components. An IKA® RH digital heating plate and Lab Dancer vortex mixer were used to facilitate solution heating and agitation and ensure dissolution of the starting OXCBZ material. Prior to crystallization, all solutions were manually filtered into clean glass vials using 0.2 μm PTFE syringe filters to eliminate undissolved powder particles. The vials were then transferred to the screening platform and crystallization experiments were carried out at fixed temperatures via fast evaporation under a light stream of nitrogen gas. In the majority of crystallizations, the solutions prepared were agitated at a constant rate using a built-in vortex mixer and complete evaporation typically occurred within 20 - 30 minutes.

The temperature of evaporation for each experiment was controlled using a heating bath circulator by Huber (Peter Huber Kältemaschinenbau AG, Germany) which was filled with silicone oil. In experiments involving the addition of a substrate to investigate the effect on solid-form outcome, a 1 cm x 1 cm tile of commercial Al foil (Caterwrap®, exact purity and thickness unknown, Stephensons) was manually added to each crystallization vial after solution filtration had been carried out. The evaporation temperature, agitation-speed profile and sample holding time used in each experiment were programmed using the WinLissy software and all experiments were run using WinLissy's ZA Runner module. An overview of the experimental workflow implemented using the Crissy® platform is provided in Figure S1 and each experiment was typically performed in triplicate. A summary of all experiments and corresponding results are provided in Tables S1 and S2.

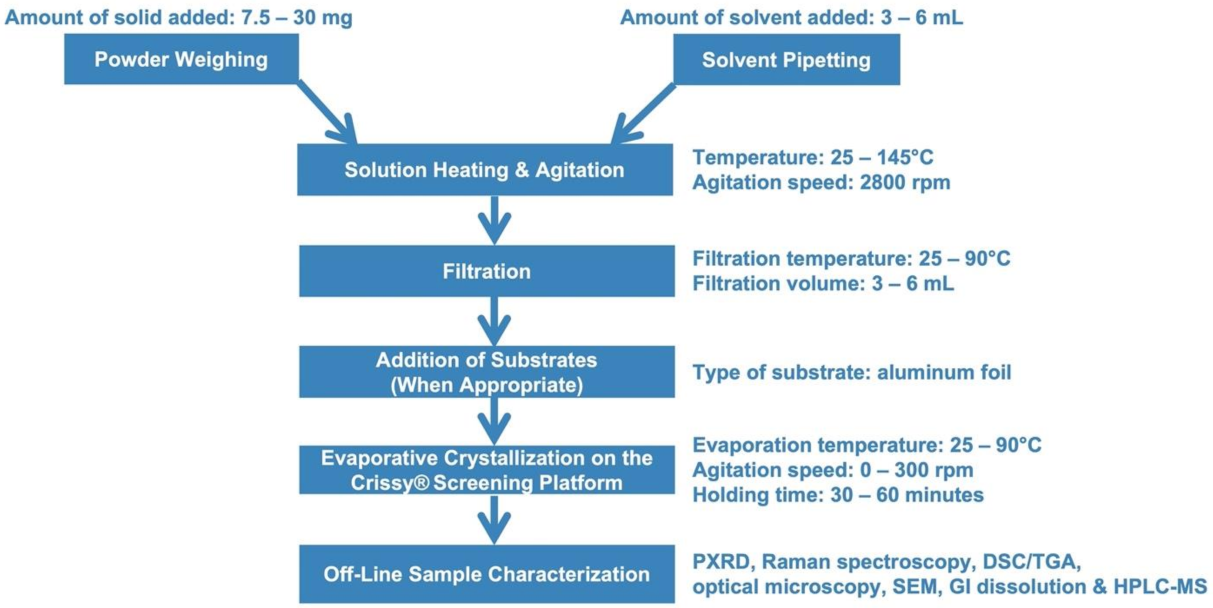

Figure S1. Workflow for evaporative-crystallization experiments performed using the Crissy® screening platform.

Table S1. Summary of results for evaporative-crystallization screening studies of OXCBZ utilizing pure solvents.

Solvent	Evaporation Conditions	Outcome without Substrate	Outcome with Al Substrate
**Acetic Acid**	70°C & 150 rpm	Form I	Form I
**Acetone**	25°C & 150 rpm	Form I	Form I
**Acetonitrile**	45°C & 150 rpm	Mixture of forms I & III	Mixture of forms I & III
**Anisole**	70°C & 150 rpm	Mixture of forms I & III	Mixture of forms I & III
**1-Bromobutane**	50°C & 150 rpm	Mixture of forms I & II	Mixture of forms I & II
**1-Butanol**	50°C & 150 rpm	Form I	Form I
**2-Butanone**	45°C & 150 rpm	Mixture of forms I & III	Mixture of forms I & III
**Chloroform**	25°C & 0 rpm	Mixture of forms I & III	Mixture of forms I & III
**Chloroform**	25°C & 150 rpm	Mixture of form III & unidentified form	Mixture of form I & unidentified form
**1,2-Dichloroethane**	50°C & 150 rpm	Mixture of forms I & III	Mixture of forms I & III
**Dimethylacetamide**	80°C & 150 rpm	Form I	Form I

**Dimethylformamide**	70°C & 150 rpm	Form I	Form I
**Dimethyl sulfoxide**	90°C & 150 rpm	Form I	Form I
**1,4-Dioxane**	50°C & 150 rpm	Form I	Form I
**Ethanol**	45°C & 150 rpm	Mixture of forms I & III	Form I
**2-Ethoxyethanol**	70°C & 150 rpm	Form I	Form I
**Ethyl Acetate**	45°C & 150 rpm	Mixture of forms I & III	Mixture of forms I & III
**Methanol**	25°C & 150 rpm	Form I	Form I
**2-Methoxyethanol**	70°C & 150 rpm	Mixture of forms I & III	Mixture of forms I & III
**Methyl Acetate**	32°C & 150 rpm	Mixture of forms I & III	Mixture of forms I & III
**4-Methyl-2-Pentanone**	70°C & 150 rpm	Mixture of forms I & III	Mixture of forms I & II
**Nitrobenzene**	90°C & 150 rpm	Form I	Form I
**Nitromethane**	50°C & 150 rpm	Mixture of forms I & III	Form I
**1-Pentanol**	70°C & 150 rpm	Mixture of forms I & III	Form I
**1-Propanol**	50°C & 150 rpm	Mixture of forms I & III	Mixture of forms I & III
**2-Propanol**	50°C & 150 rpm	Form I	Form I
**Pyridine**	50°C & 150 rpm	Mixture of forms I & III	Mixture of forms I & III
**Tetrahydrofuran**	25°C & 150 rpm	Mixture of forms I & III	Mixture of forms I & III
**Toluene**	50°C & 150 rpm	Mixture of forms I, II & III	Mixture of forms I & II
**Trichloroethylene**	50°C & 150 rpm	Form I	Mixture of forms I & II
**2,2,2-Trifluoroethanol**	32°C & 150 rpm	Mixture of form III & unidentified form	Mixture of form III & unidentified form
**2,2,2-Trifluoroethanol**	32°C & 0 rpm	Mixture of form III & unidentified form	Mixture of form III & unidentified form
**2,2,2-Trifluoroethanol**	25°C & 150 rpm	Mixture of forms I & III & unidentified form	Mixture of form III & unidentified form

**2,2,2-Trifluoroethanol**	25°C & 0 rpm	Mixture of form III & unidentified form	Mixture of form III & unidentified form

Table S2. Summary of results for evaporative-crystallization screening studies of OXCBZ utilizing binary solvent mixtures. The most phase-pure samples of form III were obtained using the conditions in red.

Solvent (Volume/Volume %)	Evaporation Conditions	Outcome without Substrate	Outcome with Al Substrate
**Acetic Acid/Water (50:50)**	70°C & 300 rpm	Form I	Form I
**Acetone/Water (50:50)**	50°C & 300 rpm	Form I	Form I
**Acetonitrile/Water (50:50)**	50°C & 300 rpm	Form I	Form I
**1-Butanol/Toluene (50:50)**	50°C & 300 rpm	Form I	Form I
**2-Butanol/Toluene (50:50)**	50°C & 300 rpm	Mixture of forms I & III	Mixture of forms I & III
**2-Butanone/Toluene (50:50)**	50°C & 300 rpm	Mixture of forms I, II & III	Mixture of forms I, II & III
**Chloroform/Ethanol (50:50)**	50°C & 300 rpm	Mixture of forms I, II & III	Mixture of forms I, II & III
**Chloroform/Toluene (50:50)**	50°C & 300 rpm	Mixture of forms I, II & III	Mixture of forms I & III
**1,2-Dichloroethane/Toluene (50:50)**	50°C & 300 rpm	Mixture of forms I, II & III	Mixture of forms I, II & III
**Dimethylacetamide/Water (50:50)**	70°C & 300 rpm	Form I	Mixture of forms I & III
**Dimethylformamide/Water (50:50)**	50°C & 300 rpm	Mixture of forms I & III	Mixture of forms I & III
**1,4-Dioxane/Water (50:50)**	50°C & 300 rpm	Form I	Form I
**Ethanol/1-Bromobutane (50:50)**	50°C & 300 rpm	Mixture of forms I & III	Mixture of forms I & III
**Ethanol/Methanol (50:50)**	50°C & 300 rpm	Form I	Mixture of forms I & III
**Ethanol/Nitromethane (50:50)**	50°C & 300 rpm	Form I	Form I
**Ethanol/1-Pentanol (50:50)**	50°C & 300 rpm	Form I	Form I
**Ethanol/Toluene (50:50)**	50°C & 300 rpm	Mixture of forms I & III	Mixture of forms I & III
**Ethanol/Toluene (50:50)**	50°C & 150 rpm	Mixture of forms I & III	Mixture of forms I & III

**Ethanol/Toluene (50:50)**	50°C & 0 rpm	Form III with Form I traces	Mixture of forms I & III
**Ethanol/Toluene (67:33)**	50°C & 0 rpm	Form III with Form I traces	Mixture of forms I & III
**Ethanol/Toluene (33:67)**	50°C & 0 rpm	Mixture of forms I & III	Mixture of forms I & III
**Ethanol/Toluene (20:80)**	50°C & 0 rpm	Mixture of forms I & III	Mixture of forms I & III
**Ethanol/Toluene (80:20)**	50°C & 0 rpm	Mixture of forms I & III	Mixture of forms I & III
**Ethanol/Toluene (10:90)**	50°C & 0 rpm	Mixture of forms I & III	Mixture of forms I & III
**Ethanol/Toluene (90:10)**	50°C & 0 rpm	Mixture of forms I & III	Mixture of forms I & III
**Ethanol/Water (50:50)**	50°C & 300 rpm	Form I	Form I
**2-Ethoxyethanol/Water (50:50)**	70°C & 300 rpm	Form I	Form I
**Ethyl Acetate/Toluene (50:50)**	50°C & 300 rpm	Mixture of forms I & II	Mixture of forms I & II
**Methanol/Water (50:50)**	50°C & 300 rpm	Form I	Form I
**2-Methoxyethanol/Water (50:50)**	70°C & 300 rpm	Form I	Form I
**1-Propanol/Water (50:50)**	50°C & 300 rpm	Form I	Form I
**2-Propanol/Water (50:50)**	50°C & 300 rpm	Form I	Form I
**Pyridine/Water (50:50)**	50°C & 300 rpm	Mixture of forms I & II	Mixture of forms I & II
**Tetrahydrofuran/Water (50:50)**	50°C & 300 rpm	Form I	Form I
**Toluene/4-Methyl-2-Pentanone (50:50)**	50°C & 300 rpm	Mixture of forms I & II	Mixture of forms I & II
**2,2,2-Trifluoroethanol/Water (50:50)**	50°C & 300 rpm	Form I	Form I

S2. Surface Topography of Experimental Substrates for Sublimation Crystallization

The following experimental substrates were utilized in screening studies of OXCBZ and were not modified in any manner prior to experimental use:

- Commercial Al foil, Caterwrap®, exact purity and thickness unknown, Stephensons
- Cu foil, Puratronic®, purity 99.999%, 0.025 mm thick, Alfa Aesar
- Ag foil, purity 99.95%, 0.075 mm thick, Alfa Aesar
- Cu-coated glass
- Ag-coated glass

Before any experiments commenced, all foil substrates were thoroughly rinsed with deionized water (Milli-Q® laboratory grade, Merck Millipore) and 2-propanol (AnalaR NORMAPUR® ACS, Reag. Ph. Eur., purity ≥ 99.7%, VWR Chemicals) and dried under a stream of nitrogen gas in order to remove dust particles and organic contaminants. Cu-coated and Ag-coated glass substrates were fabricated by depositing 200 nm of Cu or Ag (purity 99.99%, Advent Research Materials Ltd.) on 0.17 mm thick glass coverslips sourced from Fisher Scientific UK. Prior to deposition commencing, the glass substrates and metals were cleaned by sonicating in 2-propanol for 30 minutes and drying under a stream of argon gas. The thermal evaporation of the metals was carried out in a MBRAUN MB 200B vacuum evaporator (MBRAUN, Germany) at 10^{-6} mbar pressure with a slow deposition rate of 0.05 nm/s. The topography and average nanoscale roughness (R_a) of substrates for crystallization from the vapor phase were assessed by collecting peak force error and height sensor images using a Dimension FastScan™ atomic force microscopy (AFM) instrument by Bruker. At least 3 images were collected for each substrate under ambient conditions in PeakForce Tapping® mode using Bruker ScanAsyst Air probes with nominal spring constant k = 0.4 N/m and nominal tip radius of 2 nm. The NanoScope *Analysis* software package (v.1.9, Bruker) was utilized to apply first order flattening to all of the height sensor images and calculate substrate R_a values.

AFM images showing the topography of the experimental substrates can be seen in Figure S2 andFigure S3. The average nanoscale roughness of each substrate is indicated by the R_a value. The AFM analysis performed before the deposition studies helped shed light on the topographical features of the experimental templates. The metallic foil substrates were found to have significant roughness with Al foil and Ag foil exhibiting average nanoscale roughness R_a values of 135.7 ± 80.86 nm and 128.7 ± 43.66 nm respectively. Cu foil was found to be the smoothest of the three foil substrates with average R_a value of 19.4 ± 4.76 nm. In contrast with the foil substrates, the metal-coated glass coverslips investigated were found to exhibit significantly smoother surfaces with particularly low nanoscale roughness values. The average R_a values for the Cu-coated and Ag-coated glass substrates measured at 2.46 ± 2.02 nm and 6.50 ± 3.15 nm respectively. The high standard deviation values derived for the Al and Ag foil substrates are indicative of the variability in the roughness of their surfaces across the areas that were scanned. Groove-like features were observed for both Cu foil and Ag foil, whilst the surface of Al foil was highly irregular and uneven. The Ag-coated glass

substrate was found to comprise some spherical features smaller than 6 μm in size which can be attributed to residual dirt. The height sensor images collected show that nanoscale features are clearly protruding from the surface of all experimental substrates.

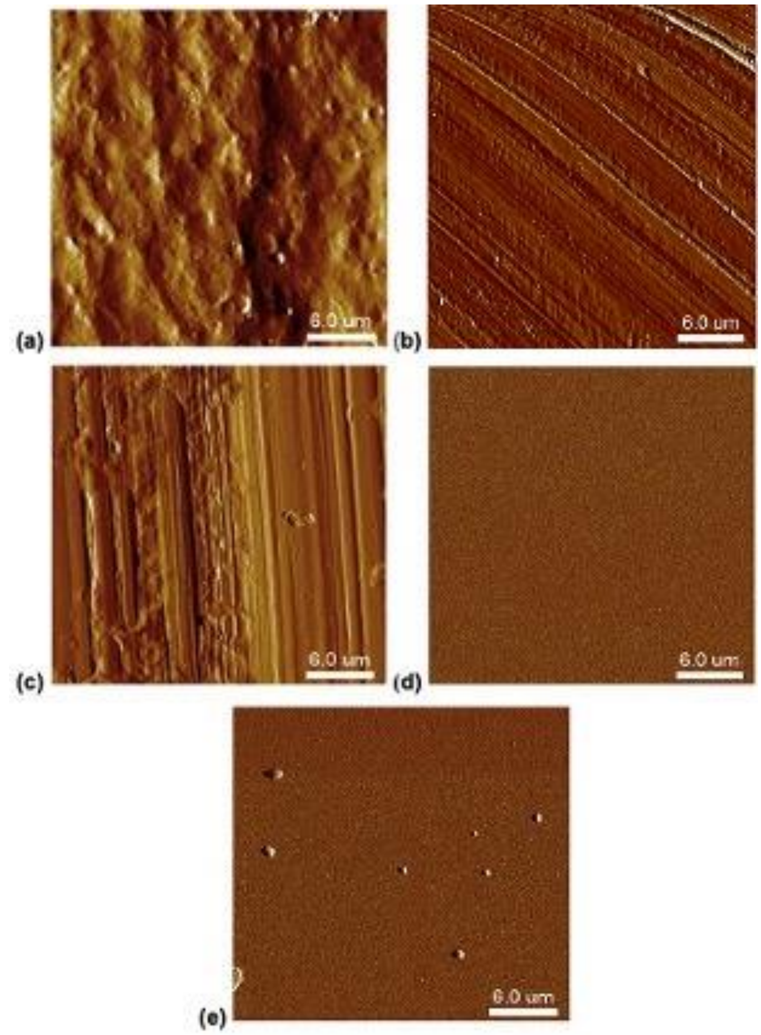

Figure S2. Representative peak force error images of (a) Al foil, R_a = 227 nm (b) Cu foil, R_a = 16.7 nm (c) Ag foil, R_a = 179 nm (d) Cu-coated glass, R_a = 1.04 nm (e) Ag-coated glass, R_a = 2.38 nm.

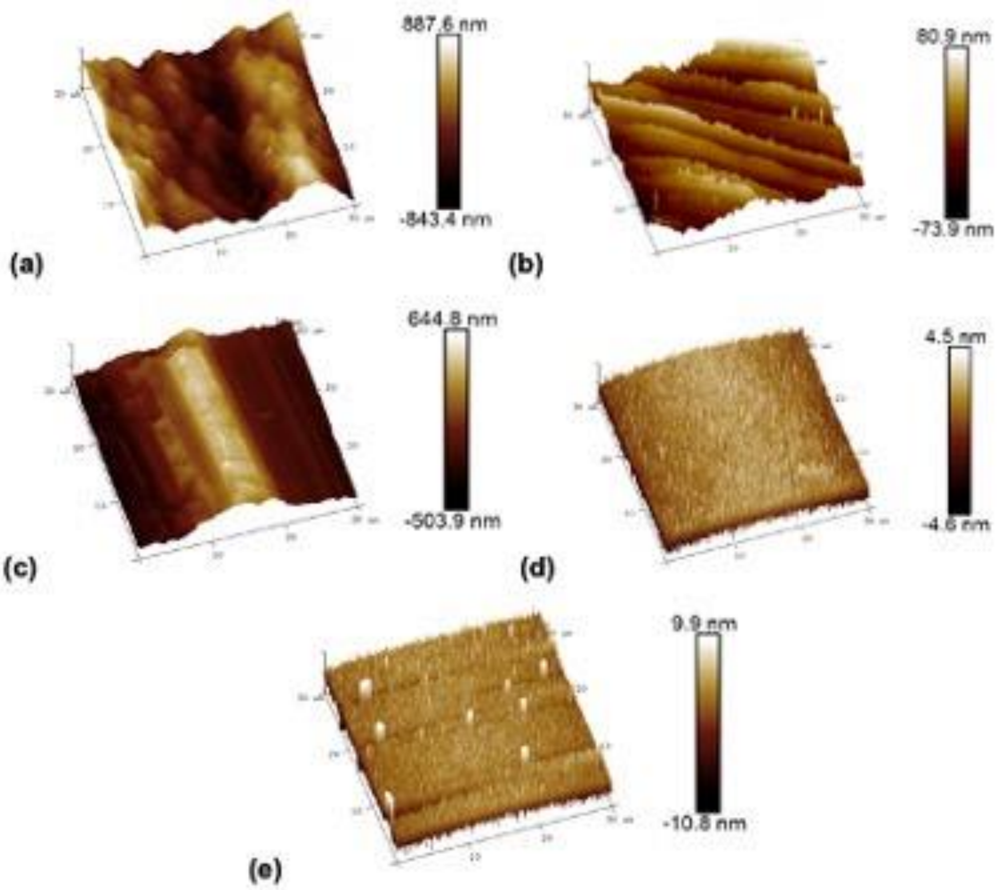

Figure S3. Representative height sensor images of (a) Al foil (b) Cu foil (c) Ag foil (d) Cu-coated glass (e) Ag -coated glass.

S3. Sublimation Studies

S3.1 Sublimation Experiments under Atmospheric Conditions

The sublimation experimental set-up is shown in Figure S4. The starting OXCBZ powder (form I) was sourced from Sigma-Aldrich UK (pharmaceutical secondary standard/certified reference material, purity 99.6%). Metallic substrates (1*1 cm) were attached to the glass slide and exposed to OXCBZ vapors as shown in in Figure S4. The results from the sublimation are tabulated in Table S3.

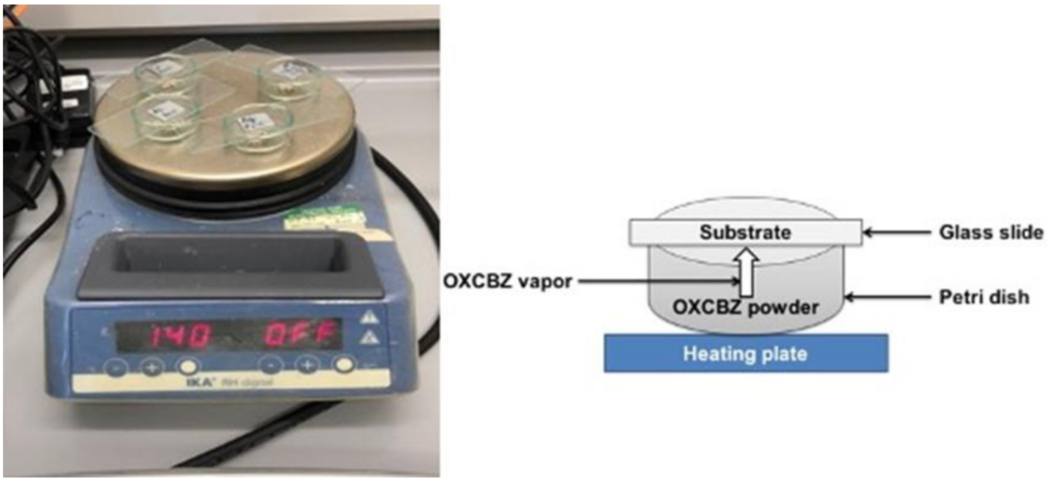

Figure S4. Experimental set-up for sublimation studies of OXCBZ.

Table S3. Overview of sublimation experiments conducted for OXCBZ. The duration of the sublimation process was 48 hours for all experiments.

Substrate	Deposition Temperature (°C)	Polymorphic Outcome
**Al foil**	125	Mixture of form I & impurity peaks
**Al foil**	130	Poorly crystalline material
**Al foil**	135	Mixture of forms I & II
**Al foil**	140	Mixture of forms I & III
**Al foil**	145	Mixture of forms I & III
**Cu foil**	125	Form I
**Cu foil**	130	Poorly crystalline material
**Cu foil**	135	Form I
**Cu foil**	140	Form III
**Cu foil**	145	Form III
**Ag foil**	125	Form I
**Ag foil**	130	Mixture of form I & unidentified form
**Ag foil**	135	Mixture of forms I & III with impurity peaks present
**Ag foil**	140	Mixture of form III & unidentified form
**Ag foil**	145	Mixture of forms I & III with impurity peaks present
**Cu-coated glass**	125	Form I
**Cu-coated glass**	130	Mixture of forms I & III
**Cu-coated glass**	135	Mixture of forms I & III
**Cu-coated glass**	140	Mixture of forms I & III
**Cu-coated glass**	145	Mixture of forms I & III
**Ag-coated glass**	125	Mixture of forms I & III
**Ag-coated glass**	130	Mixture of forms I & III
**Ag-coated glass**	135	Mixture of forms I & III

**Ag-coated glass**	140	Mixture of forms I & III
**Ag-coated glass**	145	Mixture of forms I, II & III

S3.2 Sublimation under High Vacuum using QBox

Experimental outcomes from controlled sublimation studies conducted using a QBox 450[1] system (see section 2.2 in main manuscript for method details) are presented in Table S4.

Table S4. Overview of sublimation studies of OXCBZ conducted using the QBox 450.

Average Deposition Rate (Å/s)	Estimated Film Thickness Derived from QCM (µm)	Average Deposition Temperature (°C)	Distance between Ag Substrate & Starting Material (cm)	Polymorphic Outcome
**2 (slow)**	1.2	140	14	Form I
**2.8 (medium)**	1.2	137	14	Form I
**5.4 (fast)**	1.2	154	14	Form I

S4. Scanning Electron Microscopy (SEM)

SEM micrographs (Figures S5, S6 & S8, S9) were obtained using the methods described in section 2.5 of the main manuscript. The length and pitch of several OXCBZ crystals captured in the SEM micrographs were measured using version 1.51k of the freeware ImageJ.[2] For pitch measurements, the method described by Fang et al.[3] was employed. The correlation between pitch and length in one of the measured crystals is presented in Figure S7.

S4.1 Twisted-Crystal Formation in OXCBZ

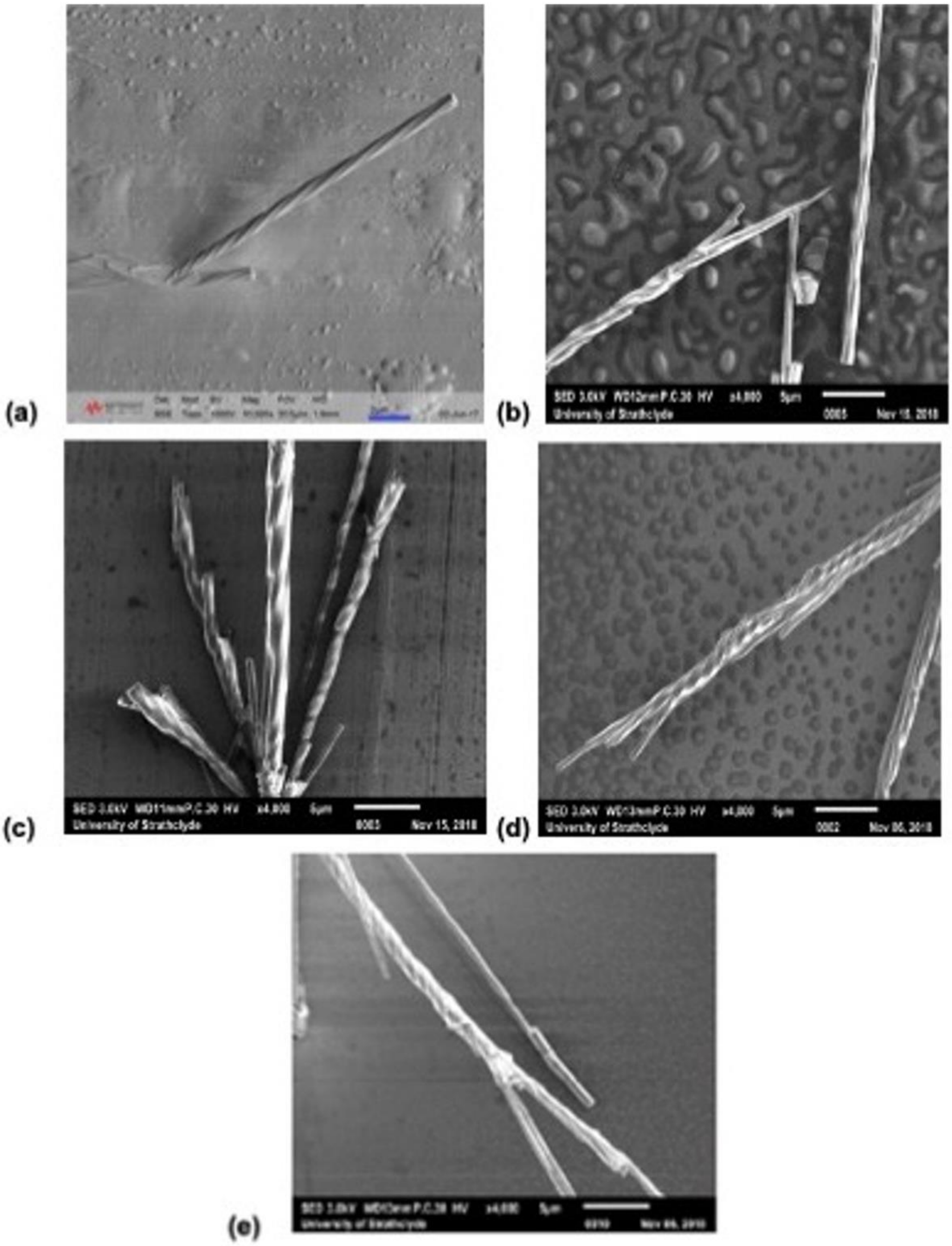

Figure S5. SEM micrographs of twisted OXCBZ III crystals grown via sublimation of OXCBZ onto a) Ag foil, scale bar = 2 μm b) Al foil, scale bar = 5 μm c) Cu foil, scale bar = 5 μm d) Cu-coated glass, scale bar = 5 μm e) Ag-coated glass, scale bar = 5 μm.

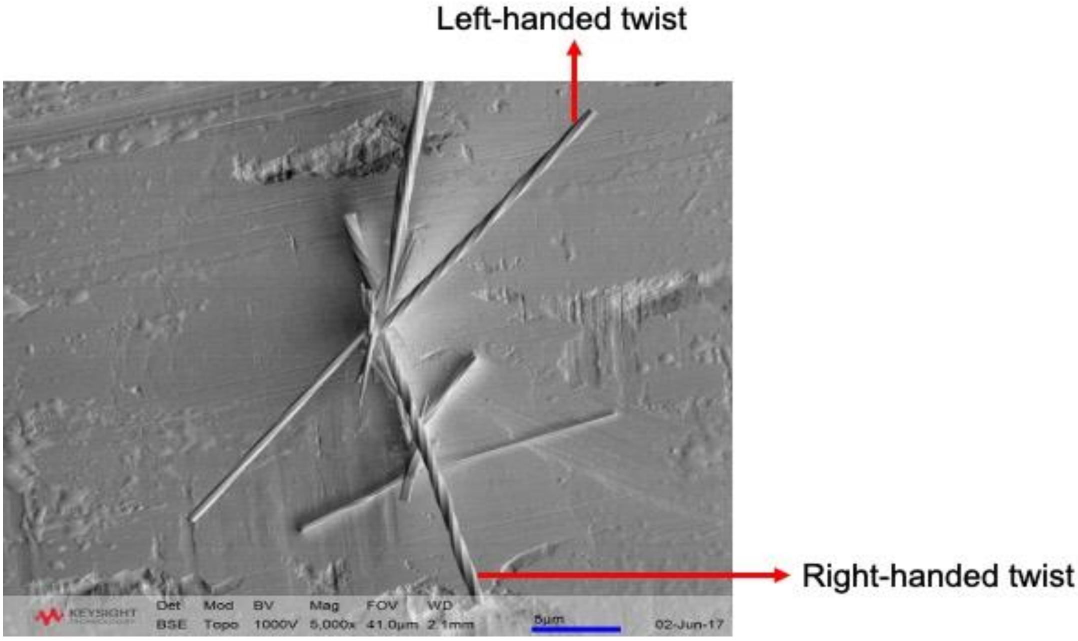

(b)

Figure S6. SEM micrograph showing left- and right-handed twisted OXCBZ III crystals prepared via sublimation onto Ag foil. The scale bar is 5 μm.

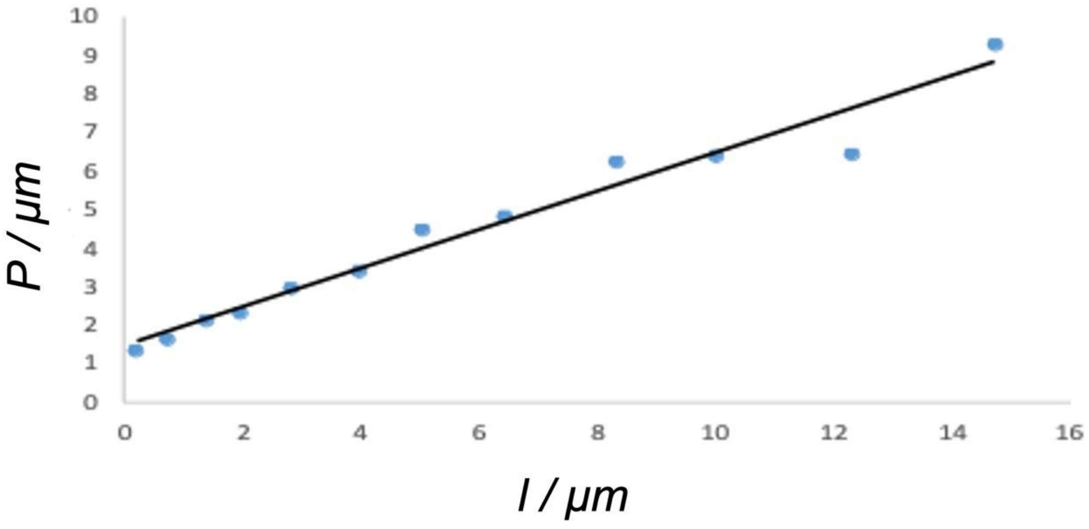

Figure S7. Correlation between pitch (*P*) as a function of length (*l*) in a sublimation-grown, twisted OXCBZ III crystal.

S4.2 Twisted-Crystal Formation in OXCBZ Analogues

To investigate the possible emergence of twisted morphology for the structurally-related analogue molecules carbamazepine (CBZ) and cytenamide (CYT), sublimation onto Ag foil substrates at 140°C and fast-evaporation-from-solution crystallizations were performed. CBZ form III powder sourced from Sigma-Aldrich UK (purity ≥ 98%) and CYT form II powder synthesized using a method described in section 2.1 of the main manuscript were used as starting materials for sample preparation. The SEM micrographs of sublimation-grown CBZ and CYT crystals are shown below in Figures S8 and S9 respectively. Droplets were clearly observed in the CBZ samples prepared by sublimation (Figure S8a), typically following two hours of depositing CBZ onto Ag foil. The sublimation-grown CBZ I are ribbons (Figure S8b), which are fundamentally different to the twisted fibers observed in OXCBZ III. The crystals obtained after 18.5 hours sublimation of CYT are bent (Figure S9) but not twisted crystals. The fast evaporation experiments for CBZ and CYT solutions, performed using the Crissy® platform, did not yield any evidence of twisting (Figure S10).

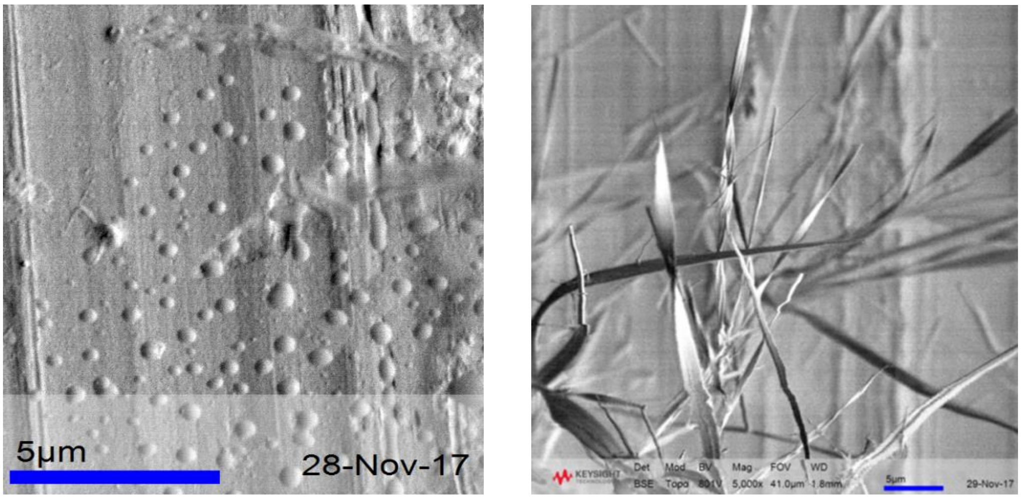

(a) (b)

Figure S8. SEM Micrographs depicting droplets (a) and twisted ribbons of CBZ I crystals grown on Ag foil following 18.5 hours of sublimation (b). The scale bar is 5 µm for both micrographs.

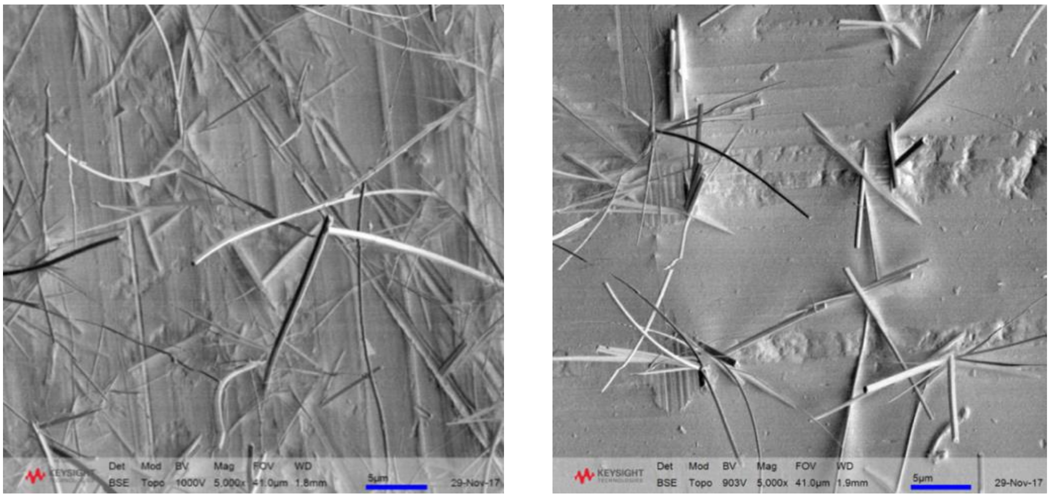

(a) (b)

Figure S9. SEM Micrographs depicting bent and straight CYT crystals grown on Ag foil following 18.5 hours of CYT sublimation. The scale bar is 5 μm for both micrographs.

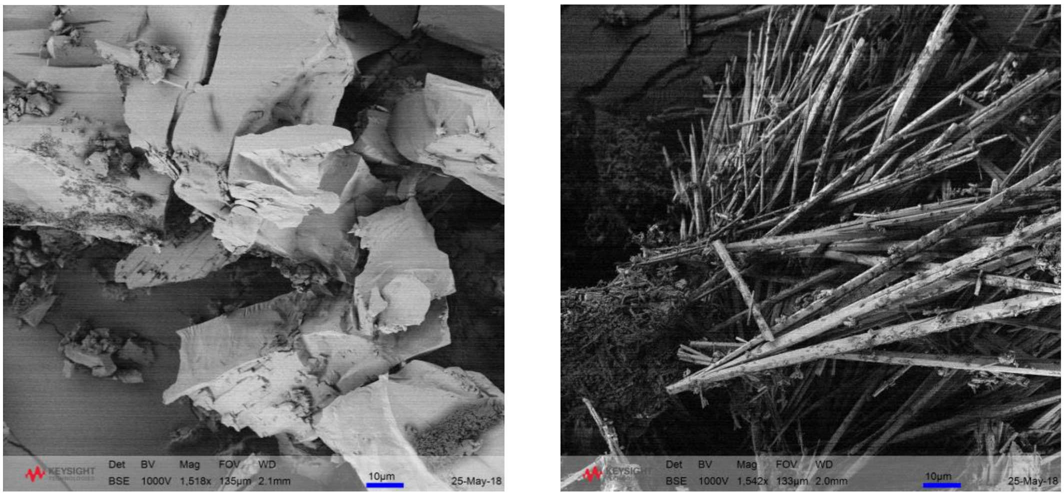

(a) (b)

Figure S10. SEM micrographs depicting CBZ (a) and CYT (b) crystals grown from 50:50 v/v ethanol/toluene mixture via fast evaporation at 50°C. The scale bar is 10 μm for both micrographs.

S5. Powder X-Ray Diffraction (XRPD)

S5.1 Analysis of Samples from Sublimation Experiments

Representative XRPD patterns corresponding to a select number of samples derived from sublimation of OXCBZ, CBZ and CYT onto different experimental substrates are shown in Figure S11-S13. The sublimed materials were scraped onto low-background silicon sample holders and analyzed at room temperature using 1) a Bruker D8 Discover instrument operating at 40 kV and 40 mA in Bragg-Brentano reflection geometry with Cu $K\alpha_{1,2}$ radiation (λ = 1.5416 Å), LynxEye 1D detector, 0.6 mm anti-divergence slit and data being collected in the 2θ range of 3-35° with a 0.01° 2θ step size and count time of 1-30 s/step, 2) a Bruker D2 Phaser instrument operating at 30 kV and 10 mA in Bragg-Brentano reflection geometry with Cu $K\alpha_{1,2}$ radiation (λ = 1.5416 Å), LynxEye 1D detector, 0.6 mm anti-divergence slit. and data being collected in the 2θ range of 3-45° with a 0.01° 2θ step size and count time of 1-10 s/step. Similar acquisition parameters were used to collect the patterns presented in Figures S12 - S14. Pawley fitting[4] of powder data corresponding to poorly-diffracting CBZ material grown on Ag foil (Figure S13) was performed using TOPAS.[5]

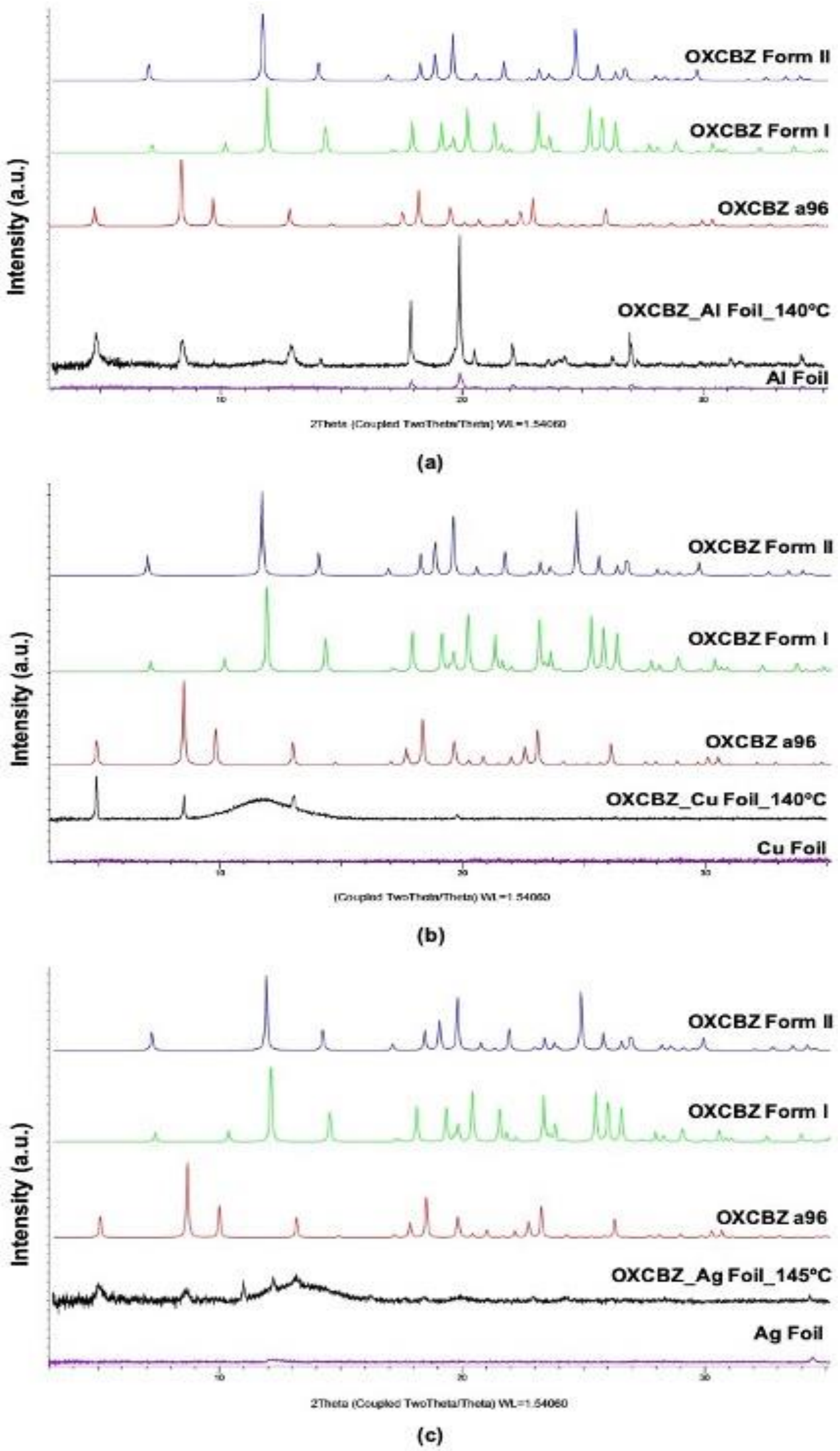

Figure 11. XRPD patterns of OXCBZ material obtained from sublimation onto Al foil at 140°C (a), Cu foil at 140°C (b) and Ag foil at 145°C (c).

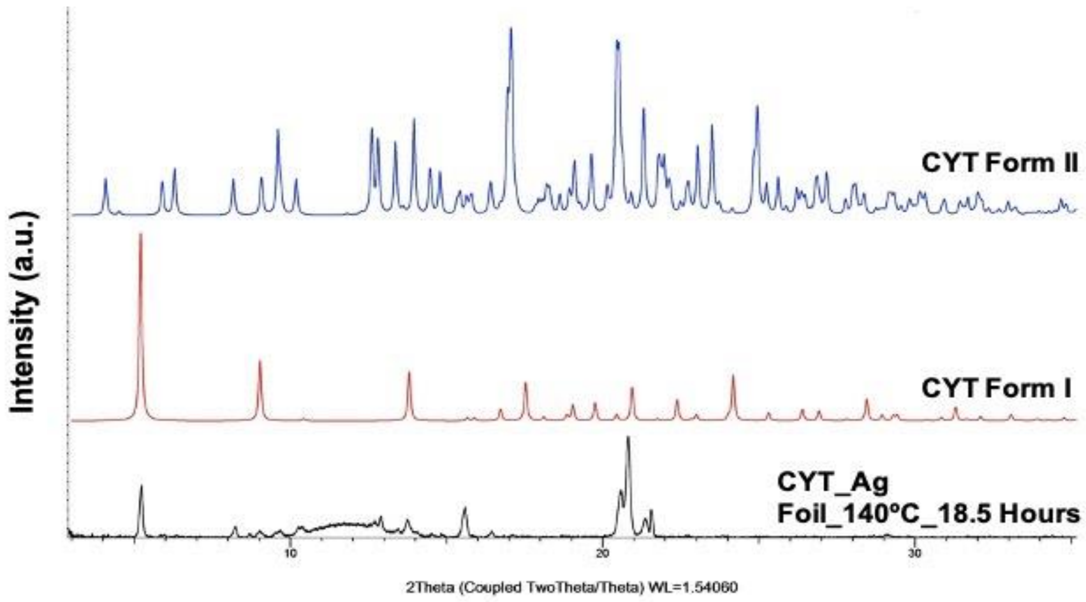

Figure S12. Comparison of XRPD pattern representative of the CYT material obtained via sublimation onto Ag foil over 18.5 hours with simulated patterns of CYT form I (CSD Refcode: SOGLEG) and II (CSD Refcode: SODNOP). The sublimed CYT material is a mixture of forms I & II.

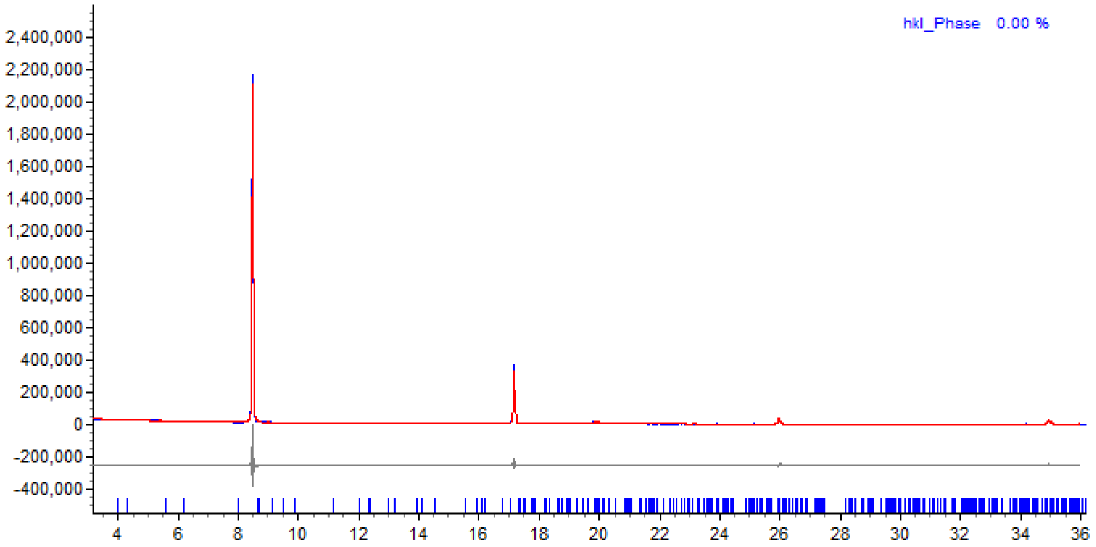

Figure S13. Pawley fit of CBZ material grown on Ag foil by sublimation over 18.5 hours. The blue pattern corresponds to experimentally observed data (y_{obs},) the red pattern represents calculated data (y_{calc}) based on the unit cell parameters of CBZ form I (CSD Refcode: CBMZPN13) and the grey pattern indicates differences between the measured and calculated data [$(y_{obs} - y_{calc})/\sigma(y_{obs})$]. The residual values obtained for the Pawley fit are R_{wp} = 6.98% and R_p = 5.07% with refined cell parameters of a = 5.133 (5) Å, b = 20.642 (17) Å, c = 22.185 (4) Å, α = 84.080 (15)°, β = 88.445

(14)° and γ = 85.243 (8)°. The blue tick marks represent calculated diffraction peaks of form I.

XRPD analysis of starting powders used for sublimation of OXCBZ at different timescales is presented in Figure S14 and indicates the presence of additional, weak crystalline peaks at ≈ 11 and 12.1° 2θ after 48 hours. These peaks were not present before sublimation commenced and can be attributed to the presence of trace amounts of dibenzazepinodione (DBZ), a pharmacopoeial impurity of OXCBZ which has recently been the subject of comprehensive solid-state characterization.[6]

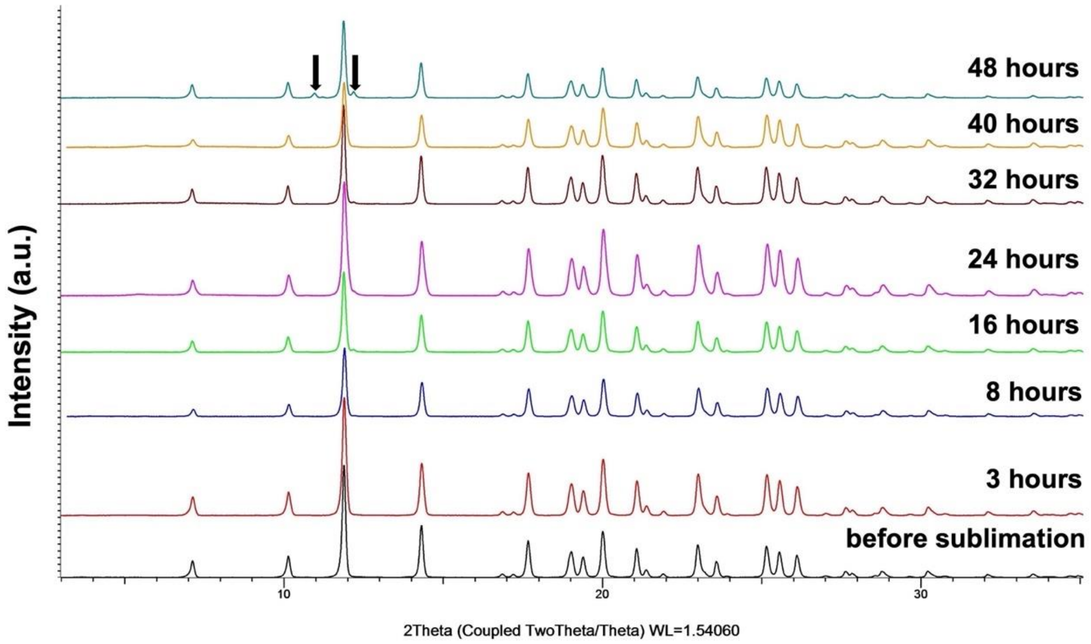

Figure S14. XRPD analysis, corresponding to various timescales, of starting OXCBZ powder specimen (Sigma-Aldrich UK) subjected to sublimation. Black arrows indicate the presence of DBZ impurity peaks at 48 hours.

S5.2 Analysis of Samples from Solution-Based Crystallization Experiments

Solution-grown samples were scraped onto low-background silicon sample holders and analyzed at room temperature using 1) a Bruker D8 Discover instrument operating at 40 kV and 40 mA in Bragg-Brentano reflection geometry with Cu K$\alpha_{1,2}$ radiation (λ = 1.5416 Å), LynxEye 1D detector, 0.6 mm anti-divergence slit and data being collected in the 2θ range of 3-45° with a 0.01 or 0.017° 2θ step size and count time of 1-10 s/step, 2) a Bruker D2 Phaser instrument operating at 30 kV and 10 mA in Bragg-Brentano reflection geometry with Cu K$\alpha_{1,2}$ radiation (λ = 1.5416 Å), LynxEye 1D detector, 0.6

mm anti-divergence slit. and data being collected in the *2θ* range of 3-45° with a 0.01° *2θ* step size and count time of 0.1-10 s/step, 3) a Bruker D8 Advance II instrument operating at 40 kV and 50 mA in Debye-Scherrer transmission geometry with Cu $K\alpha_{1,2}$ radiation (λ = 1.5416 Å), Vantec 1D detector, 1 mm anti-divergence slit, 28 position sample plate supported on Kapton® film and data being collected in the *2θ* range of 4-35° with a 0.017° *2θ* step size and count time of 1-8 s/step. Pawley fitting of select solution-grown OXCBZ samples (Figures S15 – S18) was performed using TOPAS.

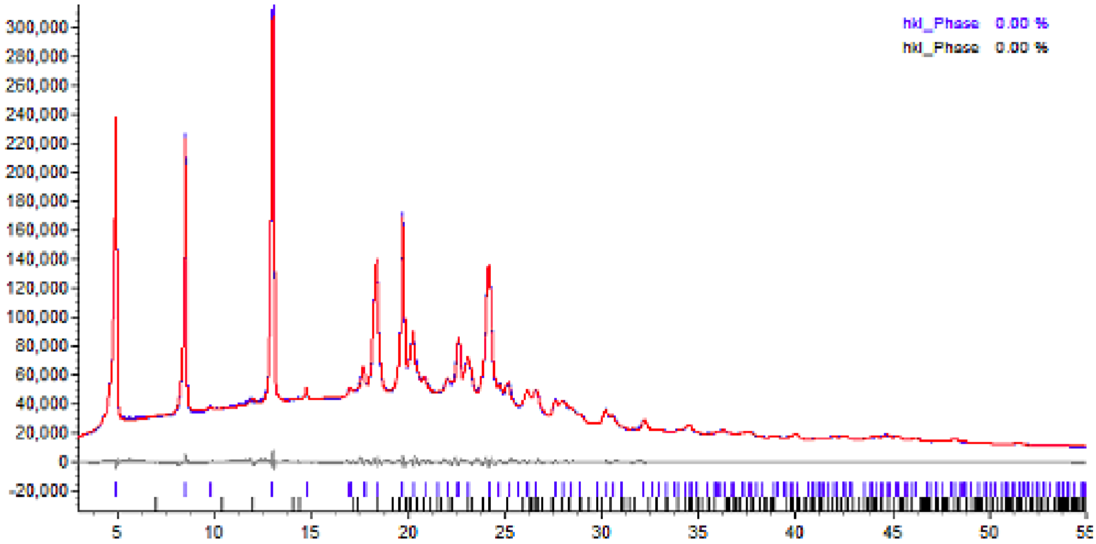

Figure S15. Two-phase Pawley fit of OXCBZ material obtained via fast evaporation from 50:50 (v/v) ethanol/toluene. Data fitting was performed using the unit cell parameters of CSP candidate structure a96 and OXCBZ form I (CSD Refcode: CANDUR01). The residual values are R_{wp} = 1.92% and R_p = 1.46% with refined cell parameters of *a* = *b* = 35.988 (15) Å, *c* = 5.297 (5) Å for form III and *a* = 5.109 (3) Å, *b* = 9.066 (10), *c* = 25.29 (6), β = 93.67 (5)° for form I. The blue and black tick marks represent calculated diffraction peaks of structure a96 and OXCBZ I respectively.

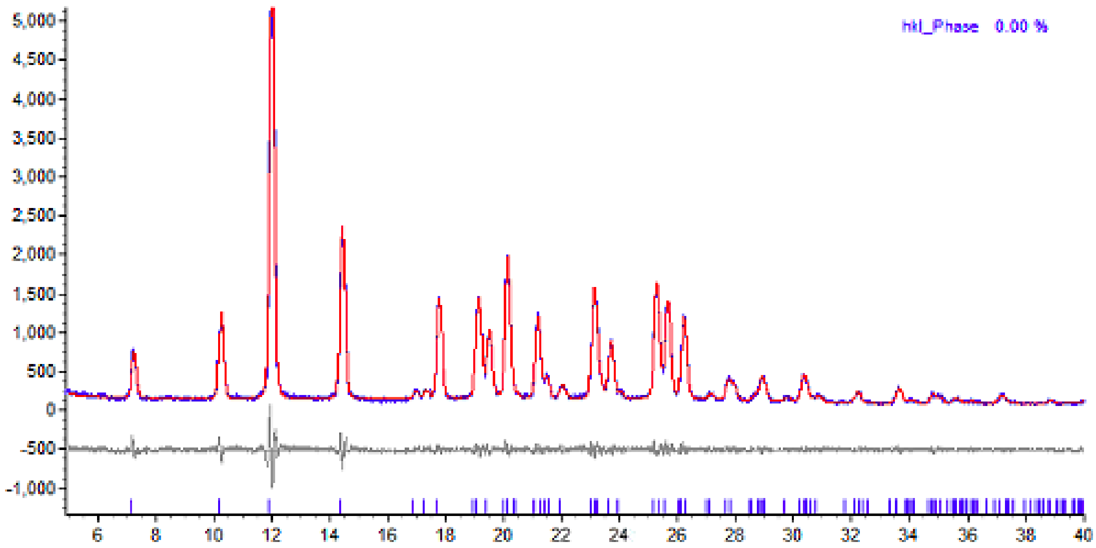

Figure S16. Pawley fit of OXCBZ material prepared by fast evaporation from 2,2,2-trifluoroethanol following desolvation at 65°C in a drying oven. Data fitting was performed using the unit cell parameters of OXCBZ form I (CANDUR01). The residual values are R_{wp} = 8.24% and R_p = 6.32% with refined cell parameters of a = 5.284 (14) Å, b = 9.310 (3) Å, c = 24.867 (6) Å and β = 95.635 (4)°.

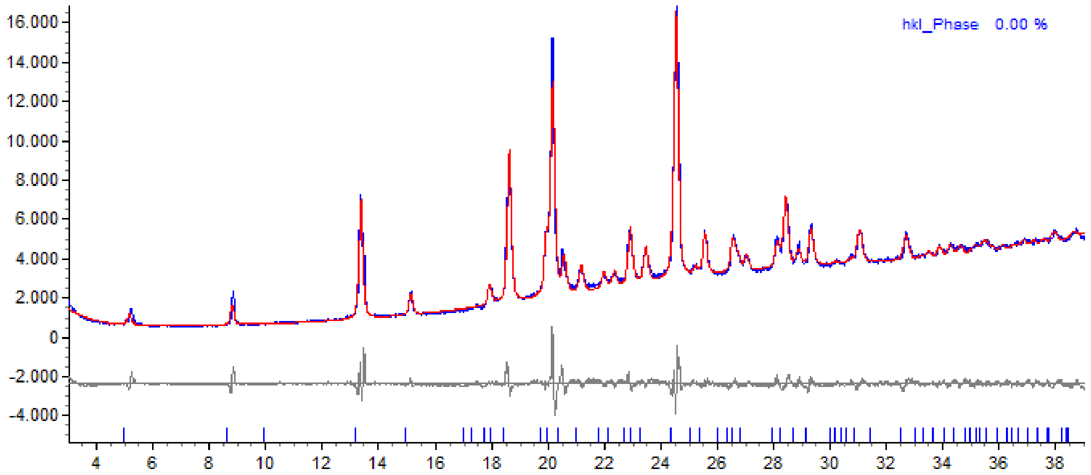

Figure S17. Pawley fit of CBZ material grown from 50:50 v/v ethanol/toluene mixture using fast evaporation. Data fitting was performed using the unit cell parameters of CBZ form II **(**CSD Refcode: CBMZPN03**)**. The residual values are R_{wp} = 5.55% and R_p = 3.57% with refined cell parameters are a = b = 35.541 (16) Å and c = 5.284 (2) Å.

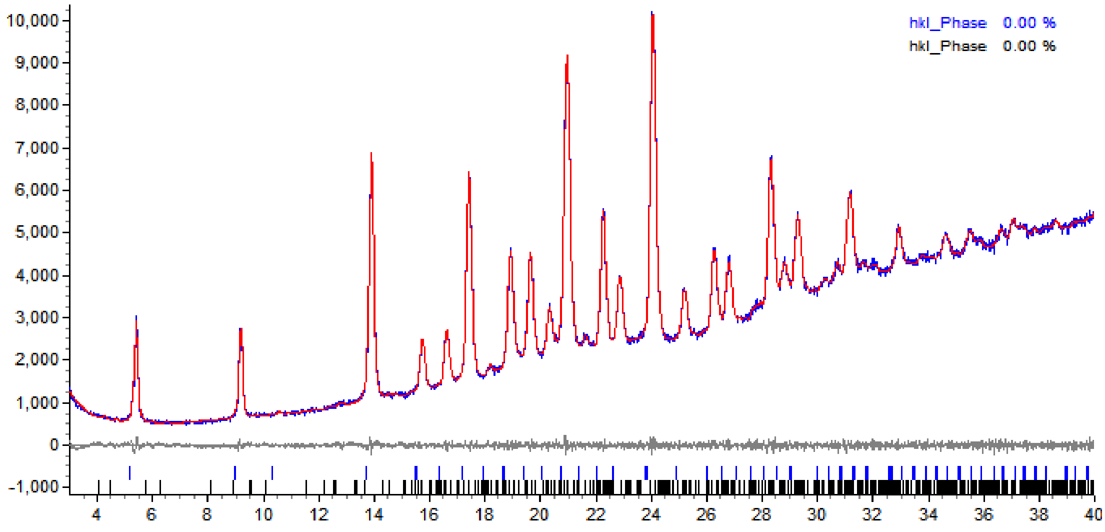

Figure S18. Pawley fit of CYT material grown from 50:50 v/v ethanol/toluene mixture using fast evaporation. Data fitting was performed using the unit cell parameters of CYT form I (SOGLEG) and II (SODNOP). The residual values are R_{wp} = 2.08% and R_p = 1.49% with refined cell parameters of a = b = 34.21 (5) Å, c = 5.825 (15) Å for form I and a = 5.89 (3) Å, b = 19.98 (3) Å, c = 21.93 (5) Å, α = 85.05 (12)°, β = 85.1 (4)°, γ = 85.5 (3)° for form II. The blue and black tick marks represent calculated diffraction peaks of CYT I and II respectively.

S5.3 Variable Humidity XRPD (VH-XRPD) Analysis

VH-XRPD studies of OXCBZ III materials were conducted using the Bruker D8 Discover diffractometer, equipped with Anton Paar CHC plus$^+$ Cryo and Humidity chamber and modular humidity generator MHG (ProUmid, Germany). Data were collected from 5 to 95% relative humidity (RH) in increments of 5% RH using a scan range of 3-40° 2θ, step size of 0.017° 2θ and count time of 1 s/step. All measurements were obtained at a temperature of 20°C and samples were held at each of the humidity profiles investigated for 15 minutes prior to data collection commencing.

The results obtained for the variable humidity study (Figure S19) performed for the OXCBZ III material revealed no significant differences between the diffraction patterns collected at different relative humidity profiles, suggesting that form III remains stable and does not convert to the thermodynamically stable form I or the metastable form II when subjected to high-moisture uptake.

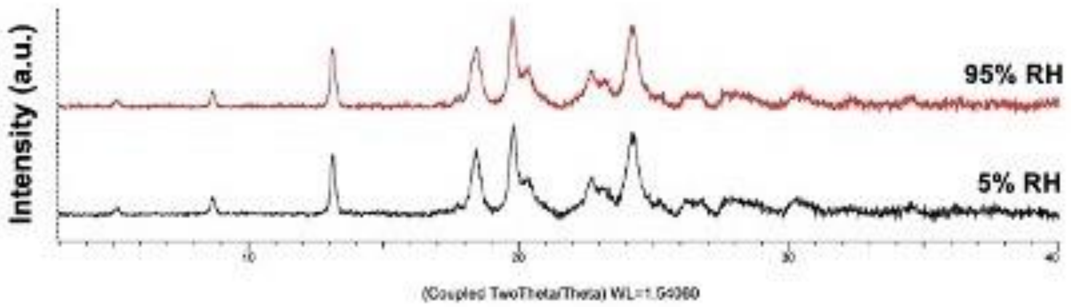

Figure S19. VH-XRPD analysis of OXCBZ form III material obtained from 67:33 (v/v) ethanol/toluene mixture by fast evaporation.

S5.4 Crystal-Structure Determination of OXCBZ Form III

The crystal structure of OXCBZ III was solved by real-space approach[7] using the simulated annealing algorithm as implemented within DASH.[8] Starting lattice parameters obtained from a Pawley refinement of the a96 cell to the data, the space group and molecular geometry from the CSP-predicted structure a96 were all used as input. The positions and spatial orientations of the molecules in the unit cell as well as the rotations around single bonds were varied by using the Mogul distribution bias.[9] Several trial structure solutions from different DASH runs exhibited very similar molecular geometry. A representative structure from trial solutions was refined using the Rietveld method[10,11] in TOPAS. To ensure sensible molecular geometries, standard restraints were applied to bond length, bond angles, and for planar groups in the final Rietveld cycle. A comparison of the Rietveld-refined OXCBZ III unit cell parameters with CSP structure a96 is shown in (Table S5).

Table S5. Comparison of the unit cell parameters of OXCBZ III (experimental) Vs CSP predicted structure a96.

	**OXCBZ III (Experimental)**	**OXCBZ CSP a96**
**Chemical formula**	$C_{15}H_{12}N_2O_2$	$C_{15}H_{12}N_2O_2$
**Crystal system**	Hexagonal	Hexagonal
a **(Å)**	35.974 (2)	36.0565
b **(Å)**	35.974 (2)	36.0565
c **(Å)**	5.2917 (5)	5.3017
**α (°)**	90	90
**β (°)**	90	90
**γ (°)**	120	120
**Space group**	$R\bar{3}$	$R\bar{3}$
**Volume (Å³)**	5930.66	5969.16
Z	18	18
**Radiation type**	Monochromated Cu $K\alpha_1$ (λ = 1.54060 Å)	N/A

S6. Simultaneous Differential Scanning Calorimetry (DSC) & Thermogravimetric Analysis (TGA)

Simultaneous DSC/TGA analysis (see section 2.3 in main manuscript for method details) performed on polycrystalline material of OXCBZ III and OXCBZ I is shown in Figure S20. The DSC thermogram of OXCBZ I displays a single endothermic peak at 232.1°C corresponding to the melting of the compound. In comparison to OXCBZ I, the DSC curve of OXCBZ III exhibits two transitions upon heating with peak temperatures at 150.7°C and 230.7°C, followed by decomposition of the material. Whilst the endothermic transition at 230.7°C is consistent with the melting of OXCBZ I, as shown in Figure S20, the first exothermic transition between 135°C – 170°C is attributed to a solid-solid phase transformation from OXCBZ III to OXCBZ I, further confirmed by variable temperature XRPD analysis and indicative of a monotropic[12,13]

relationship between forms I and III. The TGA curve of OXCBZ III indicates a weight loss event between 135 – 170°C, coinciding with the exothermic transition observed in DSC and suggesting that the OXCBZ III→I transition is accompanied by non-stoichiometric residual solvent loss in the material which corresponds to ≈ 0.045 moles of toluene or ≈ 0.089 moles of ethanol per 1 mol of OXCBZ.

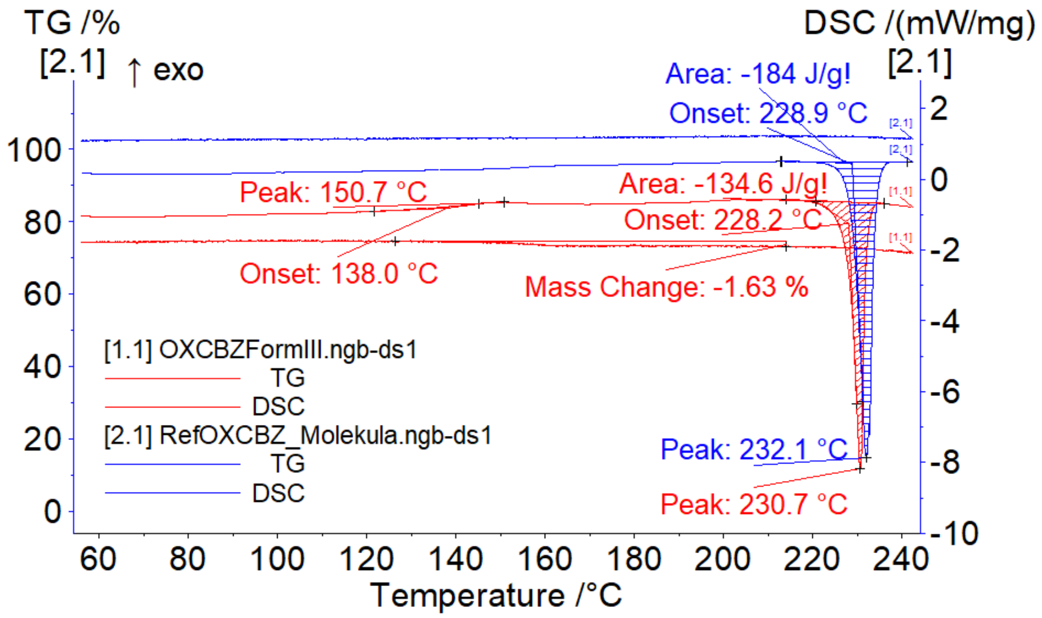

Figure S20. DSC/TGA analysis of powder OXCBZ III and I materials. Arranged top to bottom, the top two blue traces are TGA and DSC curves of OXCBZ I while the bottom two red curves represent the DSC and TGA traces of OXCBZ III respectively.

Figure S21 is DSC/TGA curve of OXCBZ material obtained by fast evaporation from 2,2,2-trifluoroethanol (TFE). The 11% weight loss in the TGA corresponds to around 0.38 moles of TFE. The powder pattern (Figure S16) of the material following attempted desolvation is consistent with OXCBZ I. This indicates that the OXCBZ material obtained by recrystallization from TFE transforms to OXCBZ form I upon desolvation.

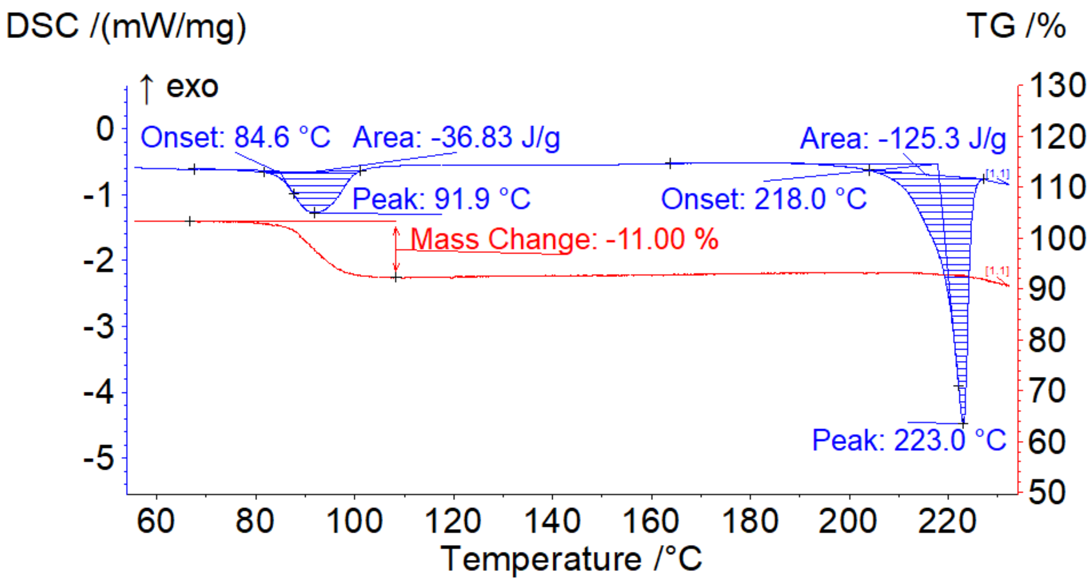

Figure S21. Representative DSC/TGA curves of OXCBZ material prepared by fast evaporation from 2,2,2-trifluoroethanol.

DSC analysis was further performed to cross-correlate the presence of impurity peaks observed in XRPD analysis (Figure S14). At sublimation times > 16 hours, the melting point of OXCBZ starting powder used to grow the twisted crystals was found to decrease by at least 1°C when compared to the reference material from Sigma-Aldrich UK (Figure S22). At 48 hours of sublimation, the decrease in melting point was ≈ 3.7°C compared to the reference, indicating that the purity of the sample had changed considerably. These observations align well with the general expectation that impurity presence in a sample will result in its melting point decreasing.[14]

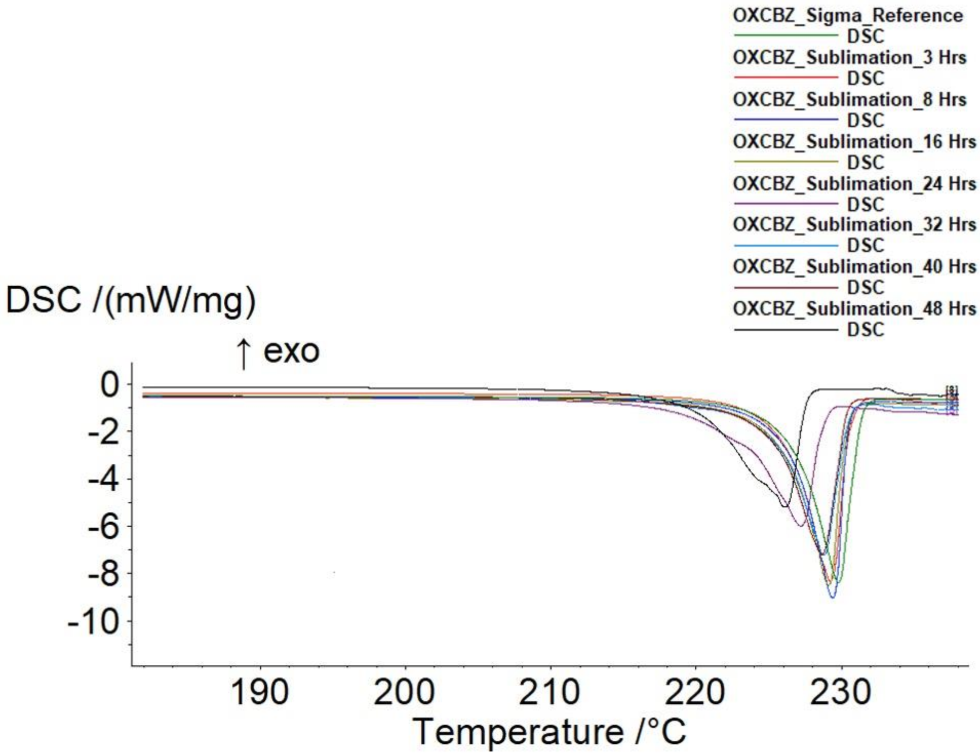

Figure S22. DSC analysis, at various timescales, of starting OXCBZ powder samples used in sublimation experiments and overlay with data for the reference OXCBZ material sourced from Sigma-Aldrich UK.

S7. Raman Spectroscopy

Raman spectra of OXCBZ samples (Figures S23 – S25) were obtained under ambient conditions using an XploRA™ PLUS Raman microscope by HORIBA Scientific, calibrated with a silicon standard and equipped with a motorized stage, CCD detector, 50x objective lens and either 532 or 785 nm excitation laser source with 1200 grooves/mm grating. All samples were measured on a glass microscope slide and spectra were acquired within the 10 - 4000 cm^{-1} shift region using 2 accumulations, exposure time of 10 - 60 seconds and resolution > 1.4 cm^{-1} FWHM. A variety of slit, hole, and filter settings were utilized in order to minimize fluorescence effects and optimize the quality of the spectra during data collection. The LabSpec 6 Spectroscopy Suite (HORIBA Scientific) was employed for performing background subtraction of the obtained spectra where applicable.

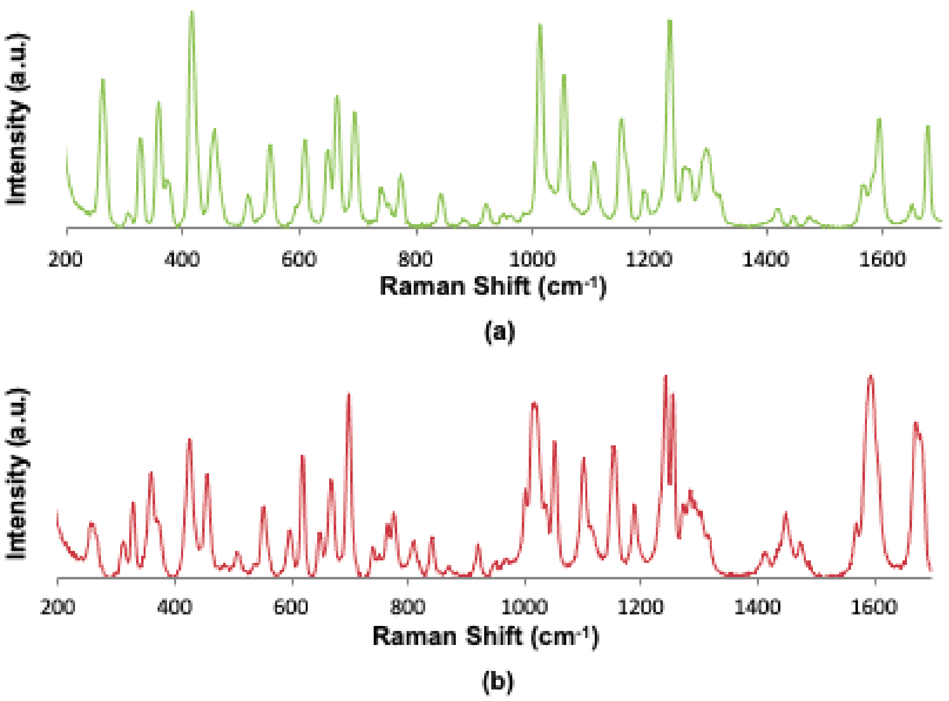

Figure S23. Raman spectra of pure OXCBZ form I sourced from Molekula (a) and OXCBZ form III obtained from 50:50 (v/v) ethanol/toluene mixture by fast evaporation (b).

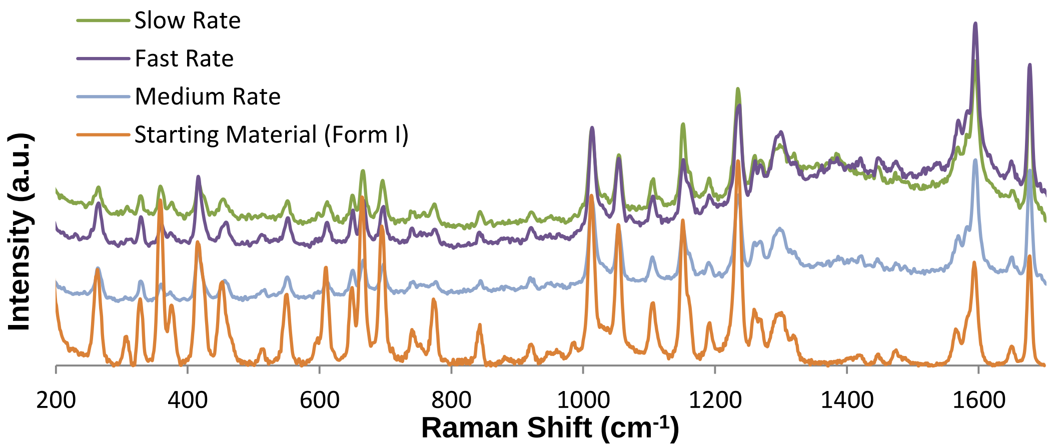

Figure S24. Overlay of Raman spectra of OXCBZ films grown on Ag foil substrates using different deposition rates with a reference spectrum for form I OXCBZ powder sourced from Sigma-Aldrich UK.

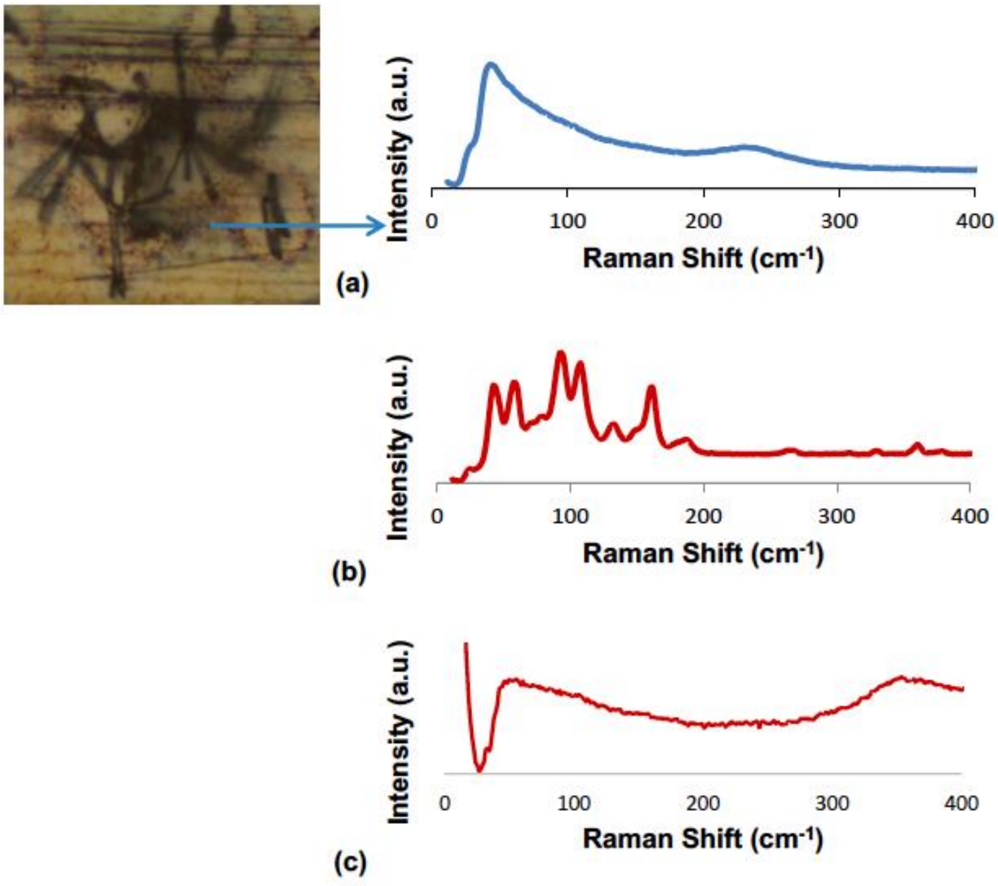

Figure S25. Representative low-frequency Raman spectra of (a) OXCBZ droplet on Ag foil, (b) OXCBZ form I sourced from Sigma-Aldrich UK and (c) amorphous OXCBZ generated via fast evaporation from a saturated solution of 2-methoxyethanol at 70°C and 150 rpm using the Crissy® platform.

S8. Dissolution Studies

S8.1 Determination of Molar Absorption Coefficient (MAC)

A starting solution was obtained by dissolving OXCBZ form I sourced from Molekula in DMSO to achieve an initial solution concentration of 50 mM and latterly a 100 mM concentration. A Sirius Inform instrument (Sirius PAT2000i from Sirius Analytical, now Pion Inc., East Sussex) was utilized to obtain the MAC value by performing a p*K*a assay. 25 µL of solution were added to a vial and the Inform p*K*a assay was carried out using the Inform Control software as follows: 1) Initially, a blank reading was taken titrating from pH 2 to pH 12 in 40 mL of sodium chloride solution using hydrochloric acid and sodium hydroxide. 2) 33 mL of potassium chloride and 2 mL of acetate phosphate buffer were added in an automated fashion. 3) A total of 3 titrations were performed by the instrument going initially from low pH to high pH, then high pH to

low pH and then ultimately low pH to high pH using hydrochloric acid and sodium hydroxide. Data were analyzed using the Inform Refine software, importing the blank aqueous reading to give a mean MAC value based on the molecular weight of OXCBZ which could be imported into the dissolution data set.

S8.2 Dissolution Measurements

Powder samples (17 - 21 mg) of OXCBZ I sourced from Molekula and OXCBZ III prepared from 67:33 v/v ethanol/toluene were compressed into tablets with a diameter of 6 mm to obtain a uniform surface area, using a manual hydraulic tablet press. The duration of compression for each tablet was 5 minutes and a compression weight of 0.5 tonnes was employed. The tablets were added to Inform vials manually in advance of the measurements. Samples were exposed to a pH range from 2 to 7.4 to simulate the effect of gastrointestinal (GI) transit as follows: 1) 36 mL of potassium chloride and 4 mL of acetate phosphate buffer were added in an automated fashion. 2) The pH was adjusted in an automated fashion to pH 2 using hydrochloric acid. 3) The pH was maintained for approximately 20 minutes before gradually increasing to pH 5.5 by addition of sodium hydroxide. 4) The pH was maintained for approximately 20 minutes before gradually increasing to pH 6.5 by addition of sodium hydroxide. 5) The pH was maintained for approximately 20 minutes before gradually increasing to pH 7.4 by addition of sodium hydroxide. All measurements for OXCBZ I and III were carried out in triplicate using a temperature of 25°C and stirring speed of 100 rpm. Data analysis was performed using Inform Refine, importing the blank aqueous reading and the mean MAC value based on the molecular weight of OXCBZ. All results were plotted and compared using Microsoft Excel.

Data from GI dissolution studies comprising OXCBZ I and OXCBZ III compacts are presented in Figure S26. Across the pH range explored, the extrapolated dissolution rate was found to be higher for samples of form III when compared to pure form I material sourced from Molekula. Over the first ≈ 30 minutes of dissolution, the dissolution of compacts that included form III was found to be ≈ 2.6 times greater than those containing form I. The use of compacts with identical diameters in these studies ensured that differences in the particle size of forms I and III would not affect the dissolution outcome. OXCBZ III dissolving more readily than OXCBZ I is in line with past observations of metastable pharmaceutical polymorphs exhibiting improved

dissolution compared to their more stable counterparts.[15] OXCBZ is a BCS Class II drug likely to exhibit dissolution rate-limited bioavailability[16] and, whilst the oral bioavailability profile of OXCBZ III was not investigated herein, it is anticipated that the superior dissolution of form III relative to form I will result in more favourable bioavailability properties and, potentially, more effective pharmaceutical formulations of OXCBZ.[15]

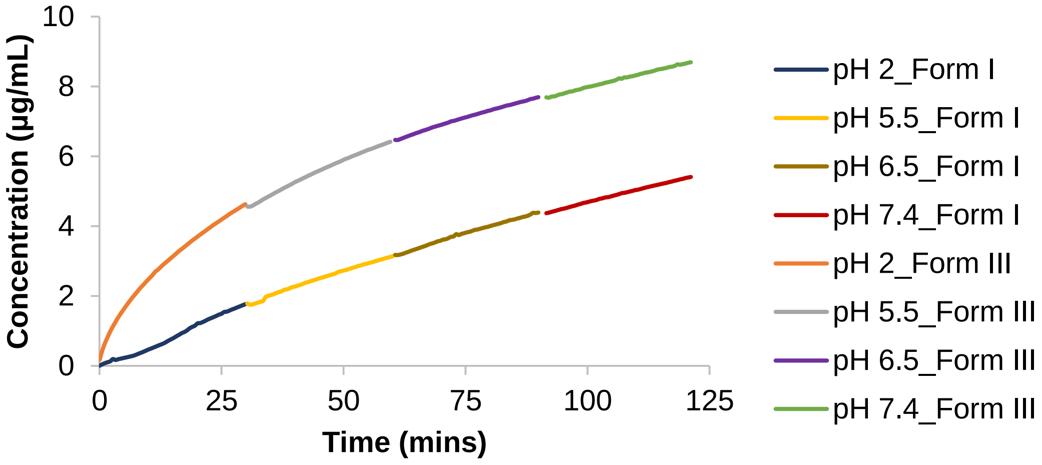

Figure S26. pH dependent dissolution profiles of OXCBZ form I and form III. Dissolution data were collected in the pH range of 2 - 7.4 and the profiles depicted represent average measurements corresponding to 3 samples.

S9. High Performance Liquid Chromatography-Mass Spectrometry (HPLC-MS)

The OXCBZ starting powders undergoing sublimation for 16, 24, and 48 hours were additionally analyzed with HPLC-MS analysis. Prior to analysis commencing, blank samples were run to establish reference data for subsequent comparisons. A representative chromatogram obtained from a blank run is shown in Figure S27a. Comparison of the chromatogram of the OXCBZ reference material from Sigma-Aldrich UK with those of the powders used in sublimation experiments confirmed the presence of a chemical impurity (Table S6 and Figures S27b – S27e). The impurity had a retention time of ≈ 6.52 min in the liquid chromatograms with a corresponding *m/z* of 222.2 (Figure S29) and was found to be present in all of the analyzed samples, including the reference material from Sigma-Aldrich. The impurity was subsequently identified as DBZ. The molecular structure of DBZ is shown in Figure S30.

HPLC-MS indicated that the amount of DBZ was greater in powder samples of OXCBZ subjected to longer sublimation experiments, suggesting that prolonged exposure of OXCBZ to high temperature induces thermal degradation and a subsequent increase in the amount of DBZ.

HPLC-MS was additionally performed on solution-grown OXCBZ form III material and the starting OXCBZ material from Molekula that was used in solution crystallization experiments (Table S6 and Figure S28). The analysis confirmed the presence of DBZ in both types of material, with the concentration of the impurity found to be less in the solution-grown material than in OXCBZ samples from sublimation experiments. Whilst the presence of DBZ in the solution-grown material was verified, its significance on the emergence of twisting for both the solution- and sublimation-grown crystals of OXCBZ III remained unclear.

Table S6. Overview of HPLC analysis of OXCBZ starting material from Sigma-Aldrich UK and Molekula, OXCBZ starting powders subjected to sublimation and solution-grown OXCBZ III material. Retention times and peak areas corresponding to OXCBZ and DBZ are shown (n = 3). The actual concentration for each sample was 1 mg/mL.

Sample	Retention time of OXCBZ (min)	Peak area of OXCBZ (%)	Retention time of DBZ (min)	Peak area of DBZ (%)
**OXCBZ (Sigma-Aldrich UK)**	5.932 ± 0.002	81.18 ± 2.59	6.526 ± 0.002	1.811 ± 0.04
**OXCBZ – 16 hours of sublimation**	5.932 ± 0.005	79.53 ± 2.25	6.524 ± 0.005	3.047 ± 0.4
**OXCBZ – 24 hours of sublimation**	5.935 ± 0.001	78.68 ± 1.36	6.524 ± 0.001	4.323 ± 0.1
**OXCBZ – 48 hours of sublimation**	5.934 ± 0.002	81.11 ± 0.99	6.522 ± 0.003	6.601 ± 0.1
**OXCBZ (Molekula)**	5.935 ± 0.003	81.07 ± 0.64	6.524 ± 0.003	1.969 ± 0.04
**OXCBZ III (50:50 v/v Ethanol/Toluene)**	5.935 ± 0.001	75.86 ± 1.77	6.527 ± 0.0006	2.199 ± 0.02

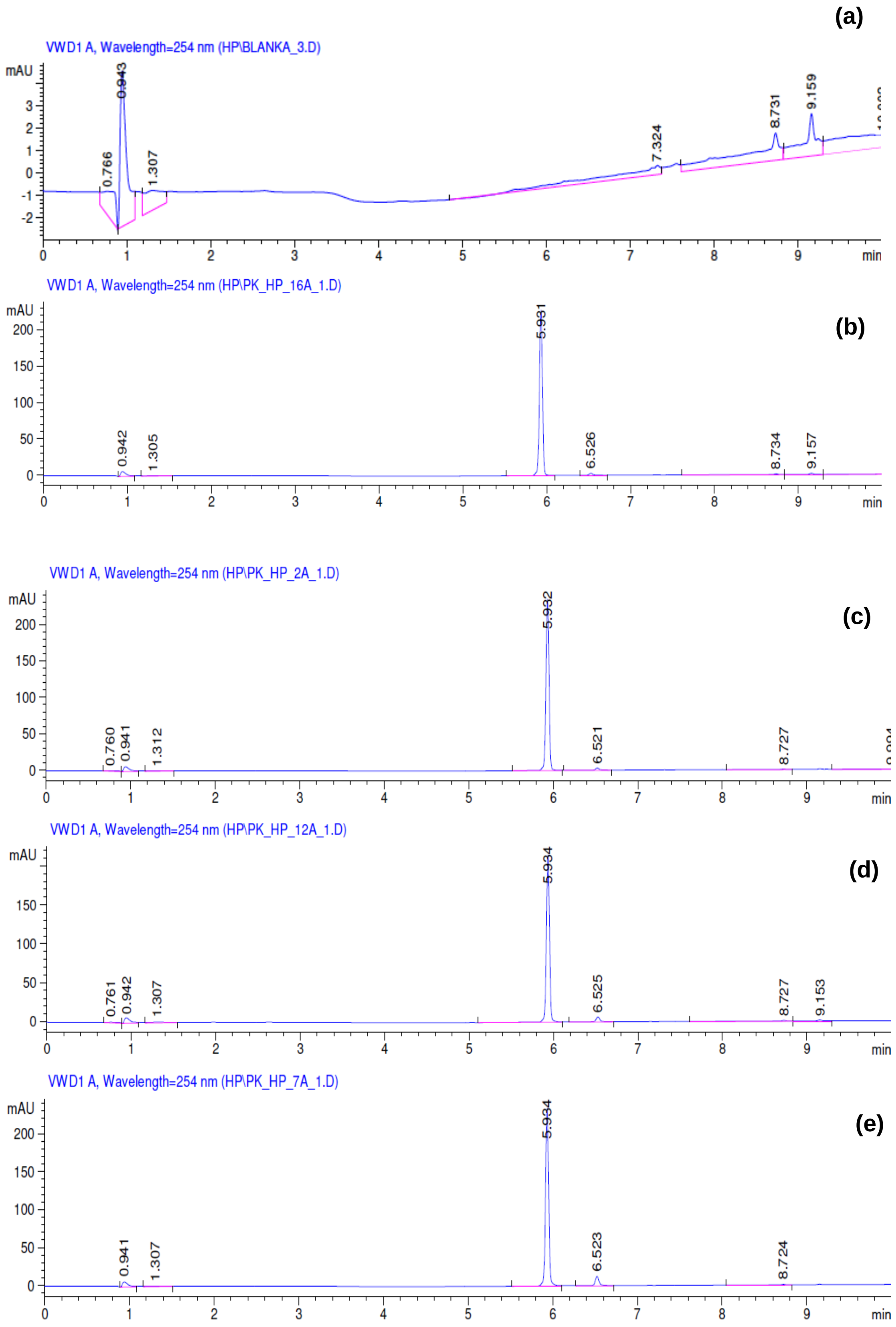

Figure S27. Representative UV chromatograms of (a) blank sample; (b) OXCBZ starting material as obtained from Sigma-Aldrich UK; (c) OXCBZ material following 16 hours of sublimation; (d) OXCBZ material following 24 hours of sublimation; and (e) OXCBZ material following 48 hours of sublimation.

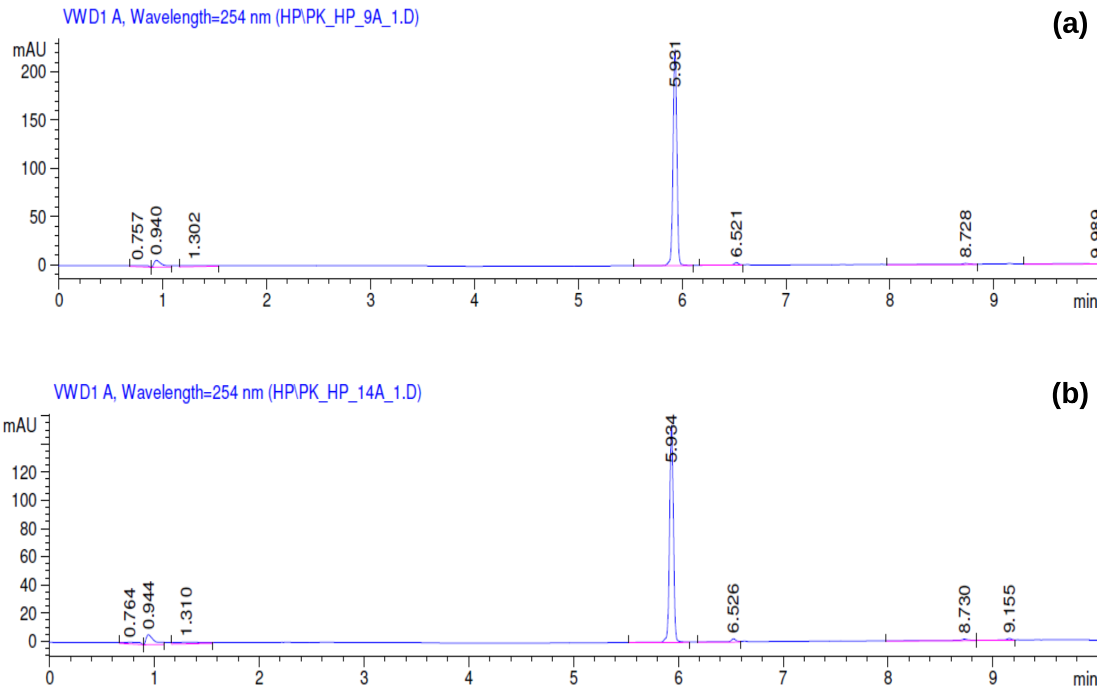

Figure S28. Representative UV chromatograms of (a) OXCBZ starting material obtained from Molekula and (b) OXCBZ form III material crystallized from 50:50 v/v ethanol/toluene.

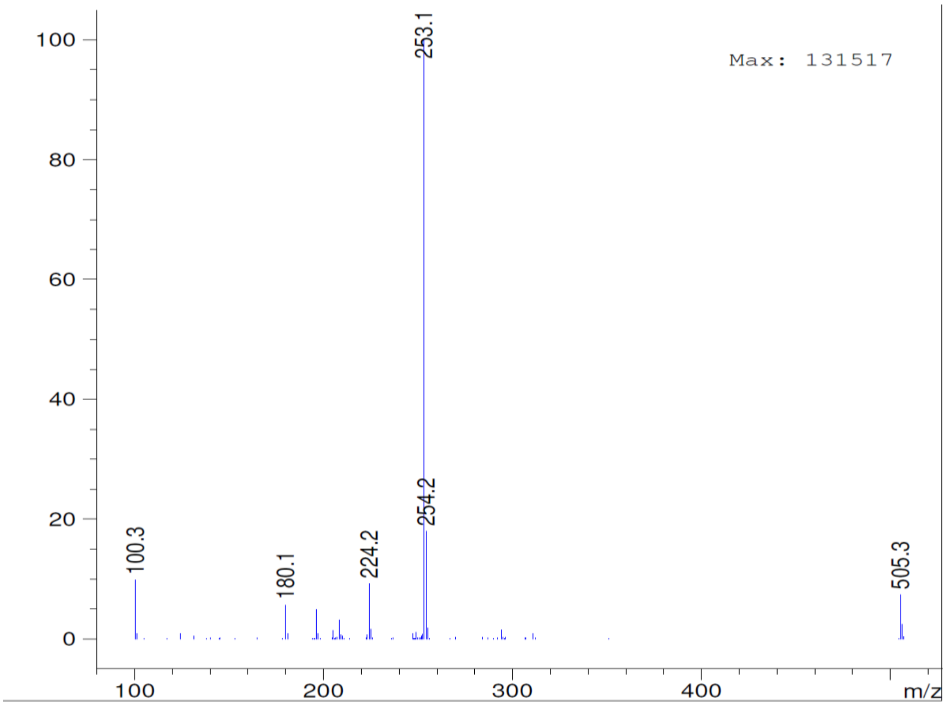

(a)

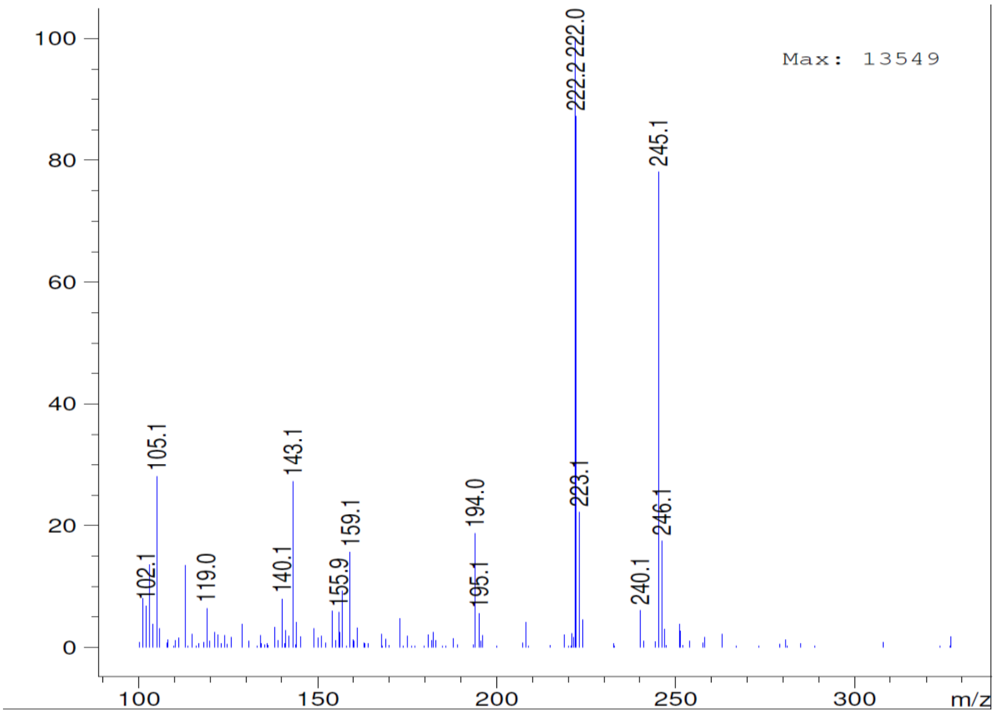

(b)

Figure S29. Representative mass spectra (a & b) corresponding to UV chromatogram peaks characteristic of OXCBZ and DBZ. Spectrum (a) corresponds to OXCBZ and spectrum (b) is representative of DBZ.

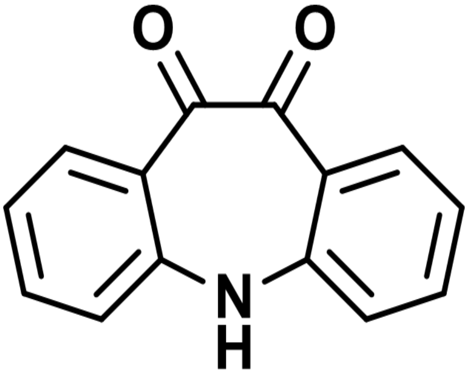

Figure S30. Molecular structure of DBZ (IUPAC name: 5*H*-dibenzo[*b*,*f*]azepine-10,11-dione, $C_{14}H_9NO_2$, molecular weight: 223.23).

II. Computational ESI

S10. Conformation Analysis

The two conformers of the isolated OXCBZ molecule were optimized using Gaussian09[17] at PBE0/6-31G(d,p) level (Figure S31). The conformations are defined by whether the -NH_2 of the amide group is on the same side (*syn*) or different side (*anti*) of the C=O kink on the azepine ring. Both OXCBZ forms I and II contain the *anti* conformer.

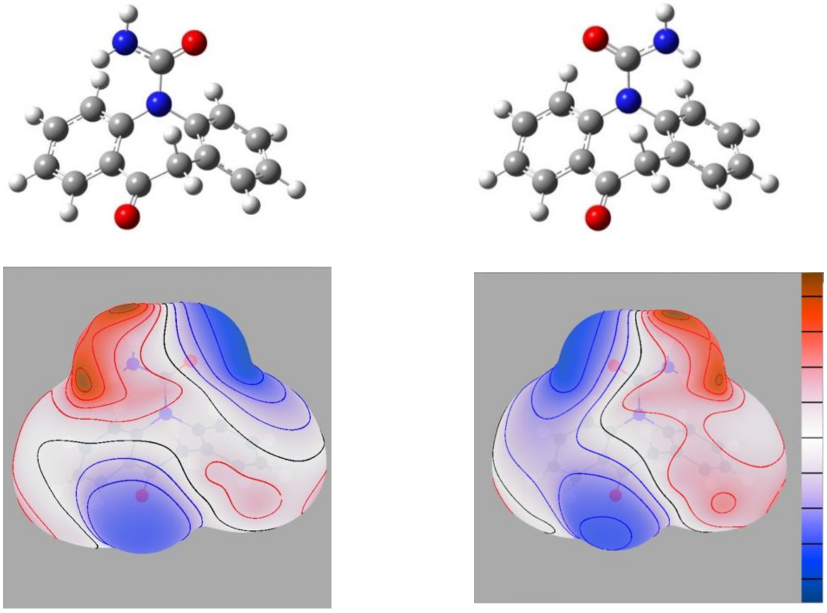

Figure S31. The *syn* (left) and *anti* (right) conformers of oxcarbazepine and the electrostatic potentials on the molecular surface defined by a 1.5 scaling of the van der Waals radii calculated from distributed multipoles representation of the PBE0/6-31G(d,p) charge densities. The electrostatic potential scale is from −1.40 eV (blue) to +1.40 eV (red).

The *anti* conformer is found to be rather more stable than the *syn* conformer by 4.3 kJ/mol (PBE0/6-31G(d,p)), because of the position of the C=O group. The electrostatic potential around the conformers differs significantly with the *anti* conformer having a larger dipole moment (Figure S31). The shapes of the two conformers are quite similar.

S11. Crystal Structure Prediction

Crystal structure prediction was carried out in the *anti* and *syn* conformation regions separately. As the *anti* conformer was seen in forms I and II, and is also more stable, one million (Z'=1) initial structures in the 60 most common space groups were generated in the *anti* search region, and half a million in the *syn* region.

CrystalPredictor2.1.0[18] was used for the generation and initial lattice energy minimizations of the structures, allowing the torsion angle connecting the amide group to the aromatic ring to change (Figure S32). Minimizations were performed with a dispersion-repulsion potential (FIT) of the *exp*-6 form,[19] and conformation-dependent atomic charges fitted to electrostatic potential calculated at PBE0/6-31G(d,p) level. The resulting unique structures were labelled by their energy ranking at this stage and conformation (a or s). This produced a set of 2500 *anti* and 1954 *syn* unique structures, spanning a lattice energy range of 23.6 kJ/mol.

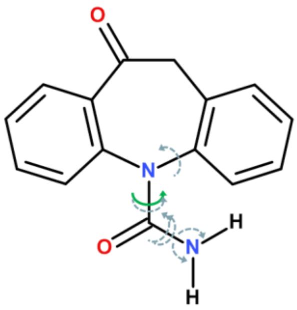

Figure S32. The flexibility of OXCBZ used in the CSP study. The green arrow denotes the only torsion angle varied in the CrystalPredictor search and the grey arrows denotes the additional torsion and bond angles refined by CrystalOptimizer.

CrystalOptimizer2.4[20] was used for further structural refinement, with the additional torsion angles and bond angles shown in Figure S32 allowed to adjust to the crystal packing forces. The intramolecular energy penalty was calculated from a grid of conformations, each optimized at PBE0/6-31G(d,p) level with the conformational variables in Figure S32 constrained and the others relaxed. The intermolecular lattice

energy optimizations were carried out with the FIT exp-6 potential, and the electrostatic contribution to the intermolecular lattice energy calculated from the distributed multipoles derived from PBE0/6-31G(d,p) charge density for the specific conformation. Clustering of the resulting lattice energy optimized crystal structures led to 1609 and 1094 structures in the *anti* and *syn* regions, of which 178 *anti* and 118 *syn* structures are within 15 kJ/mol of the global minimum. The global minimum (s59) was found in the *syn* region, with OXCBZ form I only 0.2 kJ/mol higher in lattice energy, as the lowest minimum in the *anti* region (structure a2) (Table S7 and Figure S33). Form II was found 2.7 kJ/mol higher than form I (structure a20).

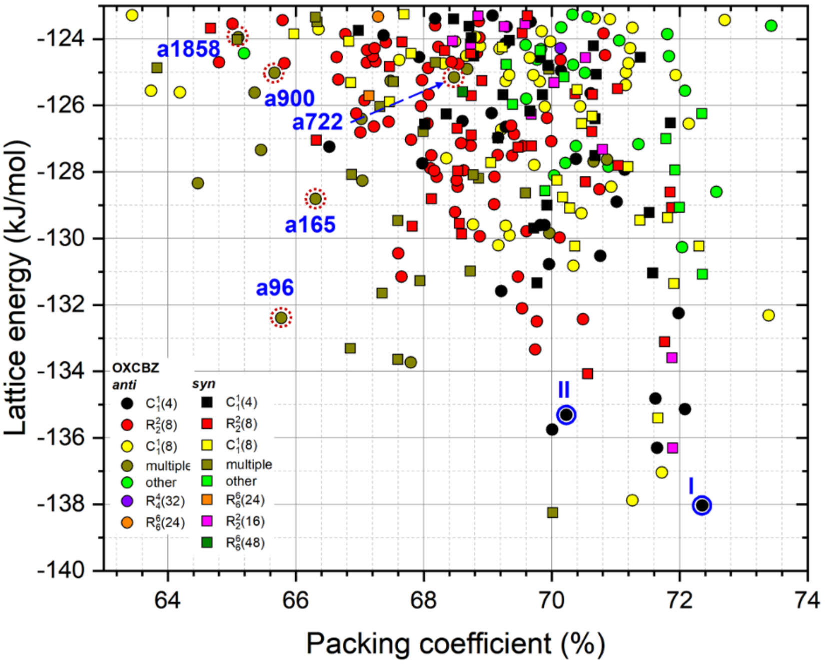

Figure S33. CrystalOptimizer lattice energy landscape of oxcarbazepine, combining the CSP searches in the *anti* (circle) and *syn* (square) regions. The colour denotes the hydrogen-bonding graph set calculated using *Mercury* 3.9 with “multiple” indicating multiple C-/R- motifs and “other” when there were no hydrogen bonds within the default geometric criteria. Packing coefficients were calculated with CCDC software *packingcoefficients* using 0.1 Å grid spacing. The candidate structures for new forms of OXCBZ are labelled as a# or s# with # referring to the ranking after CrystalPredictor. Forms I and II are labelled with blue circles.

With the additional carbonyl group on the aromatic ring, the OXCBZ search found many structures with hydrogen-bonding motifs other than those usually seen in the

carbamazepine family, i.e. amide C1,1(4) catemers or R2,2(8) dimers. OXCBZ form I was found amongst the lowest in lattice energy and the most densely packed structures. However, *syn* OXCBZ was able to form competitive structures despite being the less stable and less polar conformer (Figure S33).

Table S7. The CSP structures within 5 kJ/mol of form I, and the OXCBZ form III candidates. The .res files for the CSP structures are available from l.s.price@ucl.ac.uk on request.

	Space group	a (Å)	b (Å)	c (Å)	α (°)	β (°)	γ (°)	Density (g/cm^3)	ΔE_{latt}* (kJ/mol)
s59	P-1	11.9996	7.8518	8.8305	108.98	54.80	112.72	1.3485	-0.21
a2 (I)	$P2_1/c$	5.0425	9.7198	25.6229	90	75.08	90	1.3808	0.00
a57	$P2_1/a$	10.6544	16.7313	6.9012	90	88.11	90	1.3628	0.15
a18	$P2_1/a$	10.3087	16.4001	7.2371	90	92.87	90	1.3712	0.99
s1656	P-1	7.6510	8.2808	10.5596	87.57	75.91	69.05	1.3842	1.73
a5	Pbca	9.2836	10.0994	26.0999	90	90	90	1.3695	1.74
a19	$Pca2_1$	25.4293	5.0474	9.7248	90	90	90	1.3424	2.29
s3	P-1	8.7351	9.6422	8.3139	106.18	67.24	87.14	1.3730	2.63
a20 (II)	$P2_1$	12.5820	10.0222	4.9821	90	83.73	90	1.3416	2.73
a55	C2/c	30.3631	9.4043	10.0902	90	57.48	90	1.3795	2.90
a16	$P2_1/c$	13.1579	9.2952	10.0481	90	84.88	90	1.3689	3.22
s1103	$P2_1/n$	13.0375	7.3879	13.1376	90	77.64	90	1.3556	3.96
a3	C2/c	13.1183	11.5977	20.5935	90	124.69	90	1.3008	4.31
s15	$P2_1/n$	7.2107	10.4137	17.2438	90	89.54	90	1.2941	4.40
s7	$P2_1/c$	10.5792	15.8716	7.6109	90	73.03	90	1.3708	4.44
a22	$P2_1/a$	16.0894	7.157	11.177	90	78.58	90	1.3282	4.70
s68	P-1	10.7814	8.7534	7.6886	99.52	84.66	67.64	1.2849	4.73
s14	P-1	9.5435	7.8803	8.8325	79.72	72.11	76.32	1.3731	4.93

a96	R-3	36.0565	36.0565	5.3017	90	120	90	1.2632	5.64
a165	R-3	36.8890	36.8890	5.0353	90	120	90	1.2707	9.23
a722	$P6_1$	20.4388	20.4388	5.3061	90	90	120	1.3093	12.88
a900	R-3	36.4698	36.4698	5.1989	90	120	90	1.2591	13.02
a1858	P-3	21.3178	21.3178	5.1156	90	90	120	1.2484	14.09

*Relative to OXCBZ form I (a2) as calculated with CrystalOptimizer.

S12. Sensitivity of the Crystal Energy Landscape to Energy Models

The outcome of the CSP search is that OXCBZ can adopt a range of crystal structures, with two types of conformer, a variety of hydrogen bonding motifs and a significant variation in density, which are energetically competitive and within the plausible energy difference between polymorphs. Hence, we tested the sensitivity of the crystal energy models to the assumed energy model.

S12.1 Sensitivity to Polarization by the Environment

The sensitivity of the OXCBZ lattice energy landscape to the effect of the charge density changing on crystallization was estimated using a polarizable continuum model (PCM) with a representative dielectric constant (ε=3) for organic molecular crystals.[21] The CSP structures were re-optimized keeping the molecular conformations rigid with DMACRYS[19] using the FIT potential and distributed multipoles from PBE0/6-31G(d,p)/PCM wavefunctions (Figure S34), and the PCM conformational energy penalty used for the revised intermolecular energy penalty. This caused a significant reordering of the relative stabilities (Figure S34), mainly from changing the relative energies of different hydrogen bonding motifs. The PCM environment also reduces the energy difference between the *syn* and *anti* conformations to 3.7 kJ/mol, thus stabilizing the *syn* crystal structures by about 0.6 kJ/mol.

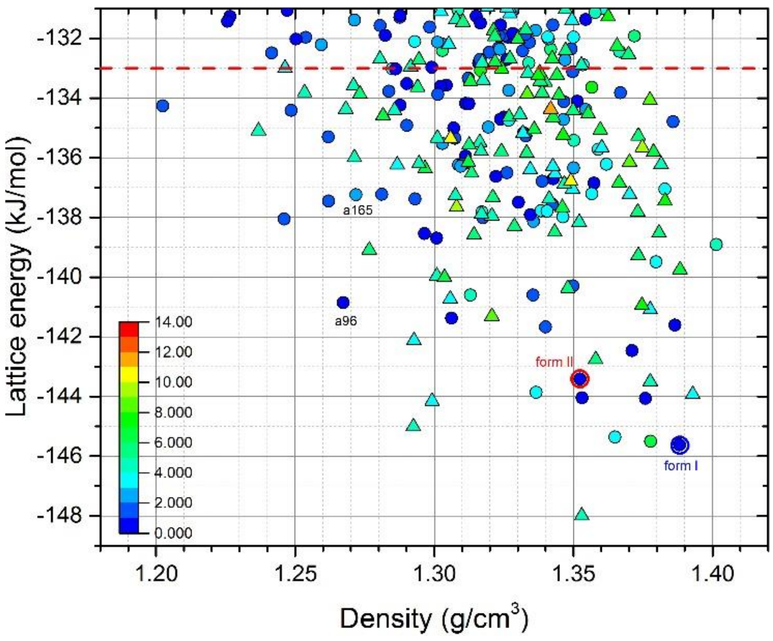

Figure S34. The PCM (ε=3) lattice energy landscape of oxcarbazepine with the conformational and intermolecular lattice energy recalculated using the PBE0/6-31G(d,p) charge density in a polarisable continuum model (PCM) with dielectric ε=3. The structures are colour-coded with relative intramolecular energies for PBE0/6-31G(d,p)/PCM(ε=3).

S12.2 Periodic DFT-D Lattice Energies

To further test the sensitivity of the relative energies, periodic DFT-D optimizations were carried out on a small number of OXCBZ observed and CSP generated structures with small unit cells, as unfortunately all candidates for OXCBZ form III have unit cells that are too big for periodic DFT-D calculations. The calculations were performed with CASTEP ver. 16.1.1,[22] using PBE functional and Tkatchenko-Scheffler's dispersion correction scheme.[23] Plane wave cutoff energy of 1100 eV, and Monkhorst-Pack *k*-point spacing of 0.05 $Å^{-1}$ were used to ensure basis set convergence and proper sampling of the Brillouin zone. Electronic energies were converged to 10^{-10} eV and structural optimizations were converged with residual force no larger than 0.001 eV/Å. Fine grid scale was set at 4.0. After optimizations, single-point energy calculations were performed on the optimized structures using other dispersion schemes, such as Grimme's D02 and D03,[24] and MBD*[25] schemes (Table S8).

The PBE-TS optimization improves the match between the structures a2 and a20 and the experimental structures, with a2 having an $RMSD_{20}$ = 0.214 Å with form I

(CANDUR01 determined at 95 K) and a20 a $RMSD_{20}$ = 0.304 Å with form II (CANDUR02 also 95 K). However, the relative energies were very sensitive to the dispersion correction used, emphasizing the role of the van der Waals forces and the variation in density between the structures (Table S8).

Table S8. PBE-TS optimized CSP structures and relative energies calculated for these these structures using different dispersion corrections.

	a (Å)	b (Å)	c (Å)	α (°)	β (°)	γ (°)	Volume ($Å^3$)	$ΔE_{latt}$ (kJ/mol)			
								PBE-TS	PBE-MBD*	PBE-D02	PBE-D03
a2 (I)	5.0472	9.3718	24.8188	90	96.63	90	1166.13	0.00	0.00	0.00	0.00
s59	11.8279	7.4599	8.5017	101.95	58.65	107.08	611.02	2.73	3.66	-1.09	-1.64
a20 (II)	4.9757	9.6983	12.4043	90	94.97	90	596.33	4.03	4.34	4.51	0.81
s68	10.5522	8.5724	7.4429	97.36	83.58	68.07	610.02	4.26	4.44	1.06	2.47

S12.3 Sensitivity to Temperature

A simple estimate of the effect of temperature on the relative energies of the CSP generated crystals was made by calculating the Helmholtz free energy (FE) at 298 K of each crystal structure using the harmonic rigid-body elastic constants and Γ-point phonons of the PCM distributed multipole lattice energy model. The energy ranking of the structures is changed by this crude approximation to the effects of temperature, (Figure S35) reducing the energy differences between form I and II and the less dense candidate structures for form III.

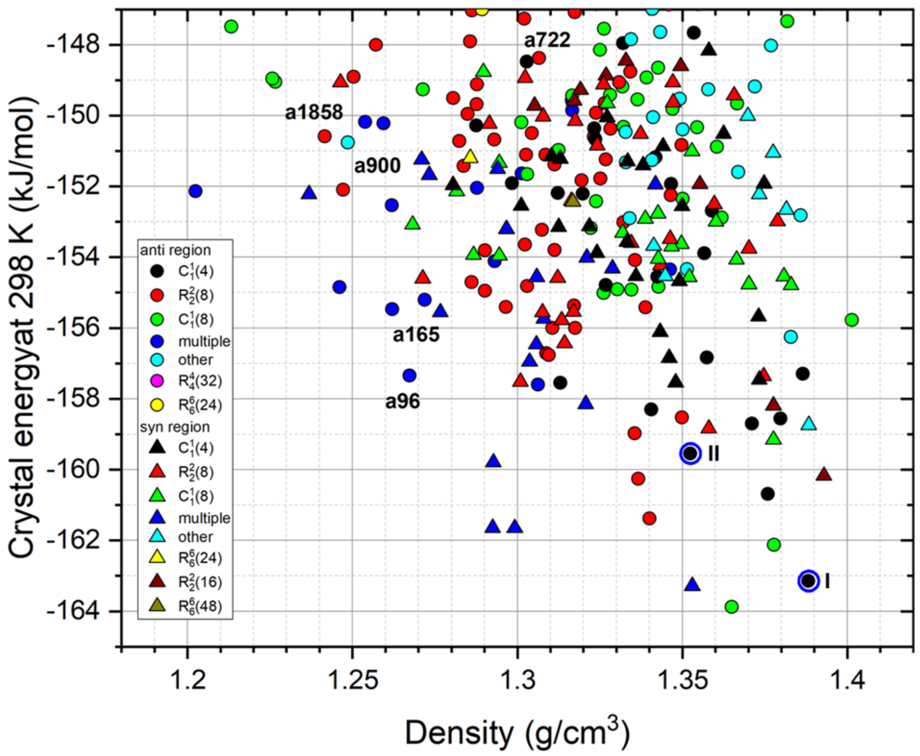

Figure S35. Free energy landscape of oxcarbazepine at 298 K, calculated with PBE0/6-31G(d,p)/PCM(ε=3) and rigid-molecule lattice phonon vibrational correction.

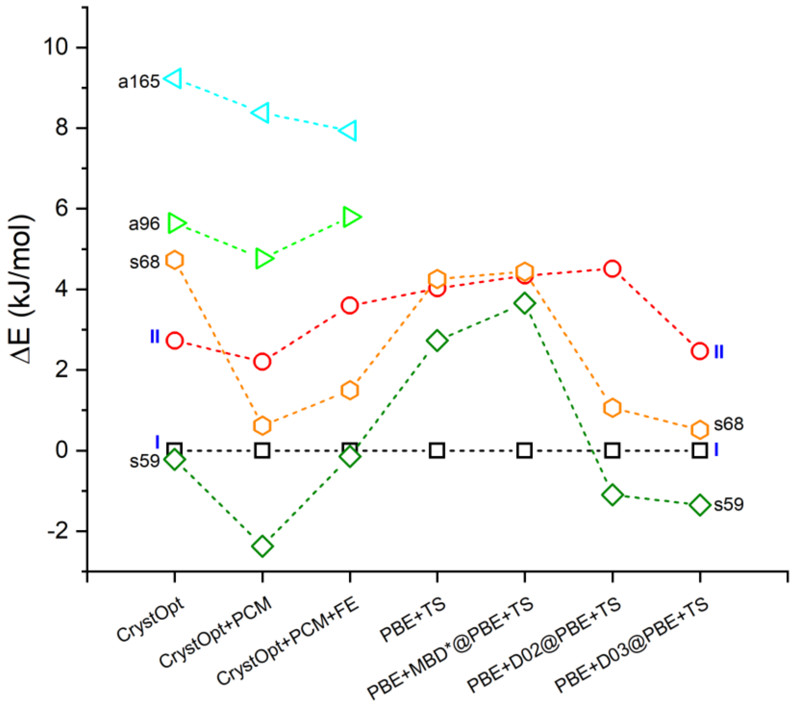

Figure S36. Comparison of relative energies of a selection of known and CSP structures of OXCBZ.

The overall comparison of the relative energies of the CSP crystal structures that match forms I (a2) and II (a20), the original global minimum, s59 and the structures that match the novel XRPD patterns (a96 and a165 for Form III) show significant variation with methods and the inclusion of temperature effects (Figure S36). However, the variation is within the energy range of plausible polymorphism, though the new forms are calculated to be metastable.

S13. Calculated Properties of the CSP candidates for OXCBZ III

S13.1 XRPD Patterns, Structures and Similarities

Five CSP structures, a96, a165, a722, a900 and a1858, were found to have XRPD patterns similar to those observed experimentally (Figure S37and Table S9) and those reported by Lutker and Matzger.[26] Of these, a96 is likely to be within the energy range of plausible polymorphs, with a165 less stable, and the other three CSP structures considerably higher in energy (Figure S35).

Among the five candidates, a96, a165 and a900 are in *R*-3 space group, with a165 loosely isomorphous to CBZ II, matching 15 out of a 15-molecule cluster, if the default similarity criteria of *Mercury* were lowered to 40% difference in distances and 40° in angles. a1858 is in *P*-3 group, while a722 is in hexagonal $P6_1$ group. However, these structures have similarities to each other matching 18 or more molecules within a 30-molecule cluster, for a96, a900 and a1858, or between a165 and a1858 (Table S9). There is less similarity between a722 and the other 4 candidate structures, and hence this structure was not discussed in the manuscript Figure 11.

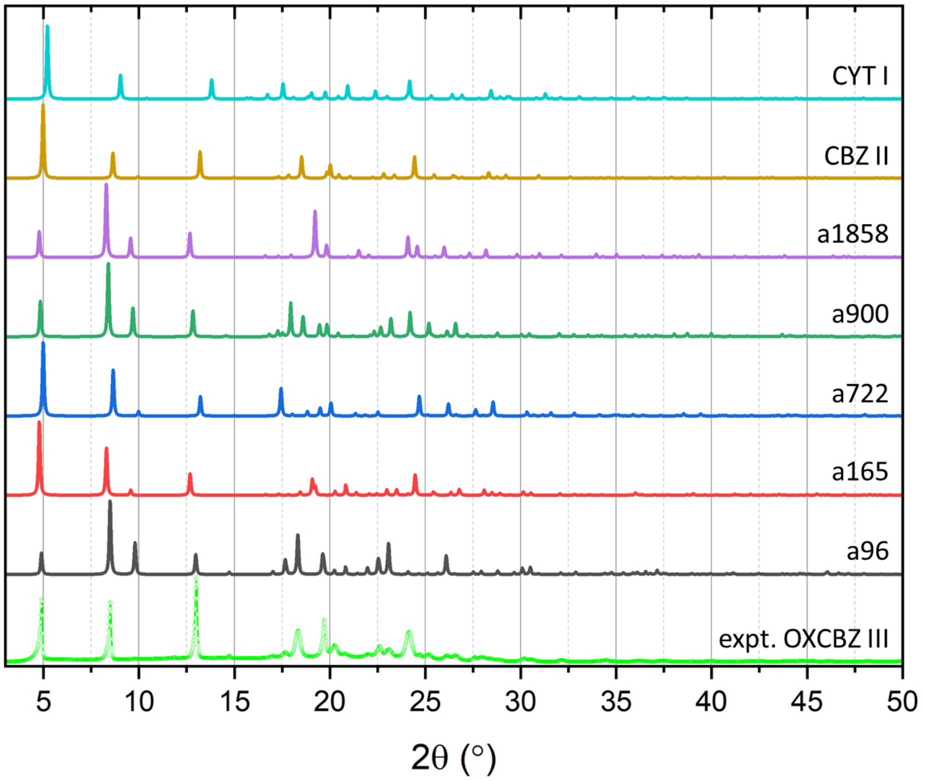

Figure S37. Comparison of simulated XRPD patterns of five CSP candidates for OXCBZ form III and those of CBZ II (CBMPZN03) and CYT I (SOGLEG) with the experimental XRPD of OXCBZ III

Table S9. The number of molecules in a 30-molecule cluster that can be overlaid using Mercury Crystal Packing Similarity within Mercury.

	**a96**	**a1858**	**a900**	**a165**	**a722**
**a96**	-	21/30	19/30	12/30	7/30
**a1858**	21/30	-	20/30	21/30	11/30
**a900**	19/30	20/30	-	14/30	16/30
**a165**	12/30	21/30	14/30	-	17/30
**a722**	7/30	11/30	16/30	17/30	-

S13.2 Channel Properties

Table S100. Void properties and cell parameters of OXCBZ form III candidates, compared to those of CBZ II and CYT I. For ease of comparison, the cell volumes and void volumes of a722 and a1858 are tripled in parenthesis to have the same number of molecules as the *R-3* structures.

α=β=90° γ=120°	Space Group	a=b (Å)	c (Å)	Volume ($Å^3$)	Void[1] volume ($Å^3$)	Effective Void[1] diameter (Å)	$ΔE_{latt}$[2] (kJ/mol)
OXCBZ a96	*R*-3	36.0565	5.3017	5969.16	226.82	4.26	5.64
OXCBZ a165	*R*-3	36.8890	5.0353	5934.03	387.64	5.72	9.23
OXCBZ a722	$P6_1$	20.4388	5.3061	1919.63 (5758.89)	146.10 (438.30)	5.92	12.88
OXCBZ a900	*R*-3	36.4698	5.1989	5988.37	268.08	4.68	13.02
OXCBZ a1858	*P*-3	21.3178	5.1156	2013.32 (6039.96)	138.27 (414.81)	5.87	14.09
CBZ II (CBMZPN03)	*R*-3	35.454	5.253	5718.32	522.70	6.50	-
CYT I (SOGLEG)	*R*-3	33.9078	5.6754	5651.01	375.53	5.30	-

[1] Void space calculated as contact surfaces using a probe of radius 1.2 Å and a grid spacing of 0.7 Å with the Voids tool of Mercury ver. 3.9. Effective diameters were calculated with the channels treated as circular cylinders. [2]Relative lattice energy to OXCBZ form I (a2) as calculated with CrystalOptimizer.

All 5 candidate structures have void channels (Figure S38). Although a722 does not have the same channel construct as those in the trigonal structures, it has a similar construct of inter-channel packing. The diameters of void channels in a165, a722, a900 and a1858 are only slightly smaller than that of CBZ II which experimentally has been observed to contain molecules as large as toluene,[27] therefore these structures may be able to accommodate small solvent molecules which would stabilize the structures. The

channels have a propeller-like directionality from the arched 2H-dibenzazepine rings: i.e. of the two OXCBZ phenyl rings, only those on the $-NH_2$ side of the amide group line the hydrophobic interior walls of the channel.

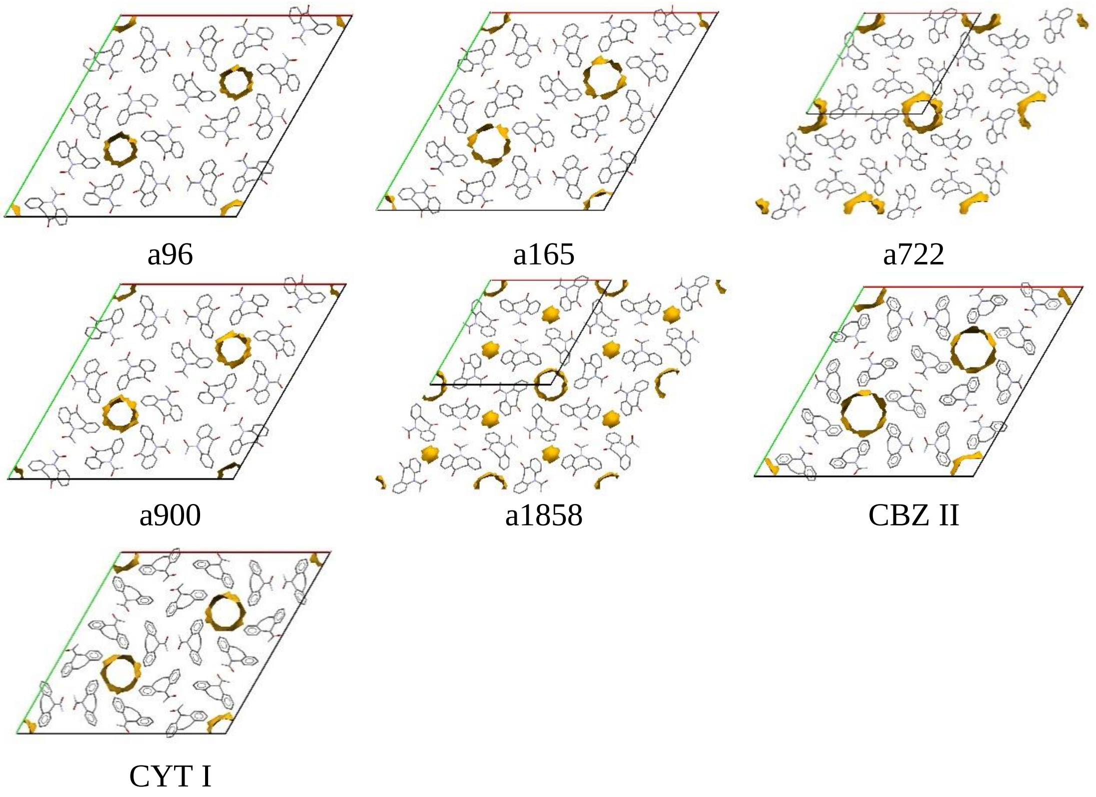

Figure S38. Void channels in OXCBZ CSP candidates, compared to those in CBZ II, CYT I.

S13.3 Calculated Growth and BFDH Morphology of OXCBZ III Candidates

Geometry-based BFDH morphology of all OXCBZ III candidates, using CCDC Mercury, show that all will grow into hexagon-shaped needles, with similar dominant faces on the sides (Figure S39). These BFDH calculated morphologies are in good agreement with the more elaborate growth morphology calculations for a96 and a165, which uses the forces between the molecules, here calculated from the ESP atomic charges and the FIT potential (i.e. the same intermolecular potential that was used for the CrystalPredictor search). The sides of channels are the slowest growing faces, while the ends channels have the fastest growth rate. In contrast, the growth and BFDH morphologies of form I and II of OXCBZ are more block-like (Figure S40).

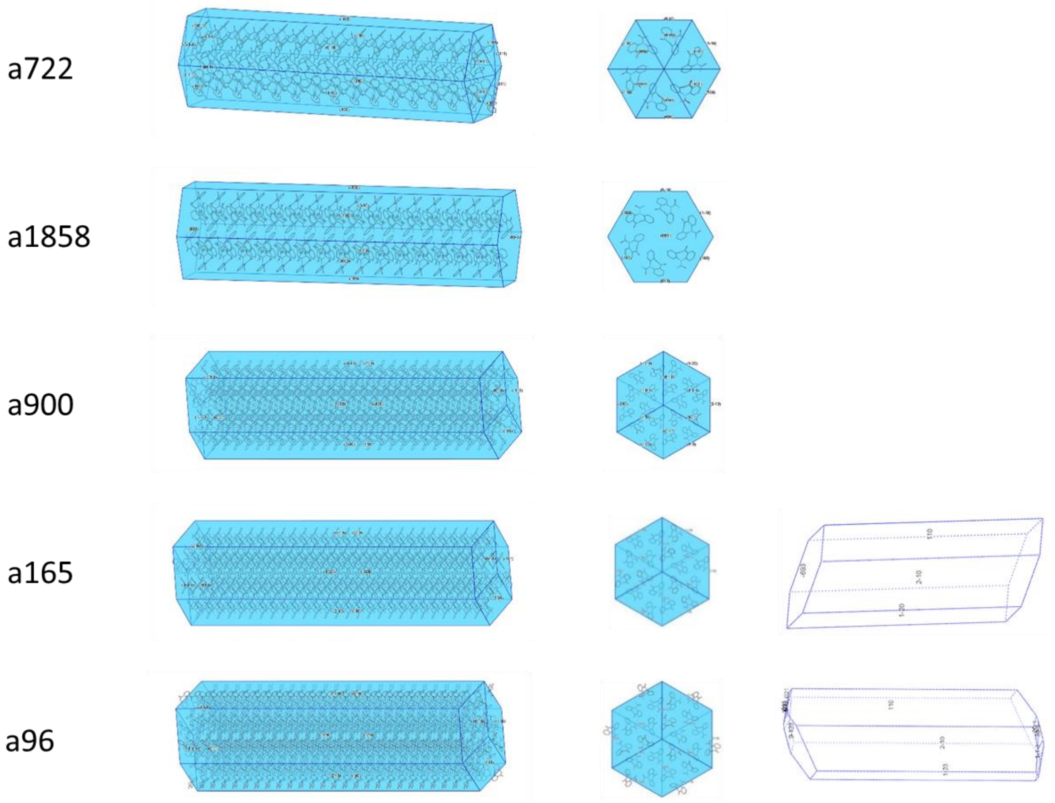

Figure S39. BFDH morphologies of OXCBZ form III candidates a96, a165, a722, a900, a1858, also attachment-energy-based growth morphologies for a96 and a165.

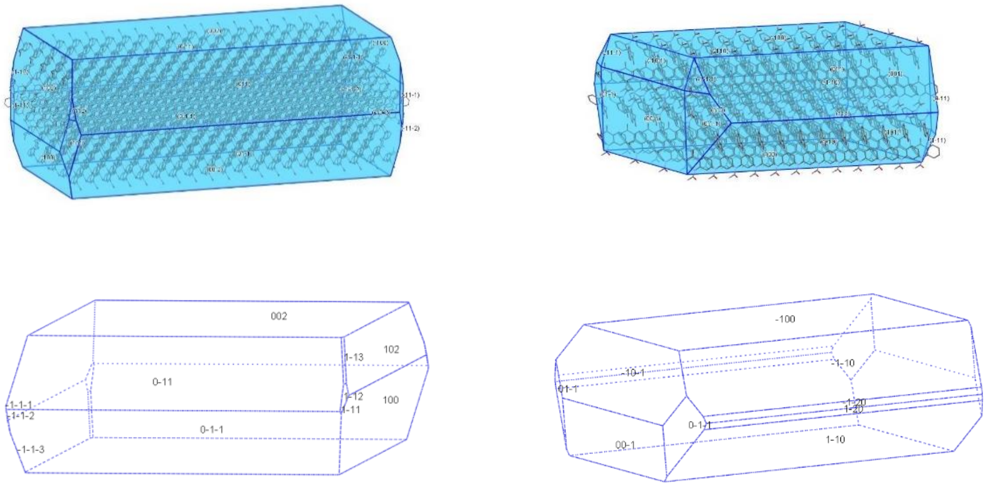

Figure S40. BFDH (top row) and growth (bottom row) morphology of OXCBZ form I (left column) and II (right column).

References

1. Shard, A.G.; Havelund, R.; Spencer, S. J.; Gilmore, I. S.; Alexander, M. R.; Angerer, T. B.; Aoyagi, S.; Barnes, J.-P.; Benayad, A.; Bernasik, A.; Ceccone, G.; Counsell, J. D. P.; Deeks, C.; Fletcher, J. S.; Graham, D. J.; Heuser, C.; Lee, T. G.; Marie, C.; Marzec, M. M.; Mishra, G.; Rading, D.; Renault, O.; Scurr, D. J.; Shon, H. K.; Spampinato, V.; Tian, H.; Wang, F.; Winograd, N.; Wu, K.; Wucher, A.; Zhou, Y.; Zhu, Z. Measuring Compositions in Organic Depth Profiling: Results from a VAMAS Interlaboratory Study. *J. Phys. Chem. B* **2015,** *119*, 10784-10797.
2. Abràmoff, M.D.; Magalhães, P. J.; Ram, S.J. Image Processing with ImageJ. *Biophotonics Int.* **2004,** *11*, 36-42.
3. Fang, L.; Feng, L.; Huixian, G.; Chao, C.; Bo, L.; Yu, W.; Huajun, L. Comparison and Analysis of Twist Pitch Length Test Methods for ITER Nb_3Sn and NbTi Strands. *Rare Metal Mat. Eng.* **2015,** *44*, 2095-2099.
4. Pawley, G. Unit-cell refinement from powder diffraction scans. *J. Appl. Crystallogr.* **1981,** *14*, 357-361.
5. Coelho, A.A. *TOPAS* and *TOPAS-Academic*: an optimization program integrating computer algebra and crystallographic objects written in C++. *J. Appl. Crystallogr.* **2018,** *51*, 210-218.
6. Faudone, S.N.; Paschoal, A.R.; Carvalho Jr., P.S.; Ellena, J.; Terra Martins, F.; Cuffini, S.L.; Ayala, A.P.; Sperandeo, N.R. X-ray diffraction, vibrational and thermal study of dibenzazepinodione, a pharmacopeial impurity of oxcarbazepine. *J. Mol. Struct.* **2019,** *1182*, 204-212.
7. David, W.I.F.; Shankland, K. Structure determination from powder diffraction data. *Acta Crystallogr. A* **2008,** *64*, 52-64.
8. David, W.I.F.; Shankland, K.; van de Streek, J.; Pidcock, E.; Motherwell, W.D.S.; Cole, J.C. *DASH*: a program for crystal structure determination from powder diffraction data. *J. Appl. Crystallogr.* **2006,** *39*, 910-915.
9. Kabova, E.A.; Cole, J.C.; Korb, O.; Williams, A.C.; Shankland, K. Improved crystal structure solution from powder diffraction data by the use of conformational information. *J. Appl. Crystallogr.* **2017,** *50*, 1421-1427.
10. Rietveld, H. Line profiles of neutron powder-diffraction peaks for structure refinement. *Acta Crystallogr.* **1967,** *22*, 151-152.

11. Rietveld, H. A profile refinement method for nuclear and magnetic structures. *J. Appl. Crystallogr.* **1969,** *2*, 65-71.
12. Burger, A.; Ramberger, R. On the polymorphism of pharmaceuticals and other molecular crystals. I. *Microchim. Acta* **1979,** *72*, 259-271.
13. Burger, A.; Ramberger, R. On the polymorphism of pharmaceuticals and other molecular crystals. II. *Microchim. Acta* **1979,** *72*, 273−316.
14. Brown, M.E. (2001). Purity Determination Using DSC. In: *Introduction to Thermal Analysis, Techniques and Applications, Second Edition*. pp 215-227. Kluwer Academic Publishers, Dordrecht.
15. Censi, R.; di Martino, P. Polymorph Impact on the Bioavailability and Stability of Poorly Soluble Drugs. *Molecules* **2015,** *20*, 18759-18776.
16. Amidon, G.L.; Lennernäs, H.; Shah, V.P.; Crison, J.R. A Theoretical Basis for a Biopharmaceutic Drug Classification: The Correlation of in Vitro Drug Product Dissolution and in Vivo Bioavailability. *Pharm. Res.* **1995,** *12*, 413-420.
17. Frisch, M. J.; Trucks, G. W.; Schlegel, H. B.; Scuseria, G. E.; Robb, M. A.; Cheeseman, J. R.; Scalmani, G.; Barone, V.; Mennucci, B.; Petersson, G. A.; Nakatsuji, H.; Caricato, M.; Li, X.; Hratchian, H. P.; Izmaylov, A. F.; Bloino, J.; Zheng, G.; Sonnenberg, J. L.; Hada, M.; Ehara, M.; Toyota, K.; Fukuda, R.; Hasegawa, J.; Ishida, M.; Nakajima, T.; Honda, Y.; Kitao, O.; Nakai, H.; Vreven, T.; Montgomery, J. A., Jr; Peralta, J. E.; Ogliaro, F.; Bearpark, M.; Heyd, J. J.; Brothers, E.; Kudin, K. N.; Staroverov, V. N.; Kobayashi, R.; Normand, J.; Raghavachari, K.; Rendell, A.; Burant, J. C.; Iyengar, S. S.; Tomasi, J.; Cossi, M.; Rega, N.; Millam, J. M.; Klene, M.; Knox, J. E.; Cross, J. B.; Bakken, V.; Adamo, C.; Jaramillo, J.; Gomperts, R.; Stratmann, R. E.; Yazyev, O.; Austin, A. J.; Cammi, R.; Pomelli, C.; Ochterski, J. W.; Martin, R. L.; Morokuma, K.; Zakrzewski, V. G.; Voth, G. A.; Salvador, P.; Dannenberg, J. J.; Dapprich, S.; Daniels, A. D.; Farkas, Ö.; Foresman, J. B.; Ortiz, J. V.; Cioslowski, J.; Fox, D. J. *Gaussian 09, Revision D.01*, 2009.
18. Karamertzanis, P. G.; Pantelides, C. C. Ab initio crystal structure prediction - I. Rigid molecules. *J. Comput. Chem.* **2005,** *26*, 304-324.
19. Price, S. L.; Leslie, M.; Welch, G. W. A.; Habgood, M.; Price, L. S.; Karamertzanis, P. G.; Day, G. M. Modelling organic crystal structures using distributed multipole and polarizability-based model intermolecular potentials. *Phys. Chem. Chem. Phys.* **2010,** *12*, 8478-8490.

20. Kazantsev, A. V.; Karamertzanis, P. G.; Adjiman, C. S.; Pantelides, C. C. Efficient Handling of Molecular Flexibility in Lattice Energy Minimization of Organic Crystals. *J. Chem. Theory Comput.* **2011,** *7*, 1998-2016.
21. Cooper, T. G.; Hejczyk, K. E.; Jones, W.; Day, G. M. Molecular Polarization Effects on the Relative Energies of the Real and Putative Crystal Structures of Valine. *J. Chem. Theory Comput.* **2008,** *4*, 1795-1805.
22. Clark, S. J.; Segall, M. D.; Pickard, C. J.; Hasnip, P. J.; Probert, M. J.; Refson, K.; Payne, M. C. First principles methods using CASTEP. *Z. Kristallogr. Cryst. Mater.* **2005,** *220*, 567-570.
23. Tkatchenko, A.; Scheffler, M. Accurate Molecular Van Der Waals Interactions from Ground-State Electron Density and Free-Atom Reference Data. *Phys. Rev. Lett.* **2009,** *102*, 073005.
24. Grimme, S. Semiempirical GGA-type density functional constructed with a long-range dispersion correction. *J. Comput. Chem.* **2006,** *27*, 1787-1799.
25. Tkatchenko, A.; Alfe, D.; Kim, K. S. First-Principles Modeling of Non-Covalent Interactions in Supramolecular Systems: The Role of Many-Body Effects. *J. Chem. Theory Comput.***2012,** *8*, 4317-4322.
26. Lutker, K. M.; Matzger, A. J. Crystal polymorphism in a carbamazepine derivative: Oxcarbazepine. *J.Pharm. Sci.* **2010,** *99*, 794-803.
27. Cruz Cabeza, A. J.; Day, G. M.; Motherwell, W. D. S.; Jones, W. Solvent inclusion in form II carbamazepine. *Chem. Commun.* **2007,** 2007, 1600-1602.
